# Unusual highly diastereoselective Rh(II)-catalyzed dimerization of 3-diazo-2-arylidenesuccinimides provides access to a new dibenzazulene scaffold

**DOI:** 10.3762/bjoc.18.55

**Published:** 2022-05-11

**Authors:** Anastasia Vepreva, Alexander S Bunev, Andrey Yu Kudinov, Grigory Kantin, Mikhail Krasavin, Dmitry Dar’in

**Affiliations:** 1 Saint Petersburg State University, Saint Petersburg 199034, Russian Federation; 2 Medicinal Chemistry Center, Togliatti State University, 445020 Togliatti, Russian Federation; 3 Immanuel Kant Baltic Federal University, Kaliningrad 236041, Russian Federation

**Keywords:** diazo arylidene succinimides, dibenzoazulenodipyrroles, indenopyrroles, Rh_2_(esp)_2_-catalyzed decomposition, unsymmetrical dimers

## Abstract

Formation of unusual unsymmetrical dimers or/and indenes via Rh_2_(esp)_2_-catalyzed decomposition of 3-diazo-2-arylidenesuccinimides has been investigated. The reaction proceeded under mild conditions, and its result was shown to strongly depend on the nature of the substituents in the diazo substrate. The new reaction provides access to dibenzoazulenodipyrrole and indenopyrrole derivatives in moderate to high yield. Dibenzoazulenodipyrroles bearing alkyl substituents at the nitrogen atom showed pronounced cytotoxocity against the A549 human lung adenocarcinoma cell line while *N*-aryl analogs were non-cytotoxic.

## Introduction

3-Diazo-2-arylidenesuccinimides **1** (DAS) are heterocyclic vinyl-substituted diazocarbonyl compounds with an exclusively (*E*)-configured double bond which positions the aryl substituent and the diazo group in close proximity. The reactivity of DAS is twofold: on the one hand, these compounds undergo reactions which are typical for diazocarbonyl compounds, on the other hand, their specific geometry enables intriguing transformations with simultaneous involvement of the diazo function and the benzylidene fragment.

**Figure 1:**
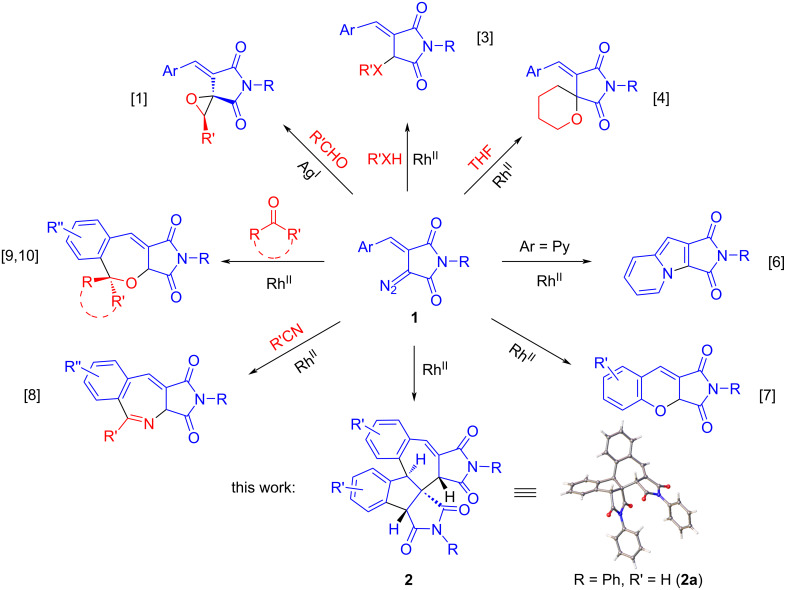
Previously reported transformations of DAS (**1**) and their unusual dimerization investigated in this work.

DAS were first described in 2020 and were involved in a [2 + 1] cycloaddition to aldehydes to give oxiranes [1]. Later on, we developed a convenient method for the preparation of this class of compounds [2] and showed that DAS can undergo Rh(II)-catalyzed insertion reactions into the heteroatom–H bonds [3]. In 2020, it was shown that under Rh(II) catalysis, DAS can enter insertion reactions into the C–O bond of ethers [4], a rare transformation for diazocarbonyl compounds [5]. The close spatial arrangement of the conjugate aryl fragment and the diazo group in the DAS molecule favors both intramolecular and intermolecular cyclizations involving the arylidene group. Thus, under catalytic decomposition, DAS containing a 2-pyridyl or 2-hydroxyaryl substituent at the double bond underwent cyclization as the result of intramolecular interception of the rhodium carbene, which led to the formation of indolizine [6] or 2*H*-chromene [7] derivatives, respectively (Figure 1). At the same time, Rh_2_(esp)_2_-catalyzed reactions of DAS with nitriles and carbonyl compounds (aldehydes and ketones) enable to obtain the products of formal [5 + 2] cycloaddition reactions ‒ 2-benzazepines [8] and 2-benzoxepines [9–10]. When studying the Rh(II)-catalyzed reaction of DAS with ketones [10], we attempted to involve poorly reactive fluorenone in the reaction. To our surprise, the formation of the spirocyclic benzoxepine was not observed. Instead, the principal product of the reaction was compound **2** (as unequivocally confirmed by the single-crystal X-ray analysis of a representative, compound **2a**, see Supporting Information File 1), presumably resulting from the dimerization of the DAS molecule (Figure 1). Intrigued by this unexpected course of the reaction and also by the unusual structure of compound **2**, we set off to study this transformation. Herein, we present the results obtained in the course of this investigation.

## Results and Discussion

The decomposition reaction of DAS **1a** in dichloromethane in the presence of Rh_2_(esp)_2_ (0.1 mol %) led to the formation of a mixture of the major product ‒ dimer **2a**, and minor indene **3a** (Table 1, entry 1). The target dimer was isolated in 74% yield as a single diastereomer. Its structure was reliably confirmed by X-ray analysis data (see Supporting Information File 1). Given that the main product **2a** is most likely the result of a bimolecular process while indene **3a** was formed via an intramolecular transformation, we attempted to completely switch the direction of the reaction in order to achieve an indene to form as the main product. However, carrying out the reaction under tenfold dilution did not fully suppress the bimolecular process to give indene **3a** in an acceptable yield, although its content in the reaction mixture increased significantly (Table 1, entry 1).

**Table 1:** Transformation of DAS **1** under Rh(II)-catalyzed decomposition.^a^

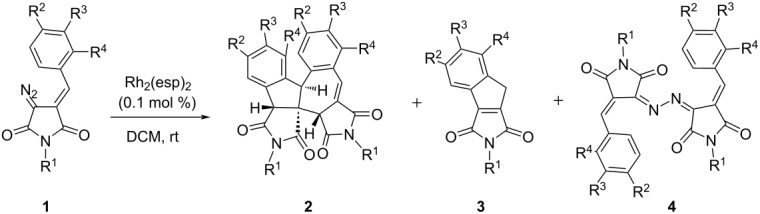						

Entry	Compounds	R^1^	R^2^, R^3^, R^4^	Yield of **2**^b^ (%)	Yield of **3** (%)	Yield of **4** (%)

1	**1**/**2**/**3**/**4a**	Ph	H, H, H	74 40^c^	(10)^d^ 15^c^ (42)	–
2	**1**/**2**/**3**/**4b**	4-MeOC_6_H_4_	H, H, H	73	–	–
3	**1**/**2**/**3**/**4c**	4-CF_3_C_6_H_4_	H, H, H	74	(6)	–
4	**1**/**2**/**3**/**4d**	4-MeC_6_H_4_	H, H, H	63	(13)	–
5	**1**/**2**/**3**/**4e**	4-FC_6_H_4_	H, H, H	86	–	–
6	**1**/**2**/**3**/**4f**	Bn	H, H, H	68	(14)	(10)
7	**1**/**2**/**3**/**4g**	Ph	MeO, H, H	traces	–	–
8	**1**/**2**/**3**/**4h**	Ph	Me, H, H	93	–	–
9	**1**/**2**/**3**/**4i**	Ph	CF_3_, H, H	18	–	(20)
10	**1**/**2**/**3**/**4j**	Ph	H, H, F	60	(12)	(18)
11	**1**/**2**/**3**/**4k**	Bn	F, H, H	42	–	18 (21)
12	**1**/**2**/**3**/**4l**	*c*-Pr	F, H, H	43	–	(21)
13	**1**/**2**/**3**/**4m**	iBu	Cl, H, H	52	(7)	(12)
14	**1**/**2**/**3**/**4n**	Ph	H, MeO, H	12	56^e^	–
15	**1**/**2**/**3**/**4o**	2-ClC_6_H_4_	H, H, H	–	35	–
16	**1**/**2**/**3**/**4p**	2-MeO-5-ClC_6_H_3_	H, H, H	–	34	–
17	**1**/**2**/**3**/**4q**	2-CO_2_EtC_6_H_4_	H, H, H	–	54	–

^a^Reaction conditions: 0.25 M solution of DAS **1** in DCM; 0.5 or 1.0 mmol scale. ^b^Isolated yields. ^c^Reaction was run under tenfold dilution. ^d^NMR yields are shown in parentheses. ^e^1.8:1.0 mixture of regioisomers was obtained (ratio 1.8:1).

Next, we investigated the influence of substituents in the DAS component **1** on the outcome of the Rh(II)-catalyzed decomposition. The results obtained clearly demonstrate that the nature of the substituent at the nitrogen atom in the DAS molecule has a minor effect: the substrates bearing both donor- and acceptor-substituted aryl groups form dimers **2** in high yields (Table 1, entries 2–5). In the case of *N*-benzyl-substituted DAS, byproduct azine **4** was detected by NMR analysis (Table 1, entry 6). The formation of these byproducts was also observed in the case of other DAS bearing alkyl groups at the nitrogen atom (Table 1, entries 10–13) as well as in the case of DAS **1i** containing a trifluoromethyl group in the arylidene ring (Table 1, entry 9). In the latter case, the negative effect of the presence of the electron-withdrawing group also manifested itself in a significant decrease in the yield of the target dimer **2** due to the formation of unidentified impurities. In one case, azine **4k** was isolated in pure form and characterized. It should be noted that such azines are typically observed as byproducts in Rh(II)-catalyzed reactions of diazocarbonyl compounds [11–14].

The introduction of a strong donor substituent (MeO) in position 4 of the benzylidene fragment (**1g**) led to the deactivation of the diazo substrate ‒ after 1 hour the conversion did not exceed 30% and even after 3 days the starting DAS **1g** was still present in the mixture. At the same time, only a trace amount of the expected dimerization product **2g** was detected along unidentified byproducts.

For the 3-methoxy-substituted DAS **1n**, the main reaction outcome was the unimolecular cyclization, leading to a mixture of regioisomeric indenes (Table 1, entry 14) while the target dimer **2n** was obtained in low (12%) yield. A similar result was obtained earlier in the study of DAS in the reaction with nitriles [8].

An unexpected and interesting result was the exclusive formation of indenes **3** during the catalytic decomposition of DAS containing an *ortho*-substituted phenyl group at the nitrogen atom (Table 1, entries 15–17). In all three cases, compounds **3** were obtained in moderate yields, while the formation of the corresponding dimers **2** or azines **4** was not observed. Most likely, the introduction of the *ortho*-substituent disturbed the molecule’s coplanarity, which may have created steric obstacles for the intermolecular process.

**Scheme 1:**
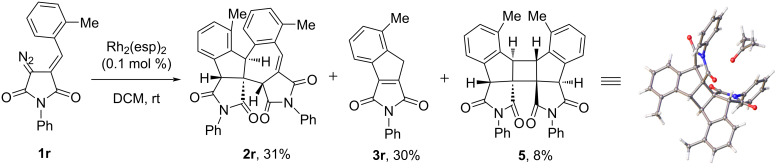
The result of Rh(II)-catalyzed decomposition of DAS **1r**.

Rather remarkable was the reaction of DAS **1r** containing an *ortho*-methyl substituent in the benzylidene fragment. In this case, along with conventional reaction products ‒ dimer **2r** and indene **3r**, unexpectedly the cyclobutane **5**, a product of the formal [2 + 2] cycloaddition, was isolated in low yield (Scheme 1); its structure was confirmed by single-crystal X-ray analysis (see Supporting Information File 1).

**Scheme 2:**
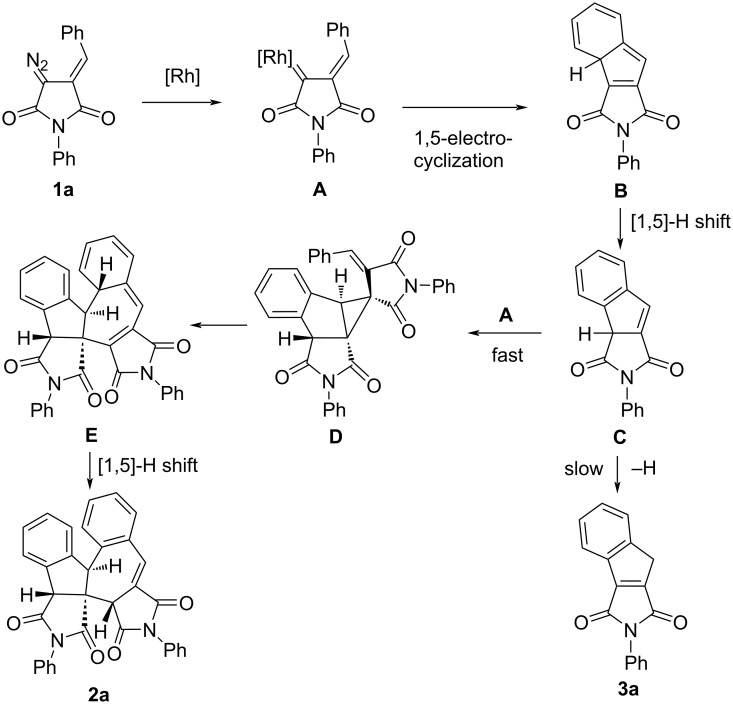
Plausible mechanism for the formation of dimer **2a** and indene **3a** through the Rh(II)-catalyzed decomposition of **1a**.

A plausible mechanism of the observed transformations of DAS (shown for **1a**) is presented in Scheme 2. The initially formed rhodium carbene **A** undergoes 1,5-electrocyclization to form intermediate **B** which turns into indene **C** as the result of a 1,5-suprafacial hydrogen shift. Indene **C** can either convert into the final indene **3a** via a slow 1,3-migration of the hydrogen atom, or is intercepted by carbene **A** with the formation of cyclopropane **D**. The relative rates of these competing processes likely determine the composition of the final product mixture. The formation of a single diastereomer at the cyclopropanation step can be explained by the preferred approach of carbene **A** from the least sterically hindered side of indene **C** and the π-stacking interaction of the aromatic fragment of indene and the benzylidene substituent of the carbene.

The conversion of intermediate **D** into cycloheptadiene **E** is an example of a relatively rare reaction of the cyclopropane-ring expansion through a 1,5-C‒C bond migration [15–17]. This concerted process is followed by yet another 1,5-migration of the hydrogen atom, leading to the final dimer **2a**. The formation of a single diastereomer **2a** is indicative of a sequence of concerted processes with an unambiguous stereochemistry control at each step where stereogenic centers are formed.

**Figure 2:**
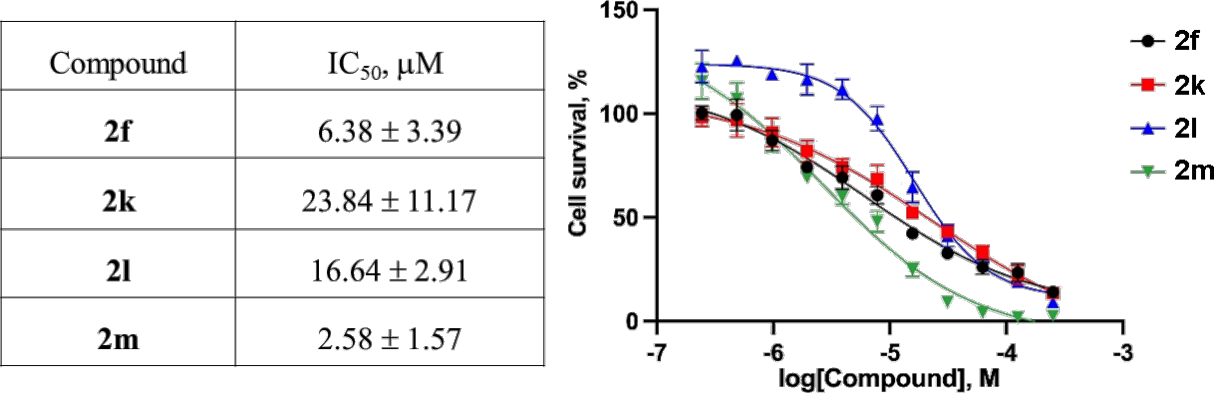
Cytotoxicity of *N*-alkyl-substituted dibenzoazulenodipyrroles **2** against the A549 human lung adenocarcinoma cell line (the IC_50_ values are mean values from three different assays).

Dibenzoazulenodipyrroles **2** have a pronounced three-dimensional character which make this chemotype promising as probe for protein–protein interactions, including oncogenic ones [18]. As the first step towards biological characterization of compounds **2**, they were screened for cytotoxicity against the A549 human lung adenocarcinoma cell line. Among the eleven compounds, *N*-aryl analogs **2a–e**, **2h**, and **2j** had no effect on the cancer cell viability. However, the *N*-alkyl analogs **2f** and **2k–m** showed a pronounced cytotoxicity with IC_50_ values in the single to double-digit micromolar range (Figure 2).

## Conclusion

In summary, we have shown that the reaction triggered by the Rh(II)-catalyzed decomposition of DAS in inert medium can proceed in two principal directions and result in the formation of an unusual unsymmetrical dibenzoazulenodipyrrole dimer **2** and/or a product of intramolecular cyclization ‒ indene **3**. The result of the reaction is determined by the nature of the substituents in the starting DAS molecule. In most cases, dimers **2** were obtained as a single diastereomer in high yields. In some cases, indenes were obtained in moderate yields. A plausible mechanism of the observed transformations was proposed which implicates a rare rearrangement of the cyclopropane intermediate as the key step. Dimers **2** bearing alkyl substituents at the nitrogen atom showed pronounced cytotoxocity against the A549 human lung adenocarcinoma cell line while *N*-aryl analogs were non-cytotoxic.

## Supporting Information

File 1 General experimental information, X-ray crystallographic data, synthetic procedures, analytical data and NMR spectra for the reported compounds.
