# Supplementary material for: Unusual highly diastereoselective Rh(II)-catalyzed dimerization of 3-diazo-2-arylidenesuccinimides provides access to a new dibenzazulene scaffold
Source: Beilstein J Org Chem. 2022 May 11;18:533–8. doi: 10.3762/bjoc.18.55 (PMC9112184; doi:10.3762/bjoc.18.55)

BEILSTEIN JOURNAL OF ORGANIC CHEMISTRY

Supporting Information

for

Unusual highly diastereoselective Rh(II)-catalyzed dimerization of 3-diazo-2-arylidenesuccinimides provides access to a new dibenzazulene scaffold

Anastasia Vepreva, Alexander S. Bunev, Andrey Yu. Kudinov, Grigory Kantin, Mikhail Krasavin and Dmitry Dar'in

Beilstein J. Org. Chem. **2022,** *18,* 533–538. doi:10.3762/bjoc.18.55

General experimental information, X-ray crystallographic data, synthetic procedures, analytical data and NMR spectra for the reported compounds

License and Terms: This is a supporting information file under the terms of the Creative Commons Attribution License (https://creativecommons.org/licenses/by/4.0). Please note that the reuse, redistribution and reproduction in particular requires that the author(s) and source are credited and that individual graphics may be subject to special legal provisions.
The license is subject to the *Beilstein Journal of Organic Chemistry* terms and conditions: (https://www.beilstein-journals.org/bjoc/terms)

Table of contents

1 Experimental procedures and characterization data S2

2 Crystallographic data for compounds **2a** and **5** S10

3 Biological data S12

4 References S13

5 Copies of ^{1}H, ^{13}C, ^{19}F NMR and NOESY NMR spectra S14

1. Experimental procedure and characterization data

General information. All commercial reagents were used without purification. NMR spectra were recorded using a Bruker Avance III spectrometer in $CDCl_3$ (^1H: 400.13 MHz; ^{13}C: 100.61 MHz; ^{19}F: 376.50 MHz); chemical shifts are reported as parts per million (δ, ppm). The residual solvent peak ($CHCl_3$) was used as internal standard: 7.26 for ^1H and 77.16 ppm for ^{13}C. Multiplicities are abbreviated as follows: s = singlet, d = doublet, t = triplet, q = quartet, m = multiplet, br = broad, dd = doublet of doublets, dt = doublet of triplets, ddd = doublet/doublets of doublets. Coupling constants, *J*, are reported in Hz. Mass spectra were recorded using a Bruker microTOF spectrometer (ionization by electrospray, positive ions detection). Melting points were determined in open capillary tubes on a Stuart SMP50 Automatic Melting Point Apparatus. Analytical thin-layer chromatography was carried out on UV-254 silica gel plates using appropriate eluents. Compounds were visualized with short-wavelength UV light. Column chromatography was performed with a flash purification system Isolera™ Prime (Biotage®) using silica gel Merk grade 60 (0.040–0.063 mm) 230–400 mesh (gradient elution with *n*-hexane/acetone). HPLC was performed using an ECS28P00 instrument and YMC-Pack SIL-06 (250 × 20 mm) column. Diazo compounds **1** were synthesized according to previously described procedures.[1,2]

General procedure for the catalytic decomposition of DAS 1 in DCM. Diazo compound **1** (0.5 or 1.0 mmol) was dissolved in dry DCM (1.8 or 3.6 mL) followed by addition of the catalyst solution (2.5 mM $Rh_2(esp)_2$ in DCM, 200 or 400 μL, 0.1 mol %). The reaction mixture was stirred at ambient temperature for 0.5–1 h (controlled by TLC). The reaction mixture was diluted with *n*-hexane (2 mL) and the resulting solution was subjected to column chromatography on silica gel, eluent cyclohexane/acetone (gradient from 10 to 50% of acetone) to afford dimer **2** and/or indene **3**. In some cases, additional purification by HPLC was performed.

(8b*R/S*,12b*R/S*,15a*S/R*,15b*S/R*)-2,14-Diphenyl-14,15b-dihydro-1*H*-dibenzo[2,3:4,5]azuleno[1,8*a*-*c*:7,8-*c*']dipyrrole-1,3,13,15(2*H*,12b*H*,14*H*)-tetraone (2a): prepared according to the general procedure from diazo compound **1a** (0.5 mmol). Yield: 97 mg (74%). Eluent – cyclohexane–acetone (from 0 to 25% of acetone). White solid; m.p. >250 °C. ^1H NMR (400 MHz, $CDCl_3$) δ 7.98 (d, *J* = 1.8 Hz, 1H), 7.74 – 7.70 (m, 1H), 7.51 – 7.46 (m, 2H), 7.45 – 7.40 (m, 4H), 7.38 (m, 1H), 7.34 – 7.30 (m, 4H), 7.27 – 7.24 (m, 1H), 7.20 – 7.16 (m, 2H), 6.98 (m, 2H), 6.76 (m, 1H), 5.31 (s, 1H), 4.76 (s, 1H), 3.71 (d, *J* = 1.8 Hz, 1H). ^{13}C NMR (101 MHz, $CDCl_3$) δ 176.7, 173.9, 172.9, 166.9, 139.8, 139.3, 139.0, 136.4, 135.5, 131.4, 131.2, 130.5, 130.0, 129.4, 129.2, 129.12, 129.11, 128.9, 128.9, 128.7, 127.9, 127.7, 126.6, 126.4, 125.5, 72.3, 55.4, 53.0, 46.8. HRMS (ESI), *m/z* calcd for $C_{34}H_{22}N_2NaO_4$ $[M+Na]^+$ 545.1472 found 545.1478.

Additionally, the experiment with diluting the reaction mixture 10 times (0.5 mmol in 18 mL) was performed. In this case, the content of indene increased, and it was isolated using column chromatography.

2-Phenylindeno[1,2-*c*]pyrrole-1,3(2*H*,8*H*)-dione (3a): yield: 19 mg (15%). Eluent – cyclohexane–acetone (from 10 to 50% of acetone). White solid; 163.2 – 165.1 °C. ^{1}H NMR (400 MHz, $CDCl_3$) δ 7.96 – 7.83 (m, 1H), 7.70 – 7.58 (m, 1H), 7.55 – 7.46 (m, 4H), 7.41 (m, 3H), 3.87 (s, 2H). ^{13}C NMR (101 MHz, $CDCl_3$) δ 165.6, 164.5, 151.3, 148.9, 148.2, 134.4, 132.1, 129.1, 129.0, 127.9, 127.7, 126.6, 125.5, 123.0, 33.6. HRMS (ESI), *m/z* calcd for $C_{17}H_{12}NO_2$ $[M+H]^+$ 262.0863 found 262.0865.

(8b*R/S*,12b*R/S*,15a*S/R*,15b*S/R*)-2,14-Bis(4-methoxyphenyl)-14,15b-dihydro-1*H*-dibenzo[2,3:4,5]-azuleno[1,8*a*-*c*:7,8-*c'*]dipyrrole-1,3,13,15(2*H*,12b*H*,14*H*)-tetraone (2b): prepared according to the general procedure from diazo compound **1b** (0.5 mmol). Yield: 106 mg (73%). Eluent – cyclohexane–acetone (from 10 to 50% of acetone). White solid, m.p. 195.7 – 196.9 °C. ^{1}H NMR (400 MHz, $CDCl_3$) δ 7.95 (d, *J* = 2.0 Hz, 1H), 7.72 – 7.69 (m, 1H), 7.51 – 7.42 (m, 3H), 7.38 (m, 1H), 7.33 – 7.29 (m, 1H), 7.25 (m 1H), 7.11 – 7.07 (m, 2H), 6.96 – 6.92 (m, 2H), 6.91 – 6.87 (m, 2H), 6.84 – 6.80 (m, 2H), 6.75 (m, 1H), 5.29 (s, 1H), 4.73 (s, 1H), 3.85 (s, 3H), 3.80 (s, 3H), 3.67 (d, *J* = 2.0 Hz, 1H). ^{13}C NMR (101 MHz, $CDCl_3$) δ 176.9, 174.2, 173.2, 167.2, 159.8, 159.6, 139.9, 139.3, 139.0, 136.4, 135.2, 130.4, 130.1, 129.42, 129.37, 129.2, 129.1, 127.88, 127.85, 127.67, 127.61, 125.5, 124.0, 123.9, 114.4, 114.2, 72.1, 55.52, 55.48, 55.3, 52.9, 46.7. HRMS (ESI), *m/z* calcd for $C_{36}H_{26}N_2NaO_6$ $[M+Na]^+$ 605.1683 found 605.1691.

(8b*R/S*,12b*R/S*,15a*S/R*,15b*S/R*)-2,14-Bis(4-(trifluoromethyl)phenyl)-14,15b-dihydro-1*H*-dibenzo-[2,3:4,5]azuleno[1,8*a*-*c*:7,8-*c'*]dipyrrole-1,3,13,15(2*H*,12b*H*,14*H*)-tetraone (2c): prepared according to the general procedure from diazo compound **1c** (0.5 mmol). Yield: 121 mg (74%). Eluent – cyclohexane–acetone (from 10 to 50% of acetone). White solid; m. p. 198.2 – 199.5 °C. ^{1}H NMR (400 MHz, $CDCl_3$) δ 8.02 (d, *J* = 2.0 Hz, 1H), 7.74 – 7.64 (m, 3H), 7.57 (d, *J* = 8.4 Hz, 2H), 7.53 – 7.44 (m, 3H), 7.41 (t, *J* = 7.6 Hz, 1H), 7.33 (m, 4H), 7.16 (d, *J* = 8.4 Hz, 2H), 6.76 (d, *J* = 7.6 Hz, 1H), 5.31 (s, 1H), 4.79 (s, 1H), 3.76 (d, *J* = 2.0 Hz, 1H). ^{13}C NMR (101 MHz, $CDCl_3$) δ 176.3, 173.3, 172.7, 166.2, 139.6, 138.8, 138.7, 136.5, 136.1, 134.4 (q, *J* = 1.2 Hz), 134.2 (q, *J* = 1.1 Hz), 131.0 (q, *J* = 14.6 Hz), 130.7 (q, *J* = 14.6 Hz), 130.6, 129.8, 129.5, 129.44, 129.4, 129.2, 127.9, 127.8, 126.6, 126.46 (s), 126.2 (q, *J* = 3.7 Hz), 126.0 (q, *J* = 3.7 Hz), 125.4, 124.8 (q, *J* = 272.7 Hz), 122.1 (q, *J* = 273.7 Hz), 72.4, 55.3, 52.9, 46.9. ^{19}F NMR (376 MHz, $CDCl_3$) δ -62.85, -62.88. HRMS (ESI), *m/z* calcd for $C_{36}H_{20}F_6N_2NaO_4$ $[M+Na]^+$ 681.1219 found 681.1225.

(8b*R/S*,12b*R/S*,15a*S/R*,15b*S/R*)-2,14-Di-*p*-tolyl-14,15b-dihydro-1*H*-dibenzo[2,3:4,5]azuleno[1,8*a*-*c*:7,8-*c'*]dipyrrole-1,3,13,15(2*H*,12b*H*,14*H*)-tetraone (2d): prepared according to the general procedure from diazo compound **1d** (1 mmol). Yield: 173 mg (63%). Eluent – cyclohexane–acetone (from 10 to 50% of acetone). White solid; m.p. 177.2 – 178.4 °C. ^{1}H NMR (400 MHz, $CDCl_3$) δ 7.96 (d, *J* = 2.0 Hz, 1H), 7.73 – 7.67 (m, 1H), 7.49 – 7.42 (m, 3H), 7.38 (t, *J* = 7.6 Hz, 1H), 7.33 – 7.29 (m, 1H), 7.25 (m, 3H), 7.12 (m, 2H), 7.06 (d, *J* = 8.3 Hz, 2H), 6.86 (d, *J* = 8.3 Hz, 2H), 6.75 (d, *J* = 7.6 Hz, 1H), 5.29 (s, 1H), 4.74 (s, 1H), 3.68 (d, *J* = 2.0 Hz, 1H), 2.41 (s, 3H), 2.35 (s, 3H). ^{13}C NMR (101 MHz, $CDCl_3$) δ 176.8, 174.1, 173.0, 167.0, 139.9, 139.4, 139.1, 139.0, 138.8, 136.4, 135.3, 130.4, 130.2, 129.8, 129.6, 129.43, 129.37, 129.2, 129.1, 128.8, 128.7, 127.9, 127.6, 126.4, 126.2, 125.5, 72.2, 55.4, 53.0, 46.8, 21.3, 21.2. HRMS (ESI), *m/z* calcd for $C_{36}H_{27}N_2O_4$ $[M+H]^+$ 551.1965 found 551.1969.

(8b*R/S*,12b*R/S*,15a*S/R*,15b*S/R*)-2,14-Bis(4-fluorophenyl)-14,15b-dihydro-1*H*-dibenzo[2,3:4,5]-azuleno[1,8*a*-*c*:7,8-*c'*]dipyrrole-1,3,13,15(2*H*,12b*H*,14*H*)-tetraone (2e): prepared according to the general procedure from diazo compound **1e** (1 mmol)**.** Yield: 211 mg (86%). Eluent – cyclohexane–acetone (from 10 to 50% of acetone). White solid, m.p. 160.1 – 160.9 °C. ^{1}H NMR (400 MHz, $CDCl_3$) δ 7.98 (d, *J* = 2.0 Hz, 1H), 7.72 – 7.68 (m, 1H), 7.50 – 7.43 (m, 3H), 7.39 (t, *J* = 7.6 Hz, 1H), 7.34 – 7.30 (m, 1H), 7.27 – 7.23 (m, 1H), 7.14 – 7.11 (m, 4H), 6.97 (m, 4H), 6.75 (d, *J* = 7.6 Hz, 1H), 5.29 (s, 1H), 4.75 (s, 1H), 3.71 (d, *J* = 2.0 Hz, 1H). ^{13}C NMR (101 MHz, $CDCl_3$) δ 176.7, 173.8, 173.0, 166.7, 163.6 (d, *J* = 250.5 Hz), 161.2 (d, *J* = 250.5 Hz), 139.7, 139.0, 138.9, 136.2, 135.9, 130.5, 129.7, 129.6, 129.5, 129.3 (d, *J* = 6.2 Hz), 128.5, 128.4, 128.3 (d, *J* = 8.8 Hz), 127.9, 127.7, 127.2 (d, *J* = 3.3 Hz), 127.1 (d, *J* = 3.2 Hz), 125.5, 116.33 (s), 116.2 (d, *J* = 22.8 Hz), 115.9 (d, *J* = 250.5 Hz), 72.3, 55.3, 52.9, 46.8. ^{19}F NMR (376 MHz, $CDCl_3$) δ -111.5, -111.8. HRMS (ESI), *m/z* calcd for $C_{34}H_{20}F_2N_2NaO_4$ $[M+Na]^+$ 581.1283 found 581.1292.

(8b*R/S*,12b*R/S*,15a*S/R*,15b*S/R*)-2,14-Dibenzyl-14,15b-dihydro-1*H*-dibenzo[2,3:4,5]azuleno[1,8*a*-*c*:7,8-*c'*]dipyrrole-1,3,13,15(2*H*,12b*H*,14*H*)-tetraone (2f): prepared according to the general procedure from diazo compound **1f** (1 mmol). Yield: 187 mg (68%). White solid, m.p. 232.0–234.0 °C. Eluent – cyclohexane–acetone (from 10 to 50% of acetone). ^{1}H NMR (400 MHz, $CDCl_3$) δ 7.81 (d, *J* = 1.9 Hz, 1H), 7.67 – 7.63 (m, 1H), 7.43 – 7.38 (m, 2H), 7.32 (m, 7H), 7.21 (m, 5H), 7.03 (m, 2H), 6.70 (m, 1H), 5.10 (s, 1H), 4.59 (s, 1H), 4.28 (dd, *J* = 49.6, 14.3 Hz, 2H), 4.06 (dd, *J* = 14.1, 12.2 Hz, 2H), 3.36 (d, *J* = 1.9 Hz, 1H). NMR data is in accordance with previously reported.[3]

(8b*S/R*,12b*R/S*,15a*S/R*,15b*S/R*)-7,11-Dimethyl-2,14-diphenyl-14,15b-dihydro-1*H*-dibenzo-[2,3:4,5]azuleno[1,8*a-c*:7,8-*c'*]dipyrrole-1,3,13,15(2*H*,12b*H*,14*H*)-tetraone (2h): prepared according to the general procedure from diazo compound **1h** (1.0 mmol). Yield: 253 mg (93%). Eluent – cyclohexane–acetone (from 10 to 50% of acetone). White solid; m.p. >250 °C. ^{1}H NMR (400 MHz, $CDCl_3$) δ 7.93 (d, *J* = 2.0 Hz, 1H), 7.52 (s, 1H), 7.44 – 7.38 (m, 3H), 7.34 – 7.30 (m, 4H), 7.29 (m, 1H), 7.20 – 7.14 (m, 4H), 7.03 – 6.97 (m, 2H), 6.59 (s, 1H), 5.23 (s, 1H), 4.71 (s, 1H), 3.68 (d, *J* = 2.0 Hz, 1H), 2.46 (s, 3H), 2.28 (s, 3H). ^{13}C NMR (101 MHz, $CDCl_3$) δ 176.8, 174.2, 173.1, 167.0, 139.9, 139.24, 139.23, 139.21, 136.8, 135.8, 133.6, 131.5, 131.3, 130.5, 130.4, 130.1, 129.09, 129.06, 128.88, 128.80, 128.6, 128.2, 127.6, 126.6, 126.4, 125.8, 72.4, 55.3, 52.6, 46.9, 21.7, 21.4. HRMS (ESI), *m/z* calcd for $C_{36}H_{27}N_2O_4$ $[M+H]^+$ 551.1965 found 551.1968.

(8b*S/R*,12b*R/S*,15a*S/R*,15b*S/R*)-2,14-Diphenyl-7,11-bis(trifluoromethyl)-14,15b-dihydro-1*H*-dibenzo[2,3:4,5]azuleno[1,8*a-c*:7,8-*c'*]dipyrrole-1,3,13,15(2*H*,12b*H*,14*H*)-tetraone (2i): prepared according to the general procedure from diazo compound **1i** (0.5 mmol). Eluent – cyclohexane–acetone (from 10 to 50% of acetone) and additionally purified by HPLC (eluent – *n*-hexane – ethyl acetate, from 10 to 30% of ethyl acetate). Yield: 30 mg (18%). Amorphous solid. ^{1}H NMR (400 MHz, $CDCl_3$) δ 8.03 (s, 1H), 7.99 (d, *J* = 2.1 Hz, 1H), 7.81 (d, *J* = 8.0 Hz, 1H), 7.69 (d, *J* = 7.9 Hz, 1H), 7.61 (d, *J* = 7.9 Hz, 1H), 7.47 (d, *J* = 8.0 Hz, 1H), 7.44 – 7.41 (m, 3H), 7.40 – 7.31 (m, 4H), 7.18 – 7.12 (m, 2H), 7.01 – 6.95 (m, 3H), 5.39 (s, 1H), 4.87 (s, 1H), 3.73 (d, *J* = 2.1 Hz, 1H). ^{13}C NMR (101 MHz, $CDCl_3$) δ 175.9, 172.9, 172.2, 166.2, 142.8, 140.1, 139.9, 138.9, 133.6, 132.4 (q, *J* = 33.0 Hz), 131.8, 131.3 (q, *J* = 32.9 Hz), 131.1, 130.8, 129.22, 129.17, 129.1, 128.3, 126.70 (q, *J* = 3.3 Hz), 126.4, 126.2, 125.8 (q, *J* = 3.6 Hz), 125.0 (q, *J* = 3.5 Hz), 124.5 (q, *J* = 273.4 Hz), 123.0 (q, *J* = 3.6 Hz), 122.4 (q, *J* = 273.4 Hz), 72.2, 55.0, 52.7, 46.4. ^{19}F NMR (376 MHz, $CDCl_3$) δ -62.4, -62.9. HRMS (ESI), *m/z* calcd for $C_{36}H_{20}F_6N_2NaO_4$ $[M+Na]^+$ 681.1220 found 681.1218.

(8bS/R,12bR/S,15aS/R,15bS/R)-5,9-Difluoro-2,14-diphenyl-14,15b-dihydro-1*H*-dibenzo[2,3:4,5]-azuleno[1,8*a-c*:7,8-*c'*]dipyrrole-1,3,13,15(2*H*,12b*H*,14*H*)-tetraone (2j): prepared according to the general procedure from diazo compound **1j** (1.0 mmol). Yield: 167 mg (60%). Eluent – cyclohexane–acetone (from 10 to 50% of acetone). White solid, m.p. >250 °C. ^{1}H NMR (400 MHz, $CDCl_3$) δ 8.14 (t, *J* = 2.0 Hz, 1H), 7.54 – 7.47 (m, 2H), 7.44 – 7.40 (m, 3H), 7.36 – 7.29 (m, 4H), 7.21 – 7.12 (m, 4H), 6.98 (m, 2H), 6.58 (m, 1H), 5.40 (s, 1H), 4.80 (s, 1H), 3.76 (d, *J* = 2.0 Hz, 1H). ^{13}C NMR (101 MHz, $CDCl_3$) δ 176.0, 173.2, 172.5, 166.2, 161.3 (d, *J* = 165.0 Hz), 159.3 (d, *J* = 164.7 Hz), 141.7 (d, *J* = 4.8 Hz), 138.3, 131.9 (d, *J* = 7.3 Hz), 131.2, 131.0, 130.97, 130.89, 129.2, 129.03, 129.01, 128.9, 128.8 (d, *J* =

6.2 Hz), 126.7 (d, J = 17.7 Hz), 126.5, 126.3, 124.3 (d, J = 2.8 Hz), 123.8 (d, J = 14.4 Hz), 121.4 (d, J = 3.7 Hz), 116.2 (d, J = 19.7 Hz), 115.4 (d, J = 21.5 Hz), 72.2, 55.4, 50.0, 46.5. ^{19}F NMR (376 MHz, $CDCl_3$) δ -112.0, -113.2. HRMS (ESI), *m/z* calcd for $C_{34}H_{20}F_2N_2NaO_4$ $[M+Na]^+$ 581.1283 found 581.1280.

(8b*S/R*,12b*R/S*,15a*S/R*,15b*S/R*)-2,14-Dibenzyl-7,11-difluoro-14,15b-dihydro-1*H*-dibenzo[2,3:4,5]-azuleno[1,8*a*-*c*:7,8-*c'*]dipyrrole-1,3,13,15(2*H*,12b*H*,14*H*)-tetraone (2k) and (3'*E*,4*Z*,4'*Z*)-4,4'-(hydrazine-1,2-diylidene)bis(1-benzyl-3-((*E*)-4-fluorobenzylidene)-pyrrolidine-2,5-dione) (4k): prepared according to the general procedure from diazo compound **1g** (1.0 mmol). As a result of the reaction, the bisazine **4k** precipitate was obtained, which was additionally purified by recrystallization from hot methanol. The solution with dimer **2k** and other soluble impurities was subjected to column chromatography on silica gel, eluent cyclohexane–acetone (gradient from 10 to 50% of acetone).

Compound 2k: Yield: 123 mg (42%) White solid, m.p. 237.1 – 239.0 °C. ^{1}H NMR (400 MHz, $CDCl_3$) δ 7.76 (d, J = 2.0 Hz, 1H), 7.37 – 7.33 (m, 3H), 7.32 – 7.30 (m, 4H), 7.27 – 7.21 (m, 4H), 7.17 (m, 2H), 7.08 (m, 2H), 7.03 (m, 1H), 6.43 (m, 1H), 5.02 (s, 1H), 4.57 (s, 1H), 4.32 (d, J = 14.1 Hz, 1H), 4.22 (d, J = 14.3 Hz, 1H), 4.08 (d, J = 14.3 Hz, 1H), 4.02 (d, J = 14.1 Hz, 1H), 3.34 (d, J = 2.0 Hz, 1H). ^{13}C NMR (101 MHz, $CDCl_3$) δ 176.4, 174.1, 172.3, 167.3, 164.2 (d, J = 250.4 Hz), 161.7 (d, J = 253.5 Hz), 141.7 (d, J = 6.2 Hz), 141.3 (d, J = 8.8 Hz), 135.5, 134.9, 134.6 (d, J = 2.8 Hz), 133.4, 132.5, 132.4 (d, J = 2.5 Hz), 132.3, 130.0 (d, J = 0.7 Hz), 129.0, 128.9, 128.6 (d, J = 3.5 Hz), 128.5, 128.0, 127.9, 117.0 (d, J = 13.4 Hz), 116.8 (d, J = 12.0 Hz), 114.6 (d, J = 21.8 Hz), 112.7 (d, J = 23.3 Hz), 71.5, 55.4 (d, J = 1.9 Hz), 52.0, 46.0, 42.2, 42.0. ^{19}F NMR (376 MHz, $CDCl_3$) δ -108.5, -111.2. HRMS (ESI), *m/z* calcd for $C_{36}H_{24}F_2N_2NaO_4$ $[M+Na]^+$ 609.1596 found 609.1600.

Compound 4k: Yield: 56 mg (18%). Yellow solid, m.p. 245.2 – 247.5 °C. ^{1}H NMR (400 MHz, $CDCl_3$) δ 8.38 (m, 4H), 7.93 (s, 2H), 7.43 (m, 4H), 7.35 (m, 6H), 7.09 (m, 4H), 4.83 (s, 4H). ^{13}C NMR (101 MHz, $CDCl_3$) δ 167.9, 165.0 (d, J = 256.3 Hz), 160.8, 141.0, 136.0 (d, J = 9.0 Hz), 135.2, 134.0, 129.5 (d, J = 3.0 Hz), 129.0, 128.8, 128.3, 118.5 (d, J = 2.2 Hz), 116.0 (d, J = 21.8 Hz), 42.6. ^{19}F NMR (376 MHz, $CDCl_3$) δ -105.8. HRMS (ESI), *m/z* calcd for $C_{36}H_{24}F_2N_2NaO_4$ $[M+Na]^+$ 637.1658 found 637.1661.

(8b*S/R*,12b*R/S*,15a*S/R*,15b*S/R*)-2,14-Dicyclopropyl-7,11-difluoro-14,15b-dihydro-1*H*-dibenzo-[2,3:4,5]azuleno[1,8*a*-*c*:7,8-*c'*]dipyrrole-1,3,13,15(2*H*,12b*H*,14*H*)-tetraone (2l): prepared according to the general procedure from diazo compound **1l** (0.86 mmol). Yield: 89 mg (43%). Eluent – cyclohexane–acetone (from 10 to 50% of acetone). White solid; m.p. >250 °C. ^{1}H NMR (400 MHz, $CDCl_3$) δ 7.75 (d, J = 2.0 Hz, 1H), 7.37 (m, 2H), 7.17 (m, 2H), 7.07 – 6.99 (m, 1H), 6.40 (m, 1H), 5.02

(s, 1H), 4.46 (s, 1H), 3.30 (d, J = 2.0 Hz, 1H), 2.75 – 2.69 (m, 1H), 2.47 (m, 1H), 1.10 – 1.04 (m, 1H), 1.00 (m, 3H), 0.95 – 0.84 (m, 3H), 0.69 (m, 1H). ^{13}C NMR (101 MHz, $CDCl_3$) δ 177.9, 175.3, 174.3, 168.5, 163.47 (d, J = 246.9 Hz), 161.5 (d, J = 248.2 Hz), 142.4 (d, J = 8.7 Hz), 135.5, 133.3, 133.2 (d, J = 3.1 Hz), 132.6, 130.6, 130.0 (d, J = 9.0 Hz), 116.9, 116.7 (d, J = 6.1 Hz), 116.5, 114.6 (d, J = 21.4 Hz), 112.1 (d, J = 22.9 Hz), 71.2, 54.6, 51.9, 45.5, 22.4, 22.2, 5.2, 5.0, 4.9, 4.78. ^{19}F NMR (376 MHz, $CDCl_3$) δ -110.3, -112.3. HRMS (ESI), *m/z* calcd for $C_{28}H_{20}F_2N_2NaO_4$ $[M+Na]^+$ 509.1283 found 509.1294.

(8b*S/R*,12b*R/S*,15a*S/R*,15b*S/R*)-7,11-Dichloro-2,14-diisobutyl-14,15b-dihydro-1*H*-dibenzo-[2,3:4,5]azuleno[1,8*a*-*c*:7,8-*c'*]dipyrrole-1,3,13,15(2*H*,12b*H*,14*H*)-tetraone (2m): prepared according to the general procedure from diazo compound **1m** (1.0 mmol). Yield: 143 mg (52%). Eluent – cyclohexane–acetone (from 10 to 50% of acetone). White solid; m.p. 198.9 – 200.0 °C. ^{1}H NMR (400 MHz, $CDCl_3$) δ 7.76 (d, J = 2.0 Hz, 1H), 7.68 (s, 1H), 7.44 (m, 1H), 7.34 (m, 2H), 7.18 (m, 1H), 6.66 (s, 1H), 5.07 (s, 1H), 4.55 (s, 1H), 3.41 (d, J = 2.0 Hz, 1H), 3.39 – 3.31 (m, 2H), 3.23 (m, 1H), 3.09 (m, 1H), 1.99 (m, 1H), 1.77 (m, 1H), 0.91 (d, J = 2.3 Hz, 3H), 0.89 (d, J = 2.3 Hz, 3H), 0.62 (d, J = 6.7 Hz, 3H), 0.57 (d, J = 6.7 Hz, 3H). ^{13}C NMR (101 MHz, $CDCl_3$) δ 177.2, 174.7, 173.4, 167.8, 141.6, 140.5, 137.4, 135.4, 135.4, 134.9, 132.9, 131.7, 130.6, 129.7, 129.5, 128.6, 127.9, 125.8, 71.2, 55.3, 52.5, 46.3, 46.0, 45.9, 27.3, 26.8, 20.1, 19.9, 19.6, 19.5. HRMS (ESI), *m/z* calcd for $C_{30}H_{29}Cl_2N_2O_4$ $[M+H]^+$ 551.1499 found 551.1497.

(8b*R/S*,12b*R/S*,15a*S/R*,15b*S/R*)-6,10-Dimethoxy-2,14-diphenyl-14,15b-dihydro-1*H*-dibenzo-[2,3:4,5]azuleno[1,8*a*-*c*:7,8-*c'*]dipyrrole-1,3,13,15(2*H*,12b*H*,14*H*)-tetraone (2n): prepared according to the general procedure from diazo compound **1n** (0.5 mmol). After column chromatography (eluent – cyclohexane–acetone (from 10% to 50% of acetone) two fractions were obtained: a mixture of two regioisomeric indenes (ratio 1.8:1.0, yield 82 mg (56%)) and contaminated dimer **2n**. Pure compound **2n** was obtained by HPLC (eluent – DCM–methanol (from 0% to 10% of methanol). Yield: 18 mg (12%). Amorphous solid. ^{1}H NMR (400 MHz, $CDCl_3$) δ 7.91 (d, J = 2.0 Hz, 1H), 7.59 (m, 1H), 7.42 (m, 3H), 7.31 (m, 3H), 7.19 – 7.14 (m, 2H), 7.02 – 6.96 (m, 4H), 6.80 (m, 2H), 6.74 (m, 1H), 5.17 (s, 1H), 4.65 (s, 1H), 3.84 (s, 6H), 3.70 (d, J = 2.0 Hz, 1H). ^{13}C NMR (101 MHz, $CDCl_3$) δ 176.9, 174.3, 173.0, 166.9, 160.6, 158.9, 141.7, 137.4, 135.4, 131.4, 131.3, 131.2, 130.74, 130.68, 130.5, 129.1, 128.93, 128.90, 128.7, 126.6, 126.4, 126.2, 115.9, 115.7, 114.5, 112.4, 72.4, 55.6, 55.5, 54.6, 52.5, 46.9. HRMS (ESI), *m/z* calcd for $C_{36}H_{26}N_2NaO_6$ $[M+Na]^+$ 605.1683 found 605.1680.

2-(2-Chlorophenyl)indeno[1,2-*c*]pyrrole-1,3(2*H*,8*H*)-dione (3o): prepared according to the general procedure from diazo compound **1o** (1.0 mmol). Yield: 105 mg (35%). Eluent – cyclohexane–acetone

(from 10 to 50% of acetone). White solid; m.p. 197.7 – 199.1 °C. ^{1}H NMR (400 MHz, $CDCl_3$) δ 7.92 (m, 1H), 7.70 – 7.65 (m, 1H), 7.58 (m, 1H), 7.53 – 7.46 (m, 2H), 7.46 – 7.40 (m, 2H), 7.41 – 7.34 (m, 1H), 3.93 (d, *J* = 24.6 Hz, 1H), 3.85 (d, *J* = 24.6 Hz, 1H). ^{13}C NMR (101 MHz, $CDCl_3$) δ 165.1, 163.9, 151.7, 149.2, 148.2, 134.4, 133.7, 131.1, 130.5, 130.4, 129.9, 129.1, 127.9, 127.7, 125.5, 123.1, 33.7. HRMS (ESI), *m/z* calcd for $C_{17}H_{10}ClNNaO_2$ $[M+Na]^+$ 318.0292 found 318.0288.

2-(5-Chloro-2-methoxyphenyl)indeno[1,2-*c*]pyrrole-1,3(2*H*,8*H*)-dione (3p): prepared according to the general procedure from diazo compound **1p** (1.0 mmol). Yield: 111 mg (34%). Eluent – cyclohexane–acetone (from 10 to 50% of acetone). White solid; m.p. 184.5 – 185.7 °C. ^{1}H NMR (400 MHz, $CDCl_3$) δ 7.90 (m, 1H), 7.68 – 7.64 (m, 1H), 7.48 (m, 2H), 7.40 (m, 1H), 7.28 (s, 1H), 6.98 (m, 1H), 3.90 (d, *J* = 24.1 Hz, 1H), 3.83 (d, *J* = 24.1 Hz, 1H), 3.82 (s, 3H). ^{13}C NMR (101 MHz, $CDCl_3$) δ 165.4, 164.3, 154.6, 151.7, 149.3, 148.2, 134.5, 130.5, 130.3, 128.9, 127.8, 125.5, 125.4, 123.0, 121.6, 113.0, 56.2, 33.6. HRMS (ESI), *m/z* calcd for $C_{18}H_{12}ClNNaO_3$ $[M+Na]^+$ 348.0398 found 348.0400.

Ethyl 2-(1,3-dioxoindeno[1,2-*c*]pyrrol-2(1*H*,3*H*,8*H*)-yl)benzoate (3q): prepared according to the general procedure from diazo compound **1q** (1.0 mmol). Yield: 180 mg (54%). Eluent – cyclohexane–acetone (from 10 to 50% of acetone). White solid; m.p. 158.6 – 160.9 °C. ^{1}H NMR (400 MHz, $CDCl_3$) δ 8.17 (m, 1H), 7.93 – 7.88 (m, 1H), 7.68 (m, 2H), 7.55 (m, 1H), 7.51 – 7.45 (m, 2H), 7.40 (m, 1H), 4.26 (q, *J* = 7.1 Hz, 2H), 3.89 (br.s, 2H), 1.24 (t, *J* = 7.1 Hz, 3H). ^{13}C NMR (101 MHz, $CDCl_3$) δ 165.9, 165.1, 164.8, 151.8, 149.4, 148.2, 134.5, 133.1, 131.8, 131.7, 130.7, 128.9, 128.7, 127.8, 125.5, 123.0, 61.3, 33.6, 14.1. HRMS (ESI), *m/z* calcd for $C_{20}H_{15}NNaO_4$ $[M+Na]^+$ 356.0893 found 356.0891.

(8b*R/S*,12b*R/S*,15a*S/R*,15b*S/R*)-5,9-Dimethyl-2,14-diphenyl-14,15b-dihydro-1*H*-dibenzo[2,3:4,5]-azuleno[1,8*a-c*:7,8-*c'*]dipyrrole-1,3,13,15(2*H*,12b*H*,14*H*)-tetraone (2r), 7-methyl-2-phenylindeno-[1,2-*c*]pyrrole-1,3(2*H*,8*H*)-dione (3r) and compound 5: prepared according to the general procedure from diazo compound **1r** (1.0 mmol). After column chromatography pure indene **3r** and the mixture of dimers **2r** and **5** (129 mg, ratio 1.0:0.3) were obtained. Individual compounds **2r** and **5** were isolated by HPLC (eluent – *n*-hexane–ethyl acetate (from 10% to 30% of ethyl acetate)).

Compound 2r: Yield: 85 mg (31%). Amorphous solid. ^{1}H NMR (400 MHz, $CDCl_3$) δ 8.14 (d, *J* = 1.9 Hz, 1H), 7.52 (d, *J* = 7.6 Hz, 1H), 7.44 – 7.39 (m, 3H), 7.36 (t, *J* = 7.6 Hz, 1H), 7.31 (m, 2H), 7.26 (m, 1H), 7.23 (m, 1H), 7.18 (m, 3H), 7.12 (m, 1H), 6.97 (m, 2H), 6.41 (m, 1H), 5.20 (s, 1H), 4.70 (s, 1H), 3.71 (d, *J* = 1.9 Hz, 1H), 2.49 (s, 3H), 2.05 (s, 3H). ^{13}C NMR (101 MHz, $CDCl_3$) δ 176.8, 174.1, 173.0, 167.0, 139.3, 139.2, 138.6, 137.1, 136.7, 134.7, 133.3, 131.4, 131.2, 130.5, 130.1, 129.9, 129.4, 129.1,

129.0, 128.9, 128.8, 128.7, 126.6, 126.5, 126.4, 122.7, 72.1, 55.3, 51.8, 46.7, 19.9, 19.2. HRMS (ESI), *m/z* calcd for $C_{36}H_{27}N_2O_4$ $[M+H]^+$ 551.1965 found 551.1969

Compound 3r: Yield: 83 mg (30%). Yellow solid, m.p. 183.5 – 185.4 °C. ^1H NMR (400 MHz, $CDCl_3$) δ 7.75 (d, *J* = 7.5 Hz, 1H), 7.54 – 7.48 (m, 2H), 7.47 – 7.38 (m, 4H), 7.29 (d, *J* = 7.1 Hz, 1H), 3.73 (s, 2H), 2.47 (s, 3H). ^{13}C NMR (101 MHz, $CDCl_3$) δ 165.7, 164.7, 151.6, 148.5, 146.9, 134.9, 134.0, 132.1, 130.2, 129.1, 128.2, 127.6, 126.5, 120.6, 32.5, 18.8. HRMS (ESI), *m/z* calcd for $C_{18}H_{14}NO_2$ $[M+H]^+$ 276.1019 found 276.1020

Compound 5: Yield: 22 mg (8%). White solid; m.p. 238.6 – 240.9 °C. ^1H NMR (400 MHz, $CDCl_3$) δ 7.69 (d, *J* = 7.6 Hz, 2H), 7.47 – 7.42 (m, 4H), 7.40 – 7.34 (m, 4H), 7.30 – 7.27 (m, 4H), 7.25 (d, *J* = 7.6 Hz, 2H), 5.88 (s, 2H), 4.19 (s, 2H), 2.42 (s, 6H). ^{13}C NMR (101 MHz, $CDCl_3$) δ 175.3, 174.5, 141.1, 139.8, 133.9, 131.4, 130.5, 129.3, 129.0, 128.7, 126.2, 123.7, 55.5, 52.9, 52.2, 19.9. HRMS (ESI), *m/z* calcd for $C_{36}H_{26}N_2NaO_4$ $[M+Na]^+$ 573.1785 found 573.1785.

2. Crystallographic data for compounds 2a and 5

X-ray single crystal analysis was performed on Rigaku XtaLAB Synergy-S diffractometer with monochromated CuKα radiation. Crystal growth was performed by slow evaporation of a solution in *n*-hexane/acetone mixture 1:1 at 5 °C. The crystal was kept at 100 K during data collection. Using Olex2[4], the structures were solved with the SHELXT[5] structure solution program using Intrinsic Phasing and refined with the SHELXL[6,7] refinement package using Least Squares minimization. CCDC 2155454 (**2a**) and CCDC 2144354 (**5**) contain the supplementary crystallographic data for this paper. These data can be obtained free of charge from The Cambridge Crystallographic Data Centre via https://www.ccdc.cam.ac.uk/.

**Table S1.** Crystal data and ORTEP representation for **2a (2155454)**	
**Empirical Formula**	$C_{34}H_{22}N_2O_4$
**Formula weight**	522.53
**Temperature, K**	100.15
**Crystal system**	monoclinic
**Space group**	$P2_1$
**a/Å**	9.64990(10)
**b/Å**	13.35850(10)
**c/Å**	9.85390(10)
**α/°**	90
**β/°**	91.5160(10)
**γ/°**	90
**Volume/Å^3**	1269.80(2)
**Z**	2
**ρ_{calc}g/cm^3**	1.367
**μ/mm^{-1}**	0.730
**F(000)**	544.0
**Crystal size/mm^3**	0.12 × 0.12 × 0.1
**Radiation**	CuKα (λ = 1.54184)
**2Θ range for data collection/°**	8.978 to 160.002
**Index ranges**	$-12 \le h \le 11$, $-16 \le k \le 16$, $-12 \le l \le 12$
**Reflections collected**	18324
**Independent reflections**	5274 [R_{int} = 0.0329, R_{sigma} = 0.0279]
**Data/restraints/parameters**	5274/1/361
**Goodness-of-fit on F^2**	1.065
**Final R indexes [I>=2σ (I)]**	R_1 = 0.0305, wR_2 = 0.0765
**Final R indexes [all data]**	R_1 = 0.0310, wR_2 = 0.0770
**Largest diff. peak/hole / e Å^{-3}**	0.15/-0.20

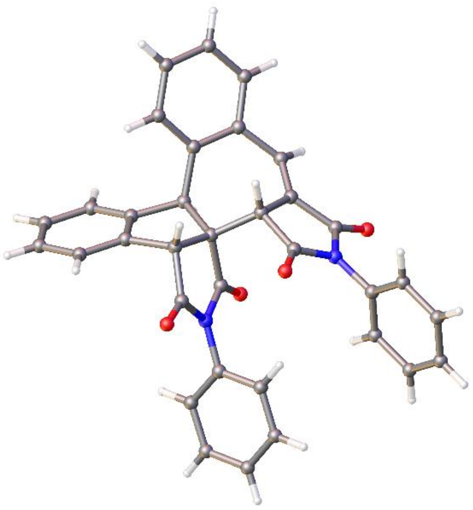

Figure S1. ORTEP representation of compound **2a** (thermal ellipsoids are shown at 50% probability)

**Table S2.** Crystal data and ORTEP representation for **5 (2144354)**	
**Empirical Formula**	$C_{36}H_{26}N_2O_4$
**Formula weight**	550.61
**Temperature, K**	100(2)
**Crystal system**	monoclinic
**Space group**	I 2/a
**a/Å**	13.9998(2)
**b/Å**	13.5871(2)
**c/Å**	17.2134(3)
**α/°**	90
**β/°**	108.553(2)
**γ/°**	90
**Volume/Å^3**	3104.11(9)
**Z**	4
**ρ_{calc}g/cm^3**	1.302
**μ/mm^{-1}**	0.695
**F(000)**	1280
**Crystal size/mm^3**	0.18 × 0.16 × 0.08
**Radiation**	CuKα (λ = 1.54184)
**2Θ range for data collection/°**	4.2360 to 79.3520
**Index ranges**	$-17 \leq h \leq 17$, $-10 \leq k \leq 17$, $-21 \leq l \leq 21$
**Reflections collected**	11804
**Independent reflections**	3271 [R_{int} = 0.0405, R_{sigma} = 0.0370]
**Data/restraints/parameters**	3271/0/211
**Goodness-of-fit on F^2**	1.074
**Final R indexes [I>=2σ (I)]**	R_1 = 0.0370, wR_2 = 0.0942
**Final R indexes [all data]**	R_1 = 0.0405, wR_2 = 0.0968
**Largest diff. peak/hole / e Å^{-3}**	0.283/-0.216

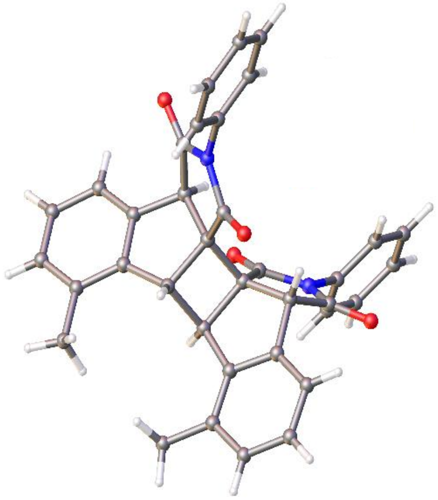

Figure S2. ORTEP representation of compound **5** (thermal ellipsoids are shown at 50% probability)

3. Biological data

Cell culture

A549 lung cancer cells and WI-26 VA4 lung epithelial-like cells were purchased from the ATCC. A549 cells were maintained in F12-K (Corning, NY, USA) supplemented with 10% fetal bovine serum (Fetal Bovine Serum, qualified, Australia; Gibco, Loughborough, UK), penicillin (100 UI mL^{-1}), streptomycin (100 µg mL^{-1}), and GlutaMax (1.9 mM, Gibco, UK). WI-26 VA4 cells were maintained in Advanced MEM (Gibco, Loughborough, UK) supplemented with 5% fetal bovine serum (Fetal Bovine Serum, qualified, Australia, Gibco, UK), penicillin (100 UI mL^{-1}), streptomycin (100 µg mL^{-1}), and GlutaMax (1.87 mM, Gibco, Loughborough, UK). All cell lines were cultivated under a humidified atmosphere of 95% air/5% CO_2 at 37 °C. Subconfluent monolayers, in the log growth phase, were harvested by a brief treatment with TrypLE Express solution (Gibco, Loughborough, UK) in phosphate buffered saline (PBS, Capricorn Scientific, Germany) and washed three times in serum-free PBS. The number of viable cells was determined by trypan blue exclusion.

MTT assay

The effects of the synthesized compounds on cell viability were determined using the MTT colorimetric test. All examined cells were diluted with the growth medium to 3.5×10^4 cells per mL and aliquots (7×10^3 cells per 200 µL) were placed in individual wells of 96-well plates (Eppendorf, Hamburg, Germany) and incubated for 24 h. The next day, the cells were treated with the synthesized compounds separately at a concentration of 250.0 µM and at various diluted concentrations for determination of IC_{50} values and incubated for 72 h at 37 °C in a 5% CO_2 atmosphere. Each compound was tested in triplicate. After incubation, the cells were treated with 40 µL MTT solution (3-(4,5-dimethylthiazol-2-yl)-2,5-diphenyltetrazolium bromide, 5 mg mL^{-1} in PBS) and incubated for 4 h. After additional 4 h of incubation, the medium with MTT was removed and DMSO (150 µL) was added to dissolve the formazan crystals. The plates were shaken for 10 min. The optical density of each well was determined at 560 nm using GloMax Multi+ (Promega, Madison, WI, USA) microplate reader. Each of the tested compounds was evaluated for cytotoxicity in three separate experiments. All stock solutions for biological evaluations were prepared by dissolving the synthesized compounds in DMSO.

4. References

2. Chupakhin, E. G.; Kantin, G. P.; Dar'in, D. V.; Krasavin, M. Convenient preparation of (*E*)-3-arylidene-4-diazopyrrolidine-2,5-diones in array format. *Mendeleev Commun.* **2021**, *31*, 36-38.
3. Chupakhin, E.; Gecht, M.; Ivanov, A.; Kantin, G.; Dar'in, D.; Krasavin, M. (*E*)-3-Arylidene-4-diazopyrrolidine-2,5-diones: Preparation and Use in Rh^{II}-Catalyzed X–H Insertion Reactions towards Novel, Medicinally Important Michael Acceptors. *Synthesis* **2021**, *53*, 1292-1300.
4. Vepreva, A.; Kantin, G.; Krasavin, M.; Dar'in. A General Way to Spiro-Annulated 2-Benzoxepines via $Rh_2(esp)_2$-catalyzed [5+2] Cycloaddition of Diazo Arylidene Succinimides to Ketones. *Synthesis* **2022**. doi: 10.1055/s-0037-1610790.
5. Dolomanov, O. V; Bourhis, L. J.; Gildea, R. J.; Howard, J. A. K.; Puschmann, H. *OLEX2*: A Complete Structure Solution, Refinement and Analysis Program. *J. Appl. Crystallogr.* **2009**, *42*, 339–341.
6. Sheldrick, G. M. SHELXT-Integrated Space-Group and Crystal-Structure Determination. *Acta Crystallogr. Sect. A* **2015**, A71, 3-8.
7. Sheldrick, G. M. A Short History of *SHELX*. *Acta Crystallogr. Sect. A* **2008**, *64*, 112–122.
8. Sheldrick, G. M. Crystal Structure Refinement with *SHELXL*. *Acta Crystallogr. Sect. C* **2015**, *71*, 3–8.

5. Copies of ^{1}H, ^{13}C, ^{19}F NMR and NOESY NMR spectra

Copies of ^{1}H (400.13 MHz, $CDCl_3$) and $^{13}C\{^{1}H\}$ (100.61 MHz, $CDCl_3$) spectra of **2a**

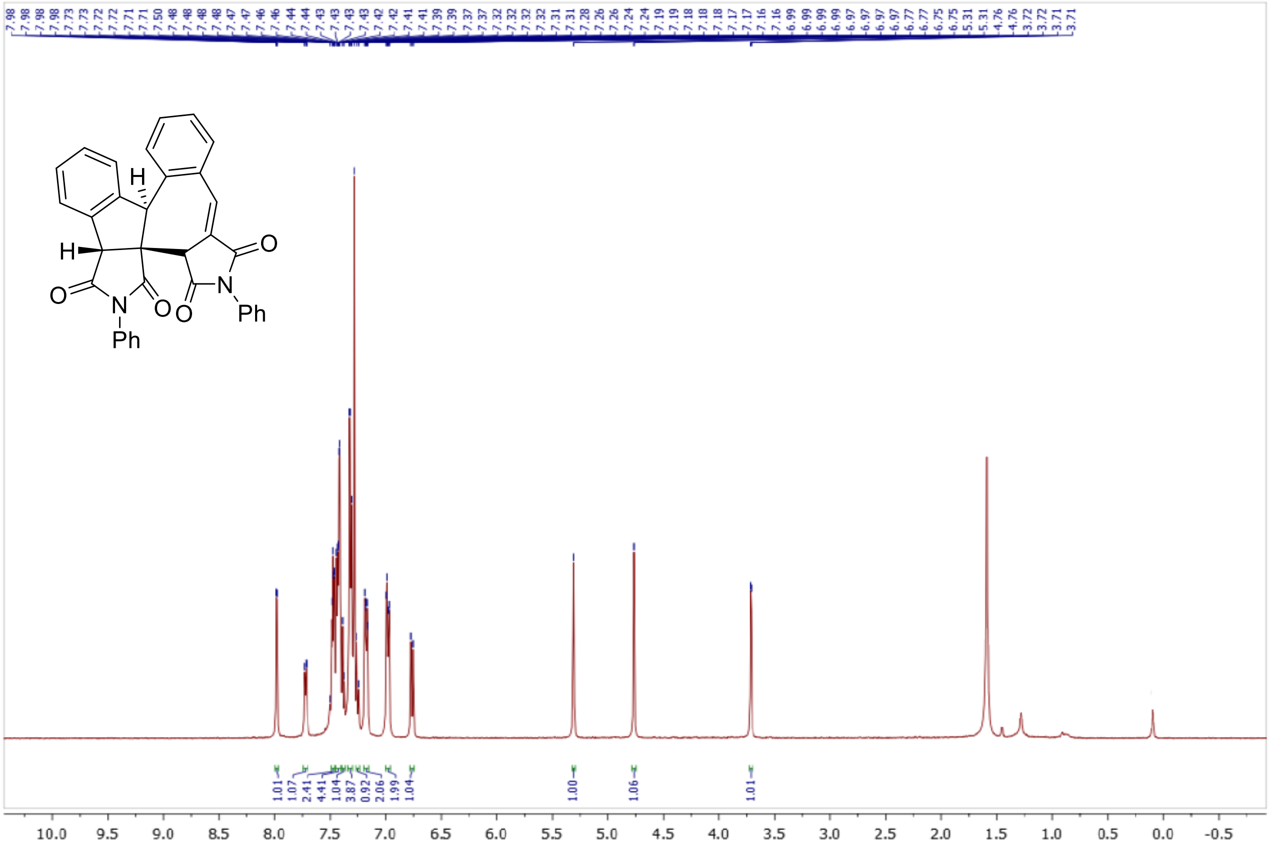

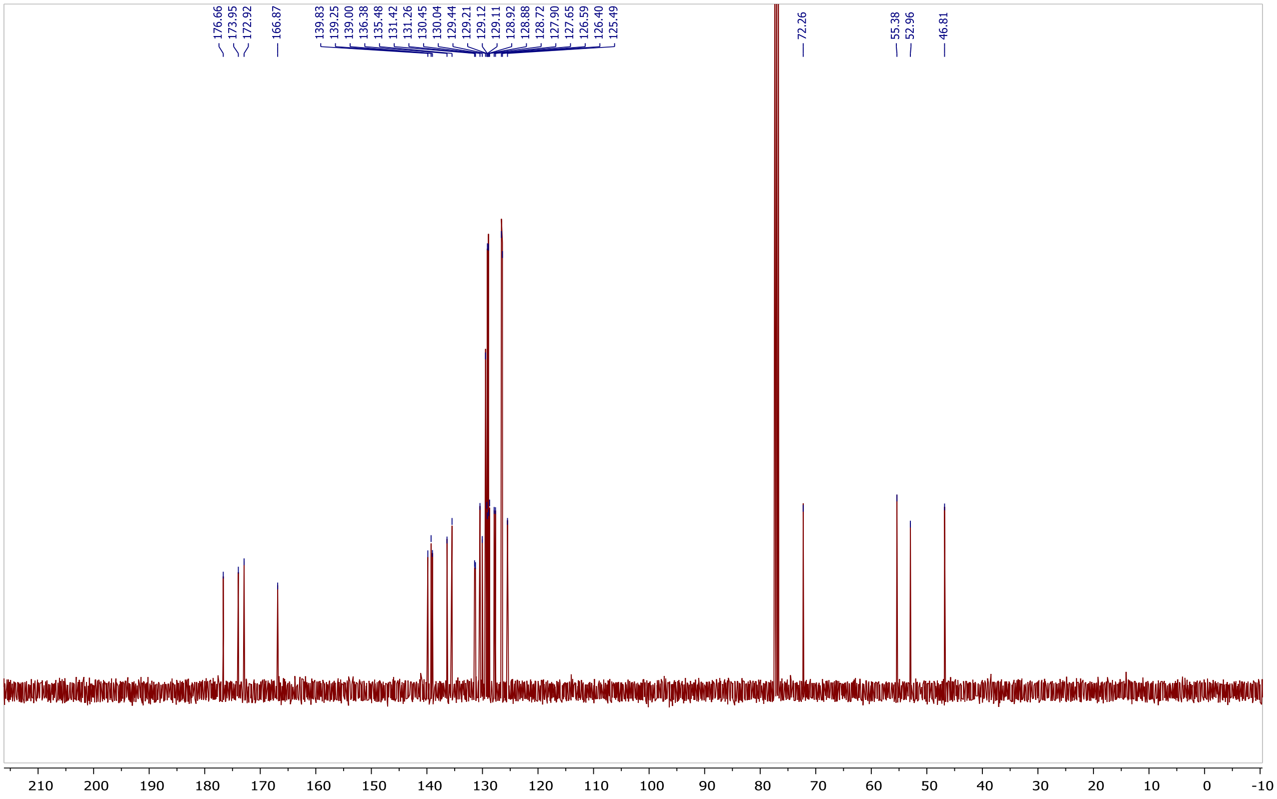

Copies of ^{1}H (400.13 MHz, $CDCl_3$) and $^{13}C\{^{1}H\}$ (100.61 MHz, $CDCl_3$) spectra of **3a**

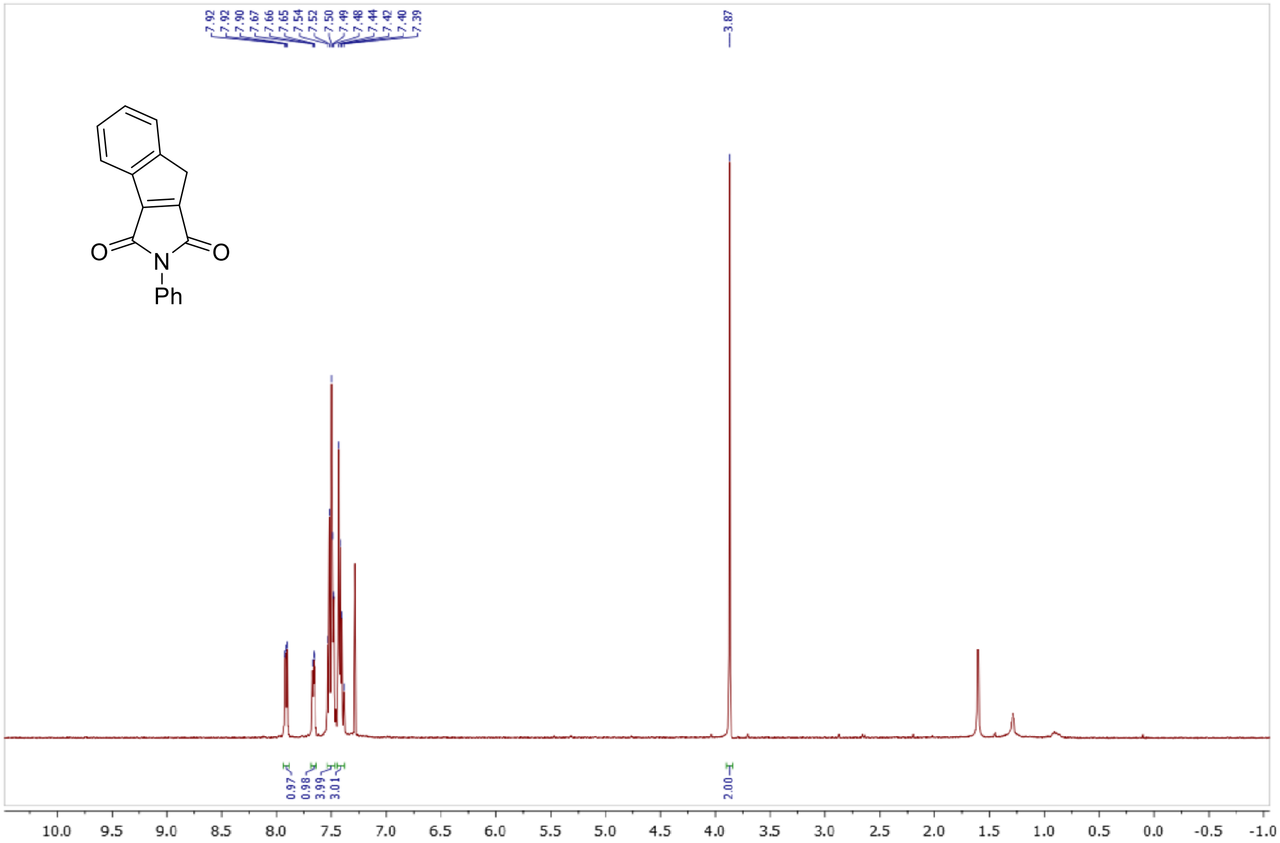

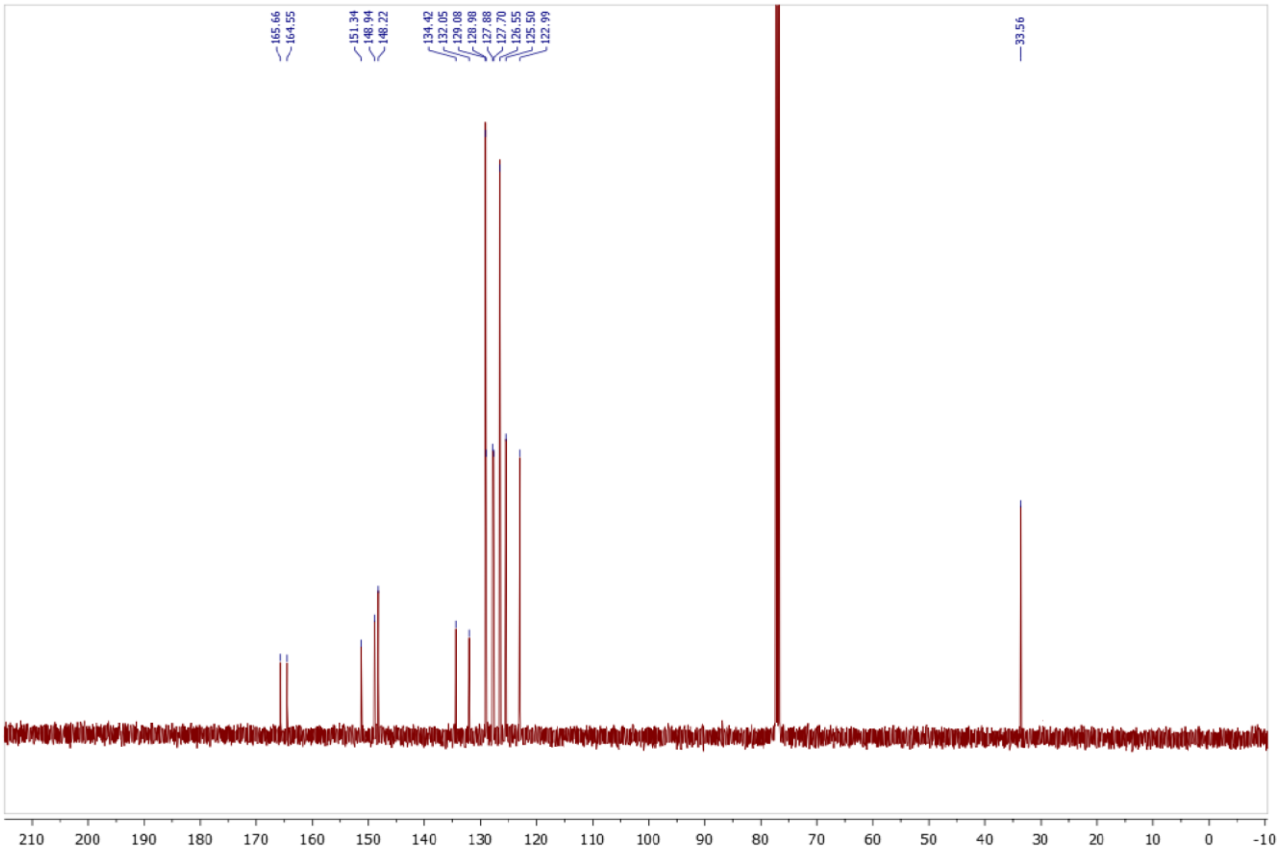

Copies of ^{1}H (400.13 MHz, $CDCl_3$) and $^{13}C\{^{1}H\}$ (100.61 MHz, $CDCl_3$) spectra of **2b**

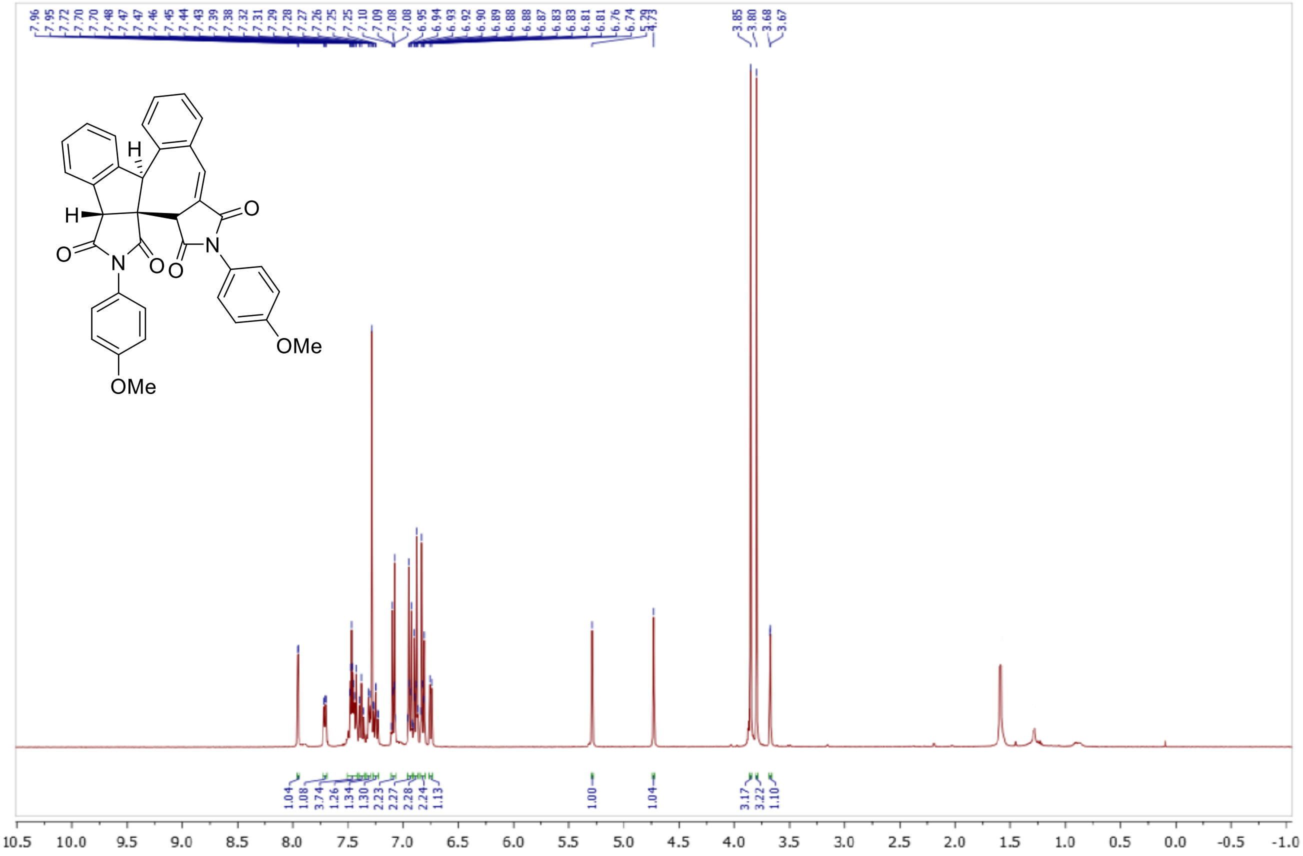

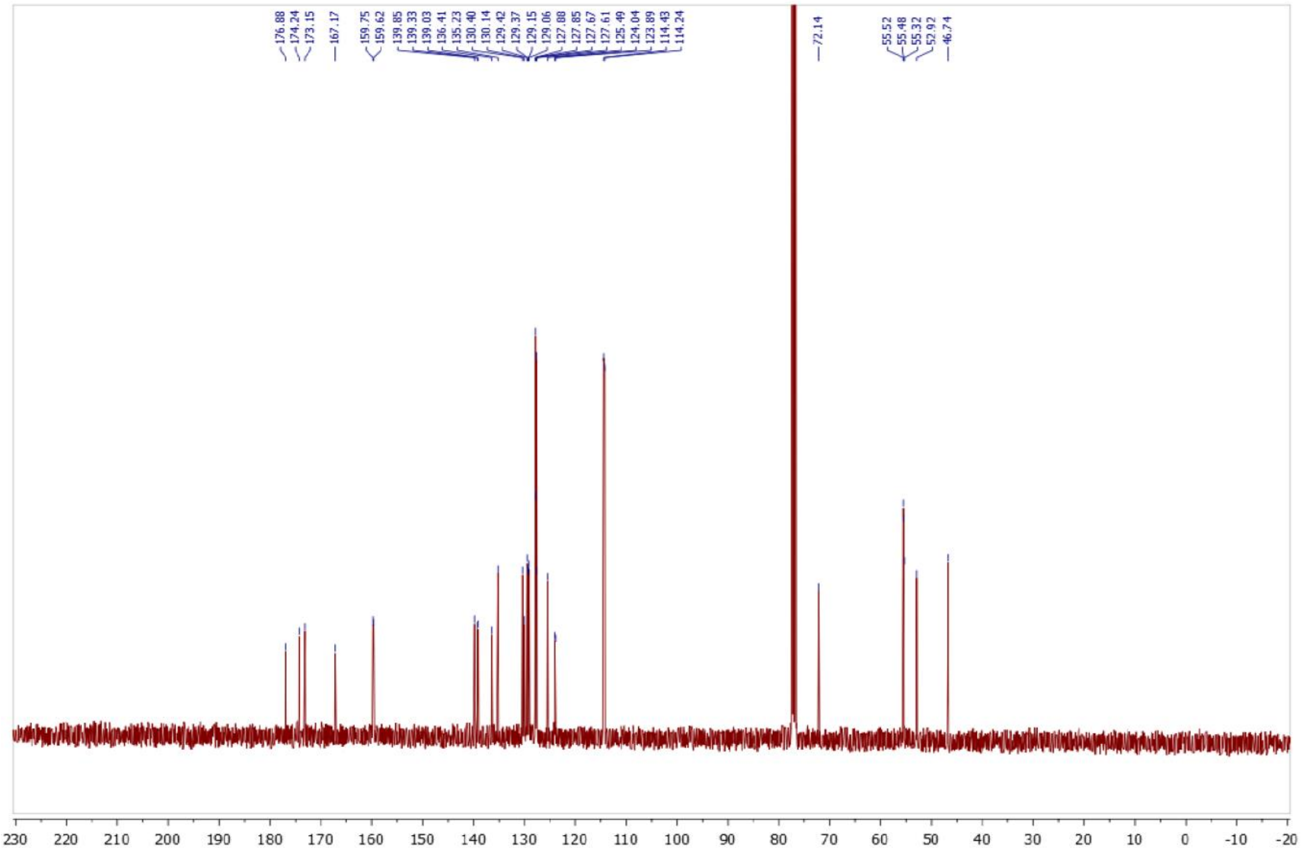

Copies of ^{1}H (400.13 MHz, $CDCl_3$) and $^{13}C\{^{1}H\}$ (100.61 MHz, $CDCl_3$) spectra of **2c**

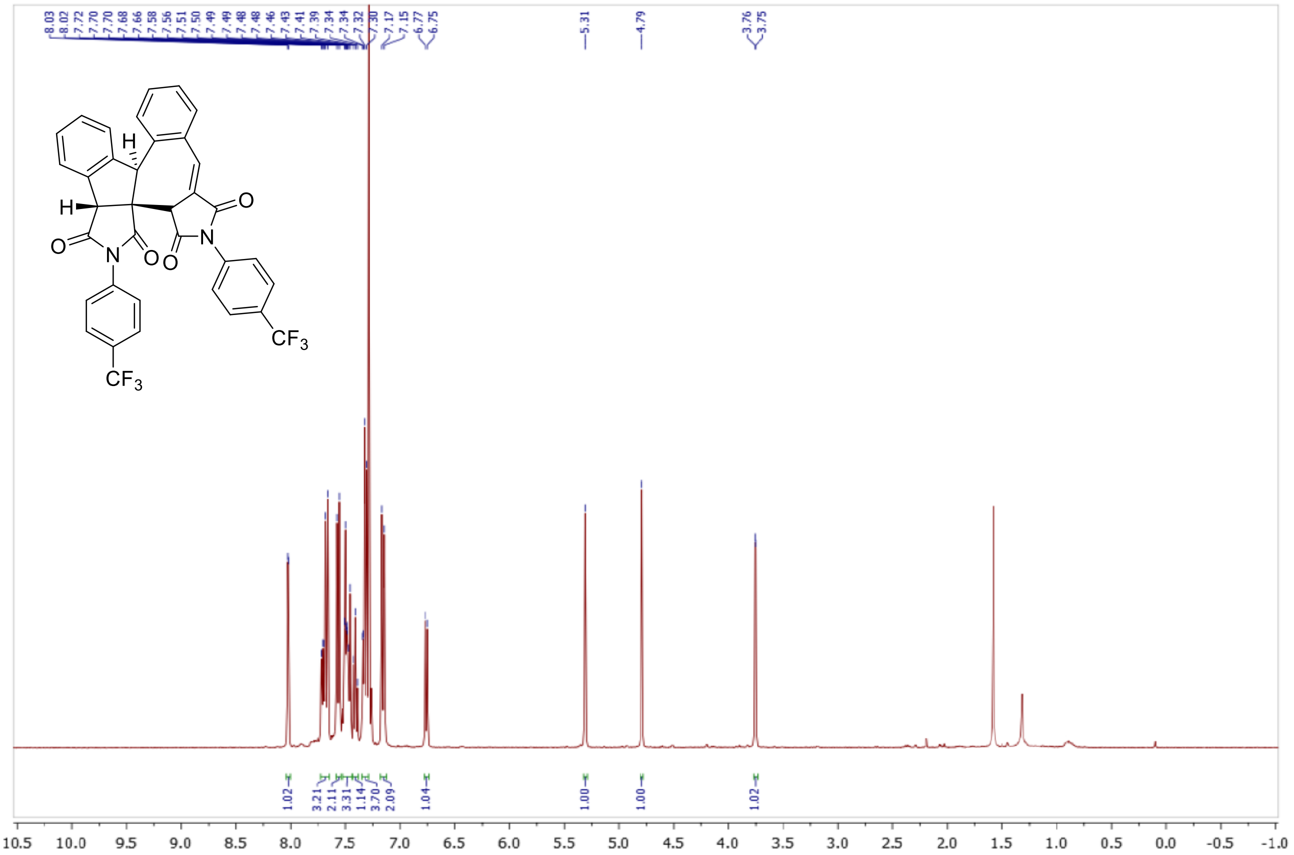

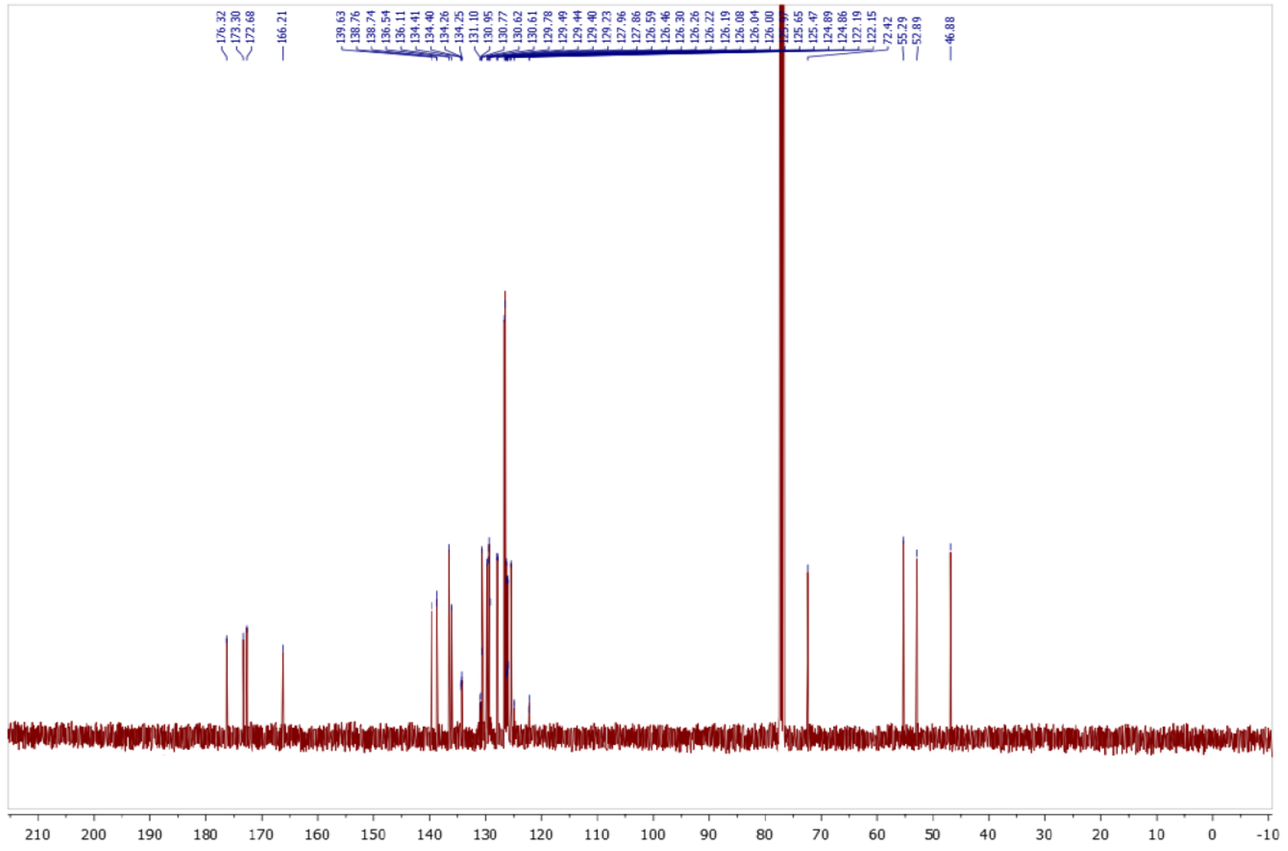

Copy of $^{19}F\{^1H\}$ (376.50 MHz, $CDCl_3$) spectrum of **2c**

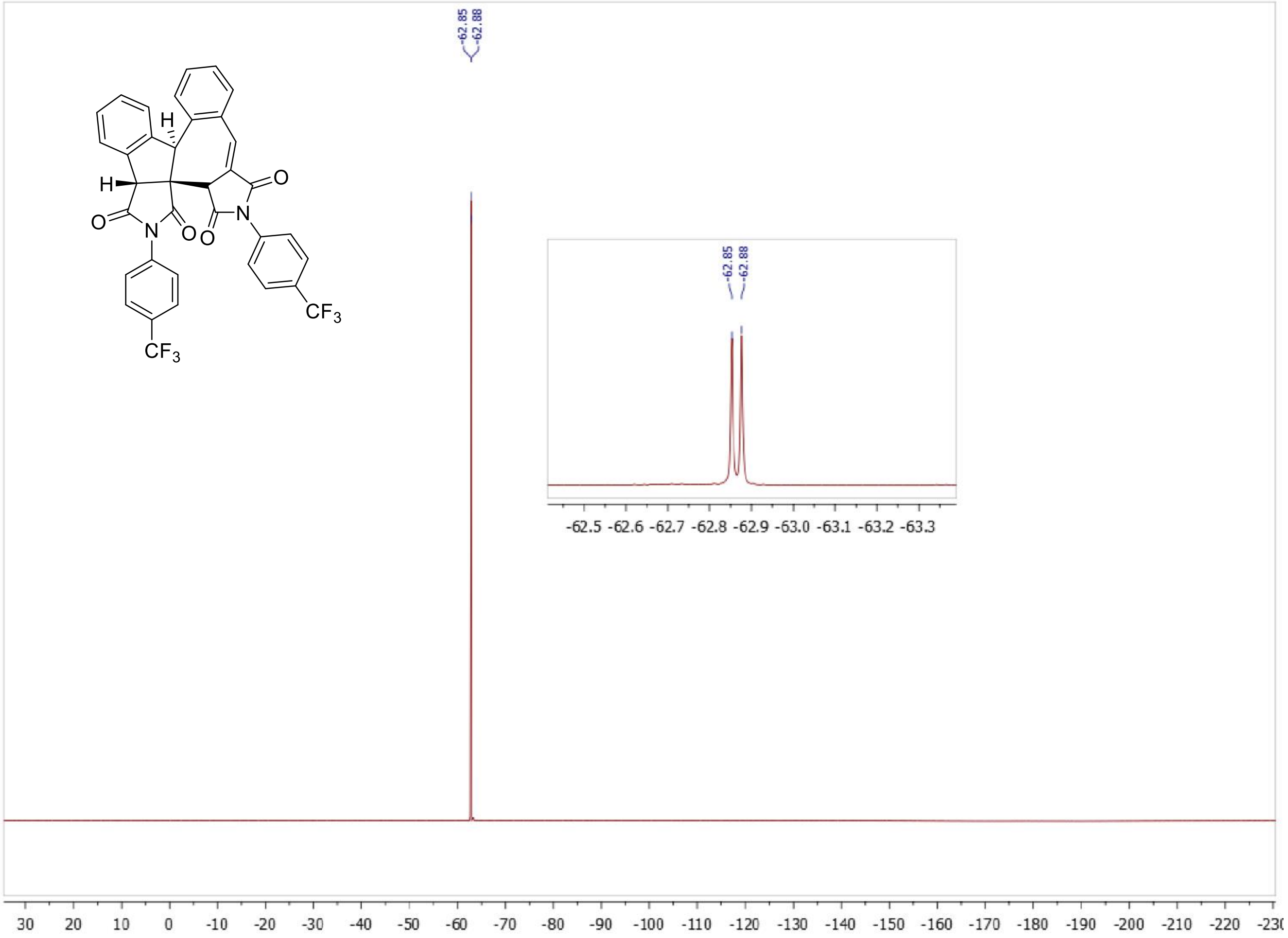

Copies of ^{1}H (400.13 MHz, $CDCl_3$) and $^{13}C\{^{1}H\}$ (100.61 MHz, $CDCl_3$) spectra of **2d**

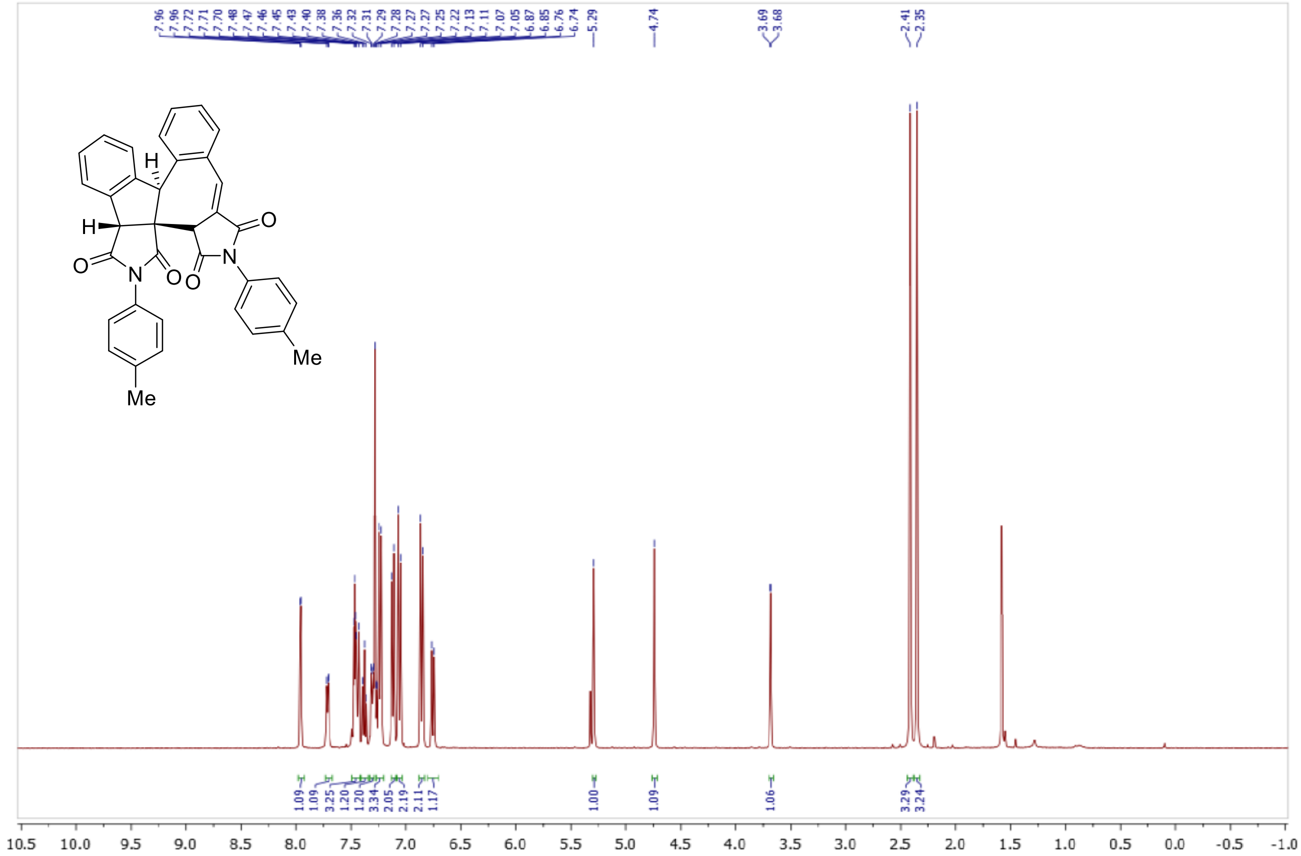

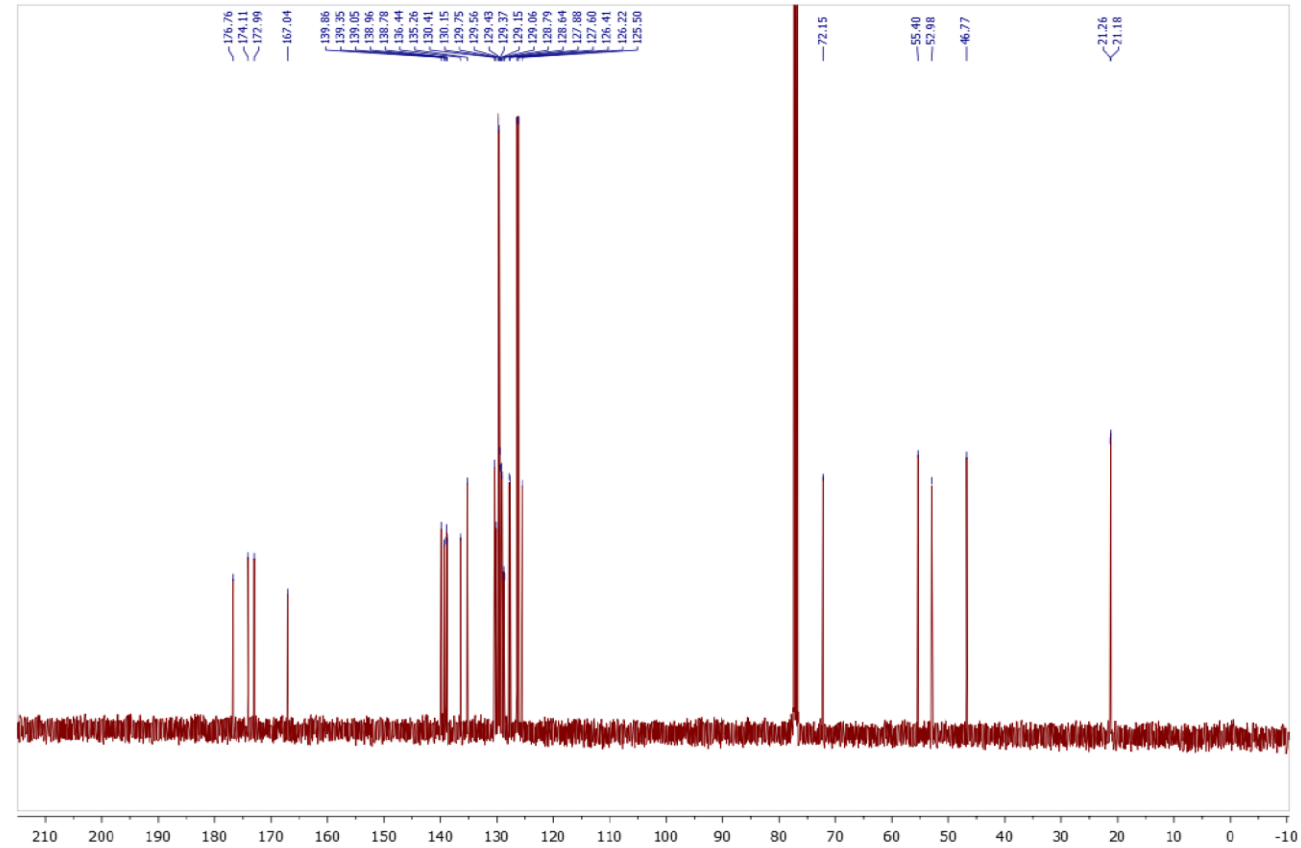

Copies of ^{1}H (400.13 MHz, $CDCl_3$) and $^{13}C\{^{1}H\}$ (100.61 MHz, $CDCl_3$) spectra of **2e**

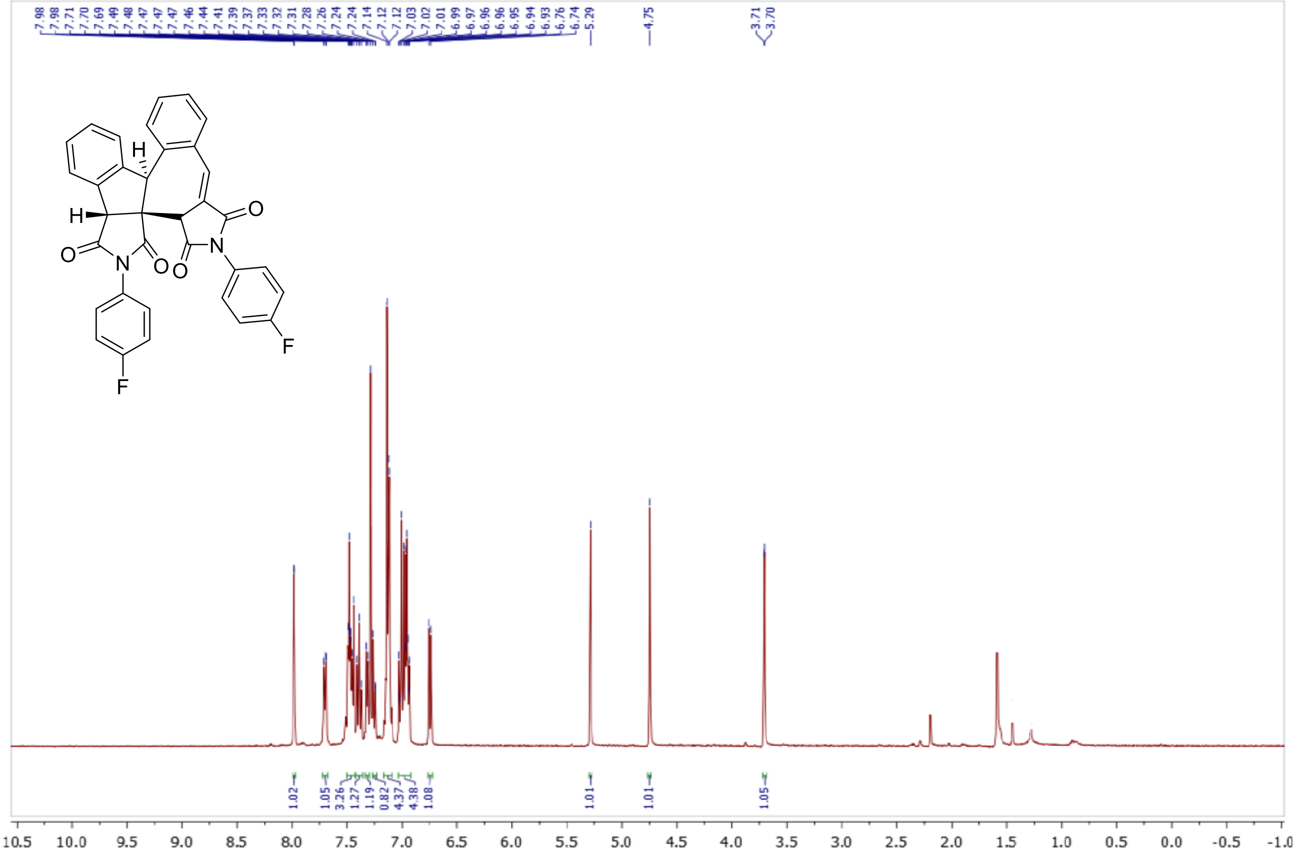

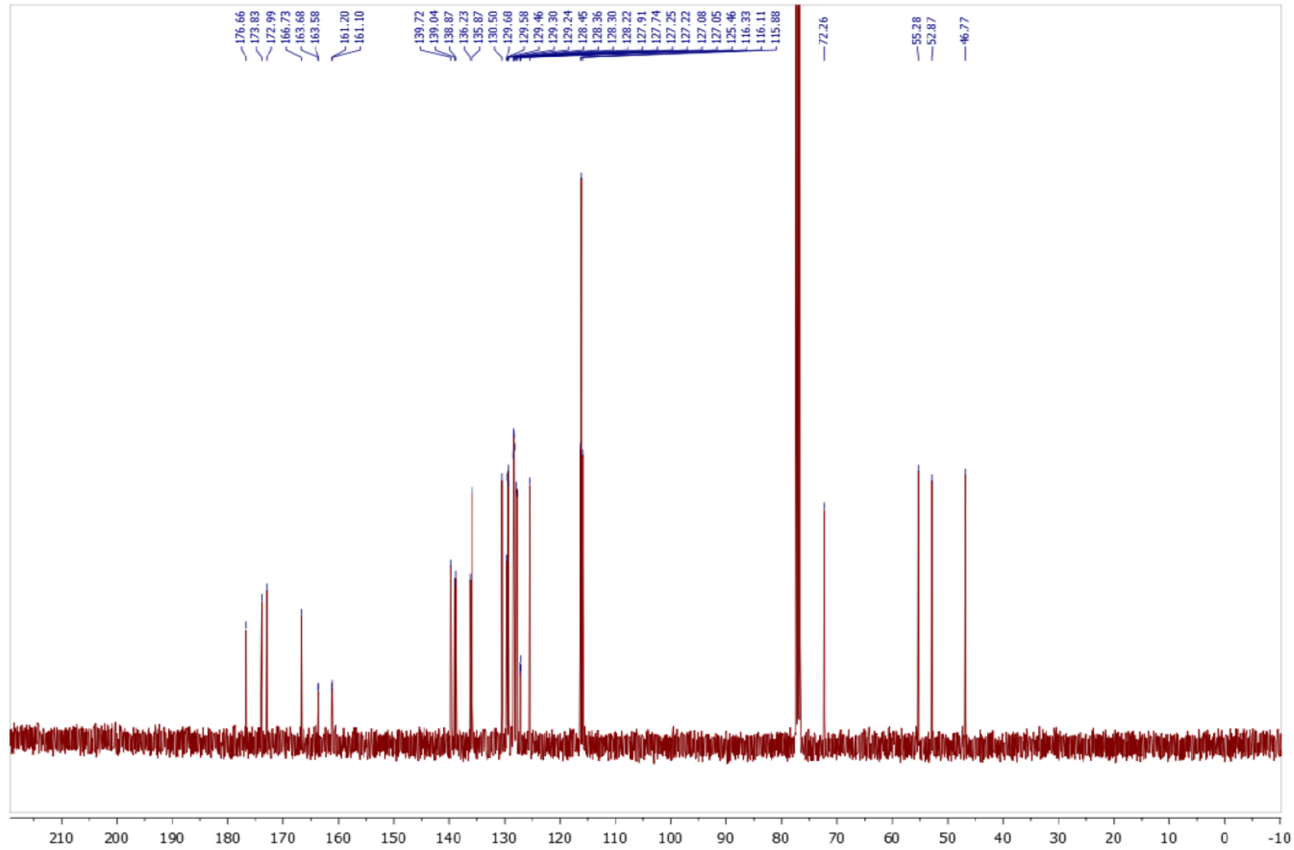

Copy of $^{19}F\{^{1}H\}$ (376.50 MHz, $CDCl_3$) spectrum of **2e**

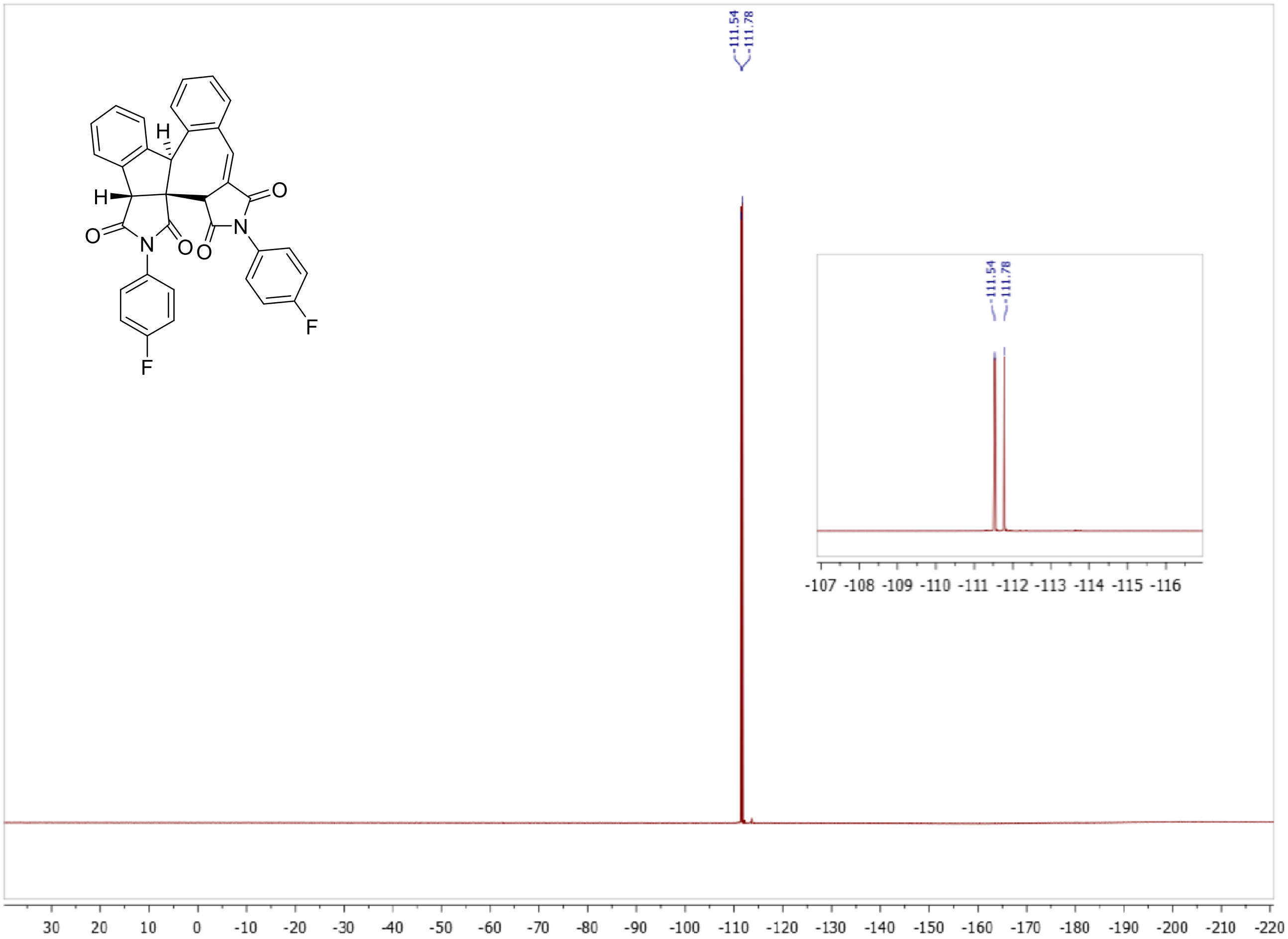

Copies of ^{1}H (400.13 MHz, $CDCl_3$) and ^{13}C{^{1}H} (100.61 MHz, $CDCl_3$) spectra of **2h**

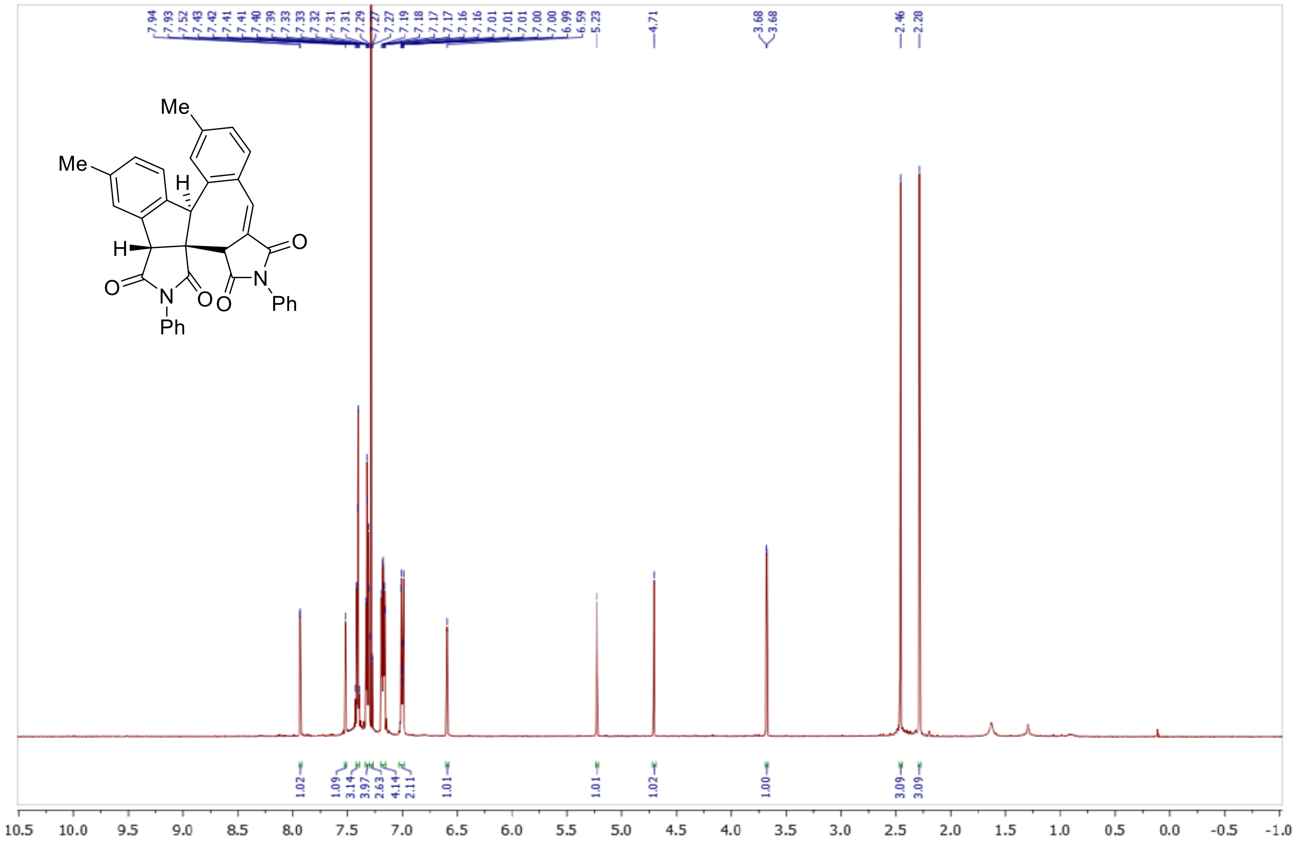

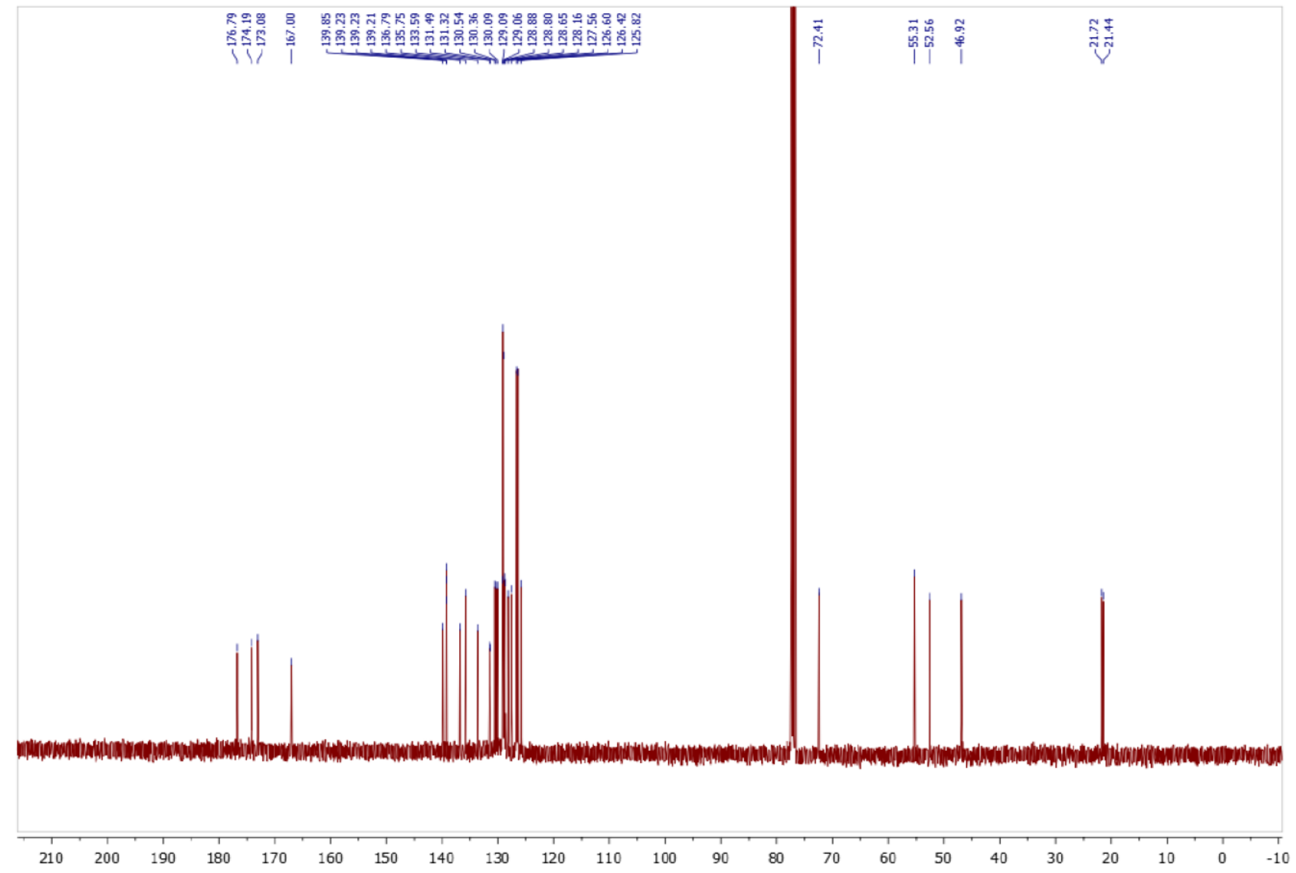

Copies of ^{1}H (400.13 MHz, $CDCl_3$) and $^{13}C\{^{1}H\}$ (100.61 MHz, $CDCl_3$) spectra of **2i**

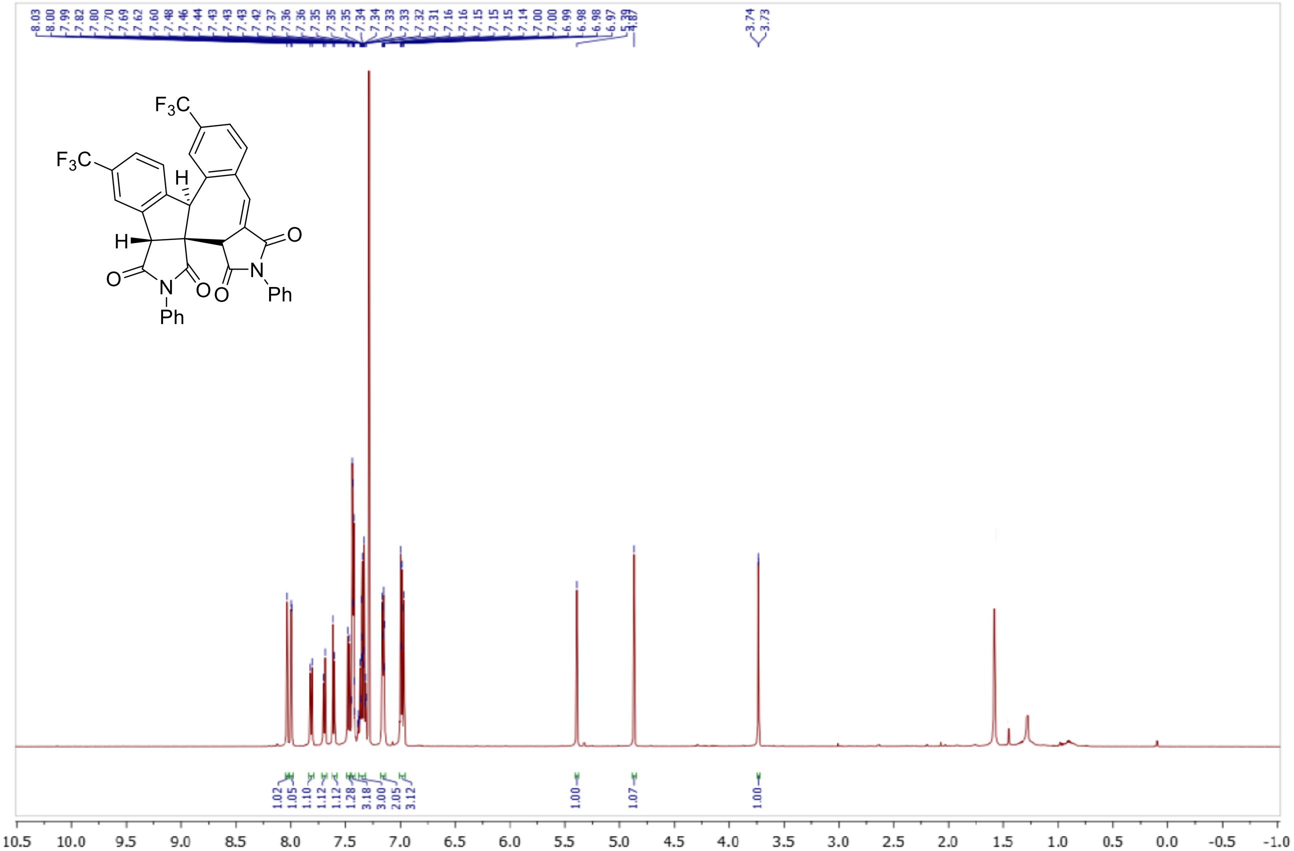

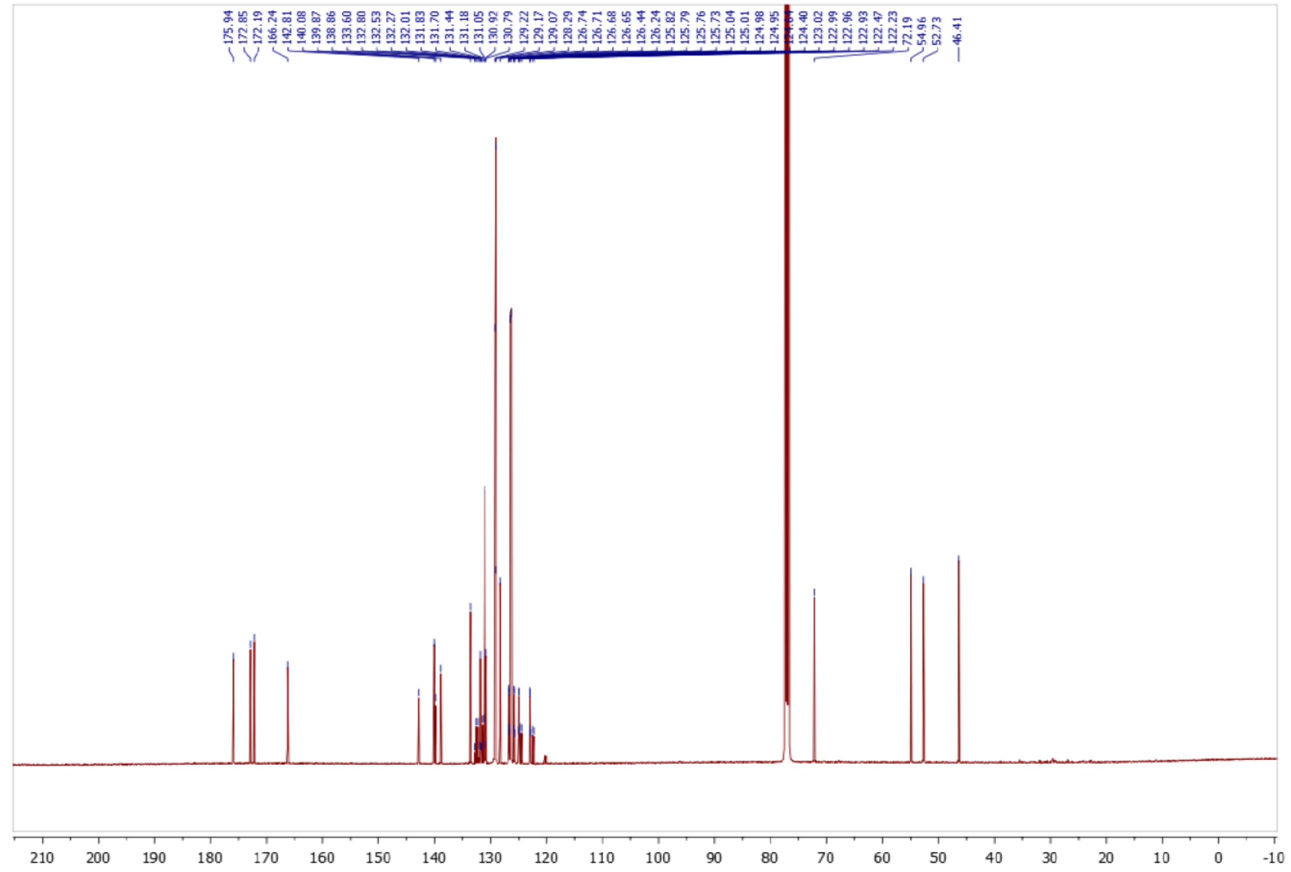

Copy of $^{19}F\{^{1}H\}$ (376.50 MHz, $CDCl_3$) spectrum of **2i**

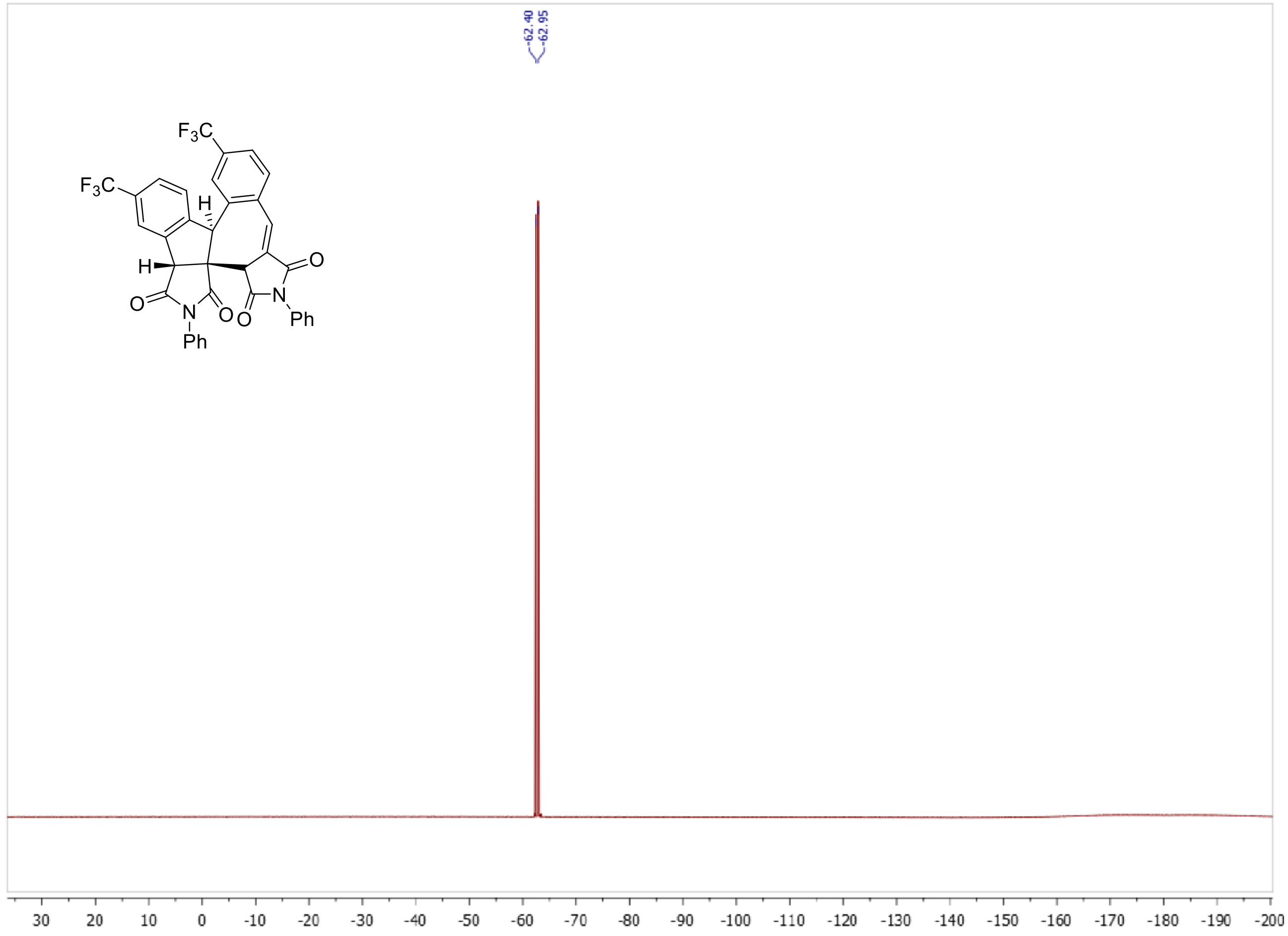

Copies of ^{1}H (400.13 MHz, $CDCl_3$) and $^{13}C\{^{1}H\}$ (100.61 MHz, $CDCl_3$) spectra of **2j**

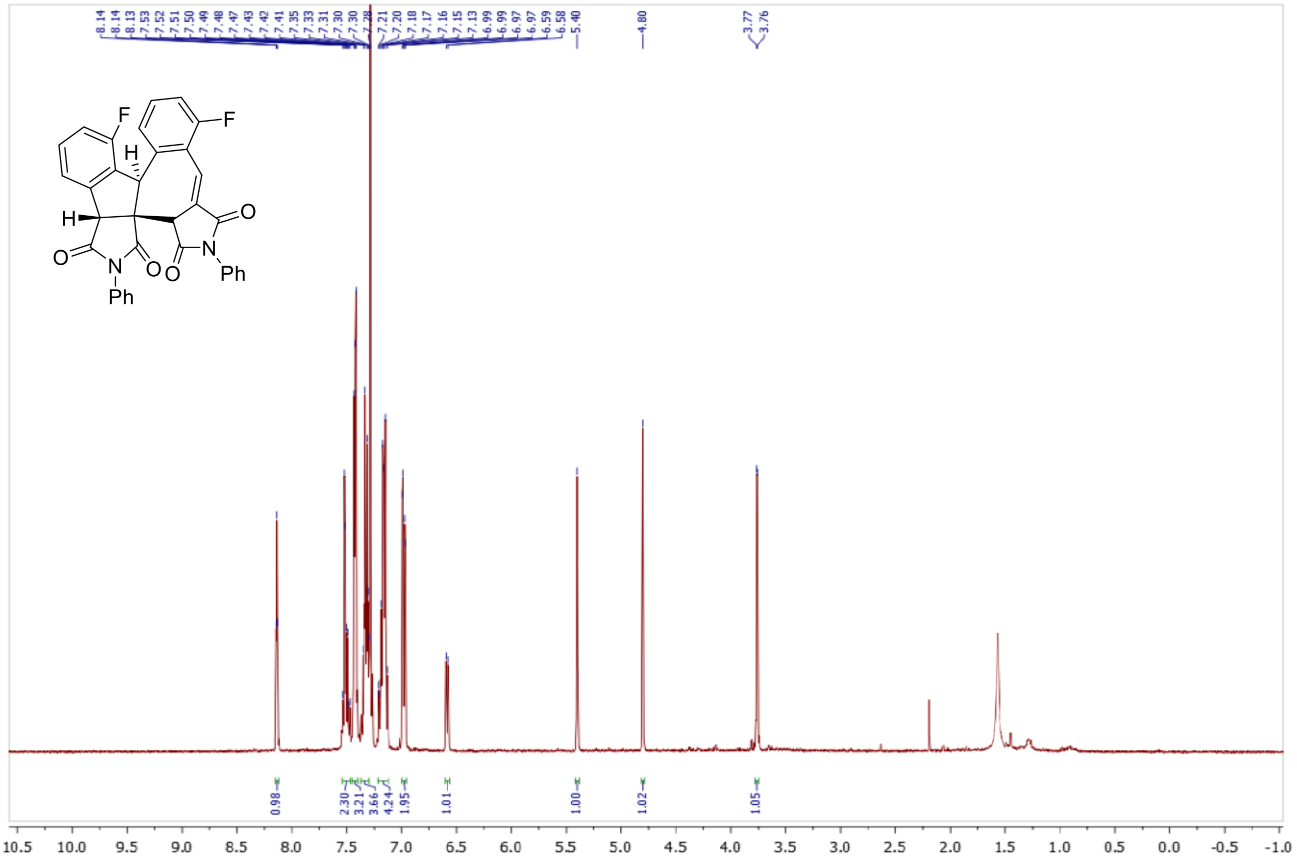

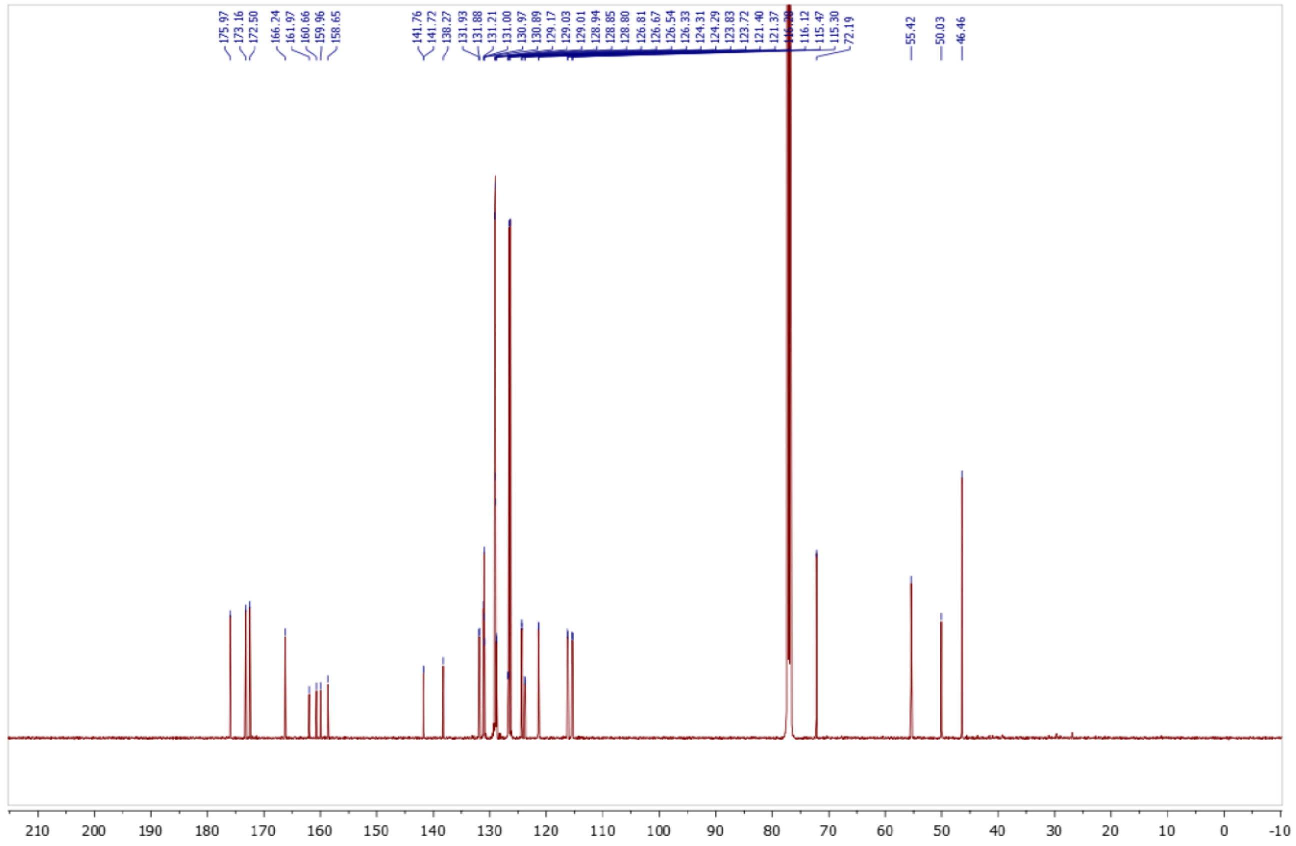

Copy of $^{19}F\{^{1}H\}$ (376.50 MHz, $CDCl_3$) spectrum of **2j**

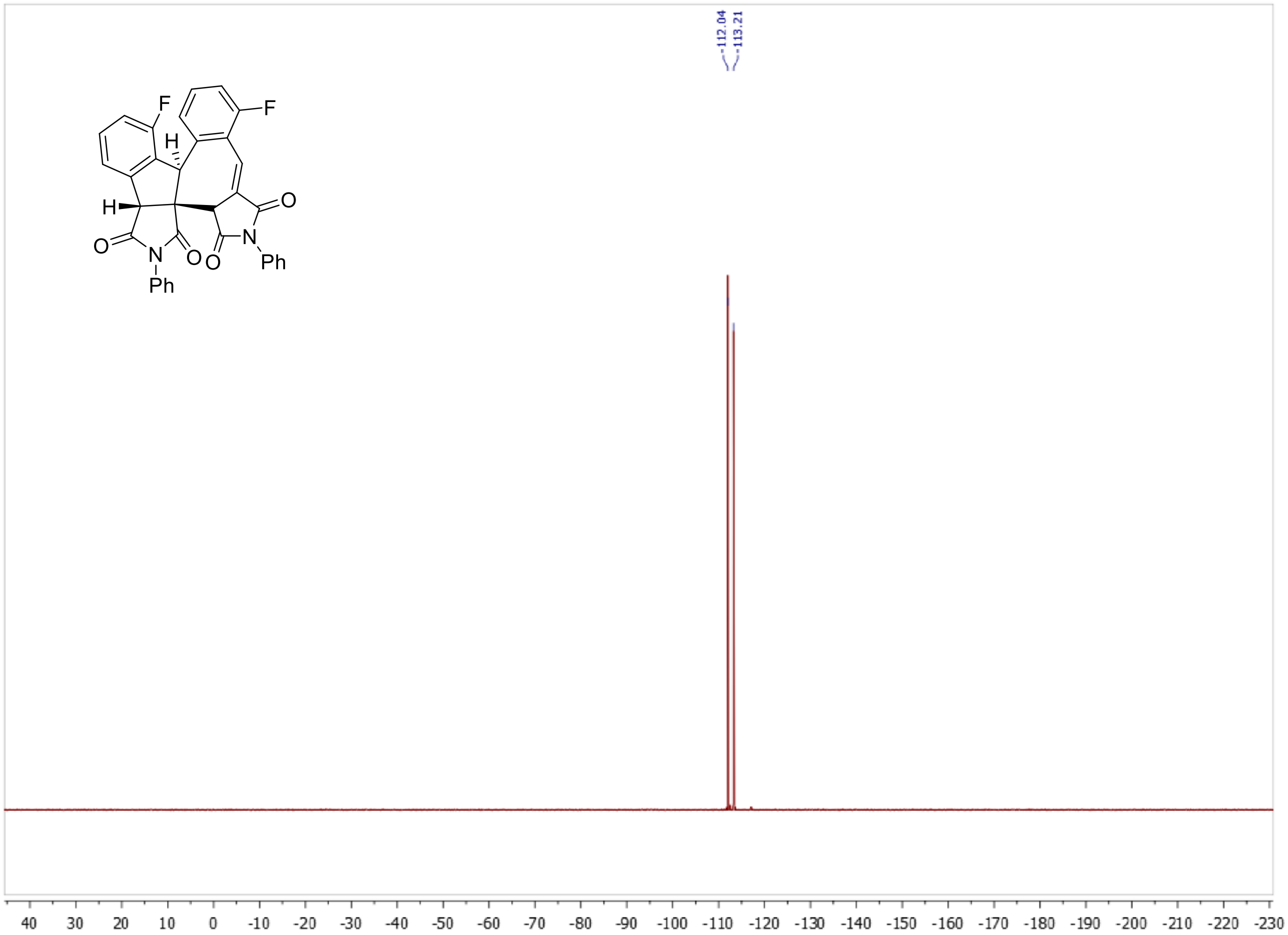

Copies of ^{1}H (400.13 MHz, $CDCl_3$) and $^{13}C\{^{1}H\}$ (100.61 MHz, $CDCl_3$) spectra of **2k**

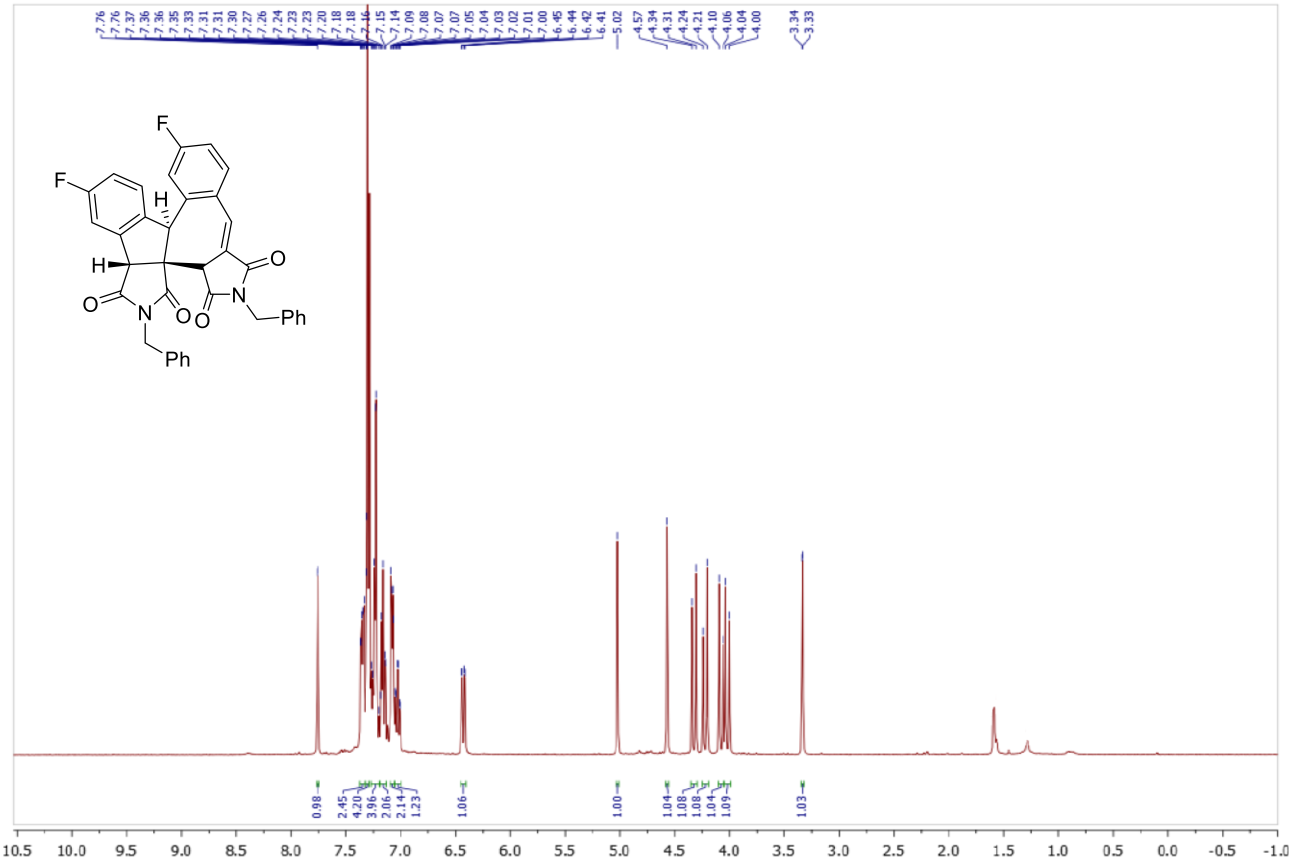

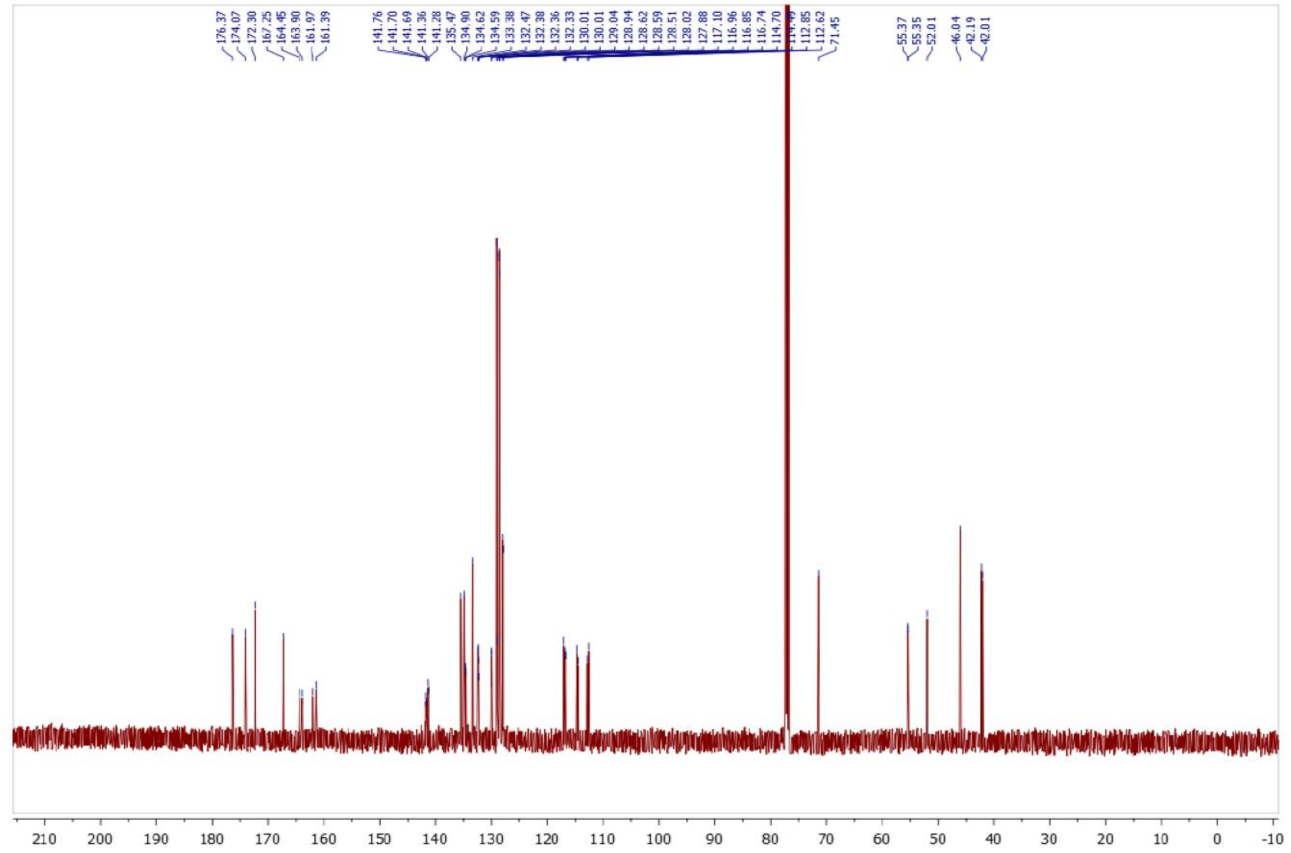

Copy of $^{19}F\{^{1}H\}$ (376.50 MHz, $CDCl_3$) spectrum of **2k**

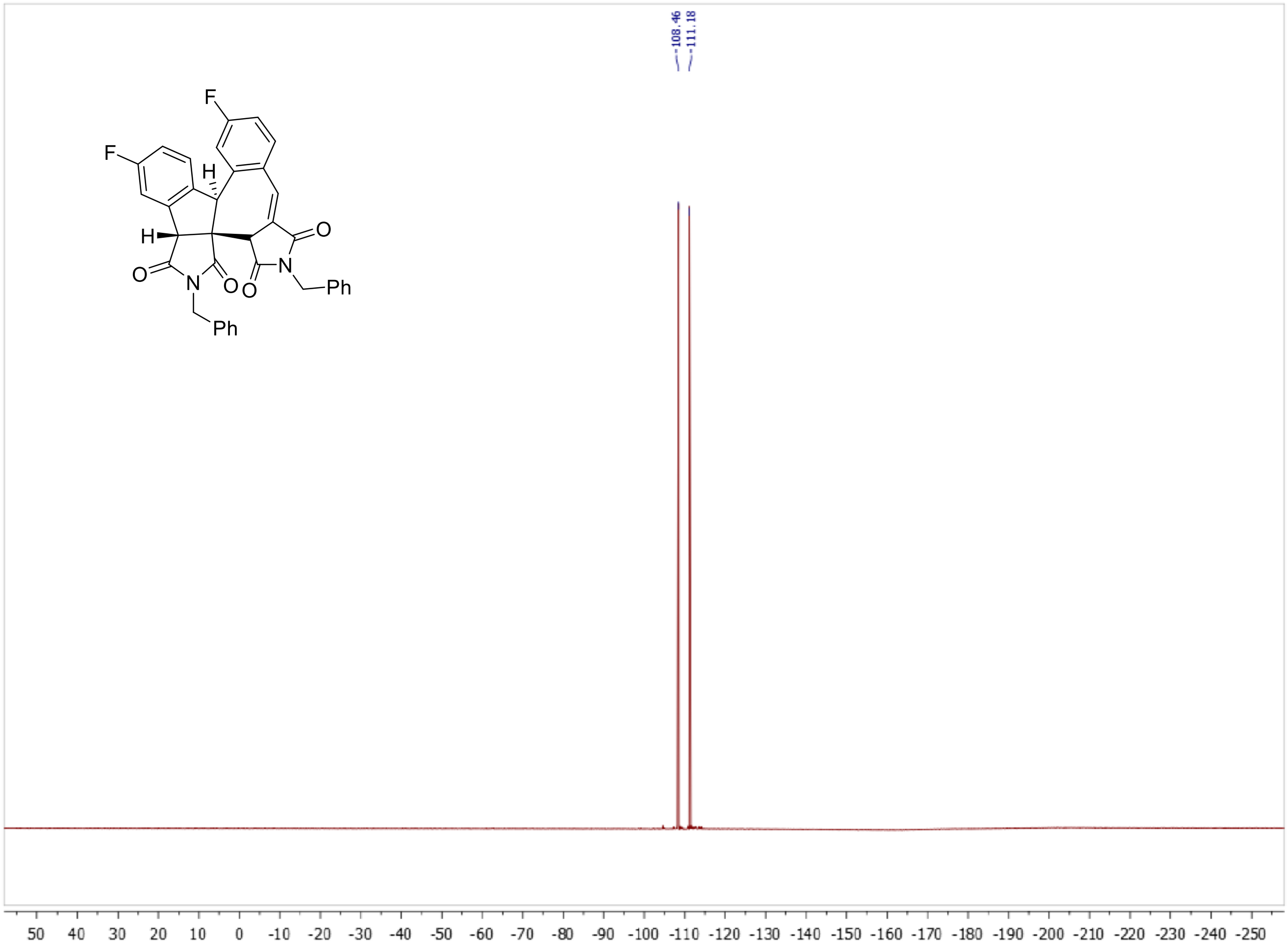

Copies of ^{1}H (400.13 MHz, $CDCl_3$) and $^{13}C\{^{1}H\}$ (100.61 MHz, $CDCl_3$) spectra of **4k**

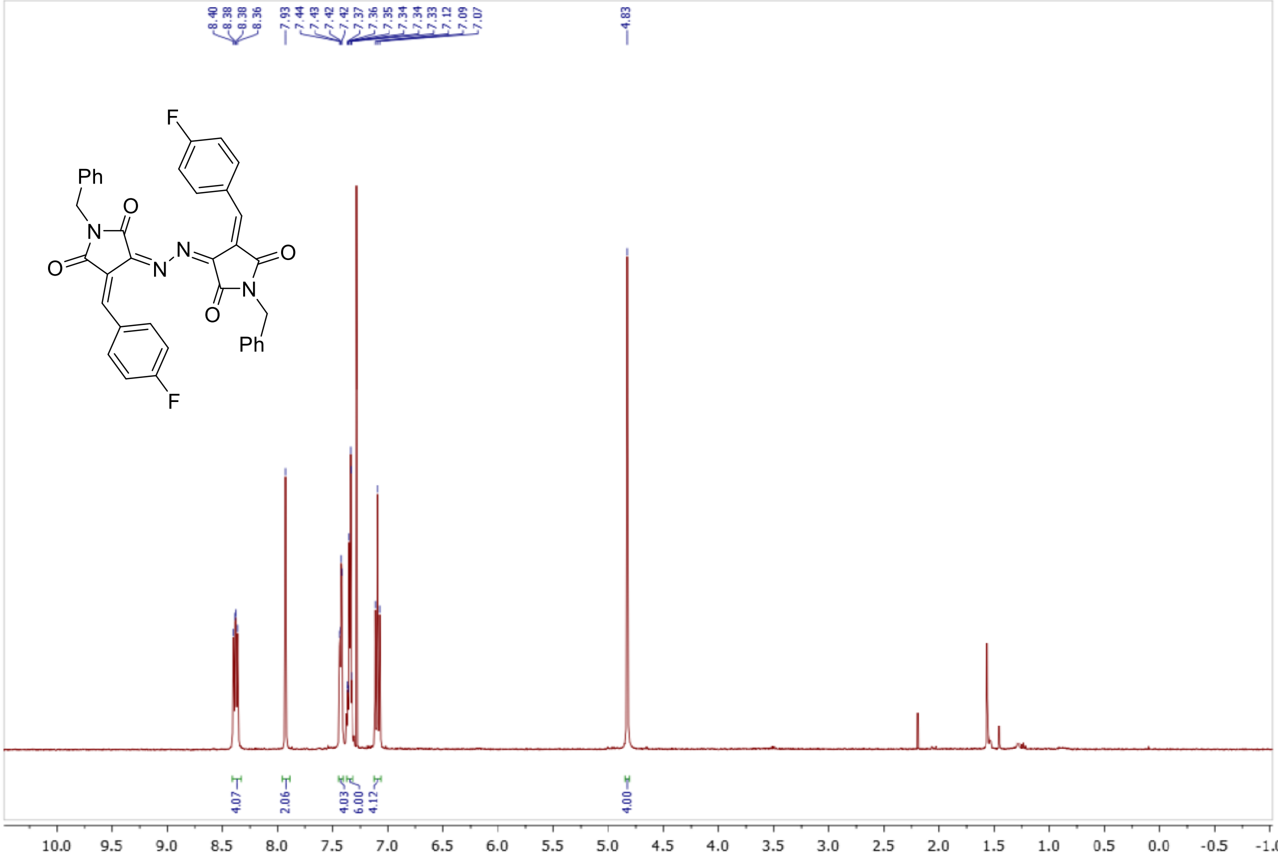

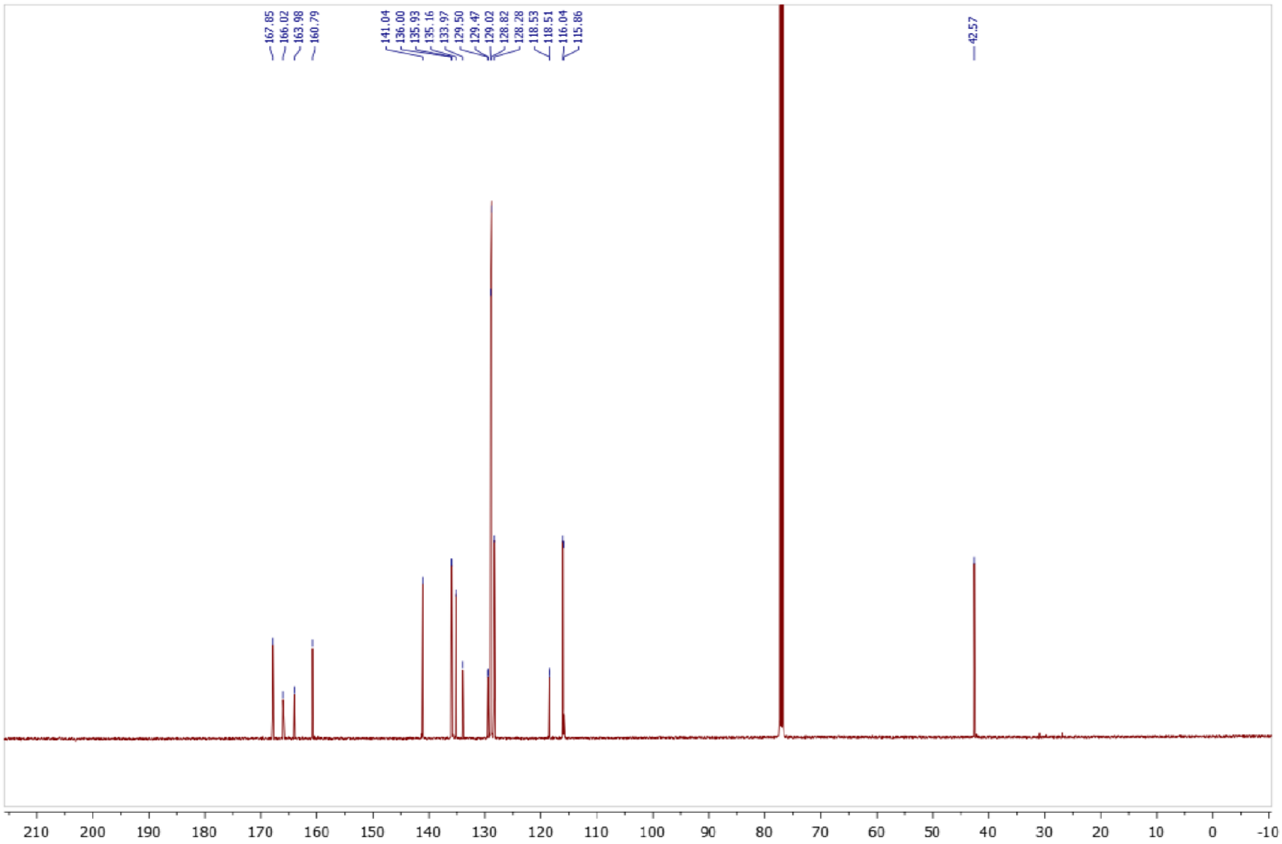

Copy of $^{19}F\{^1H\}$ (376.50 MHz, $CDCl_3$) spectrum of **4k**

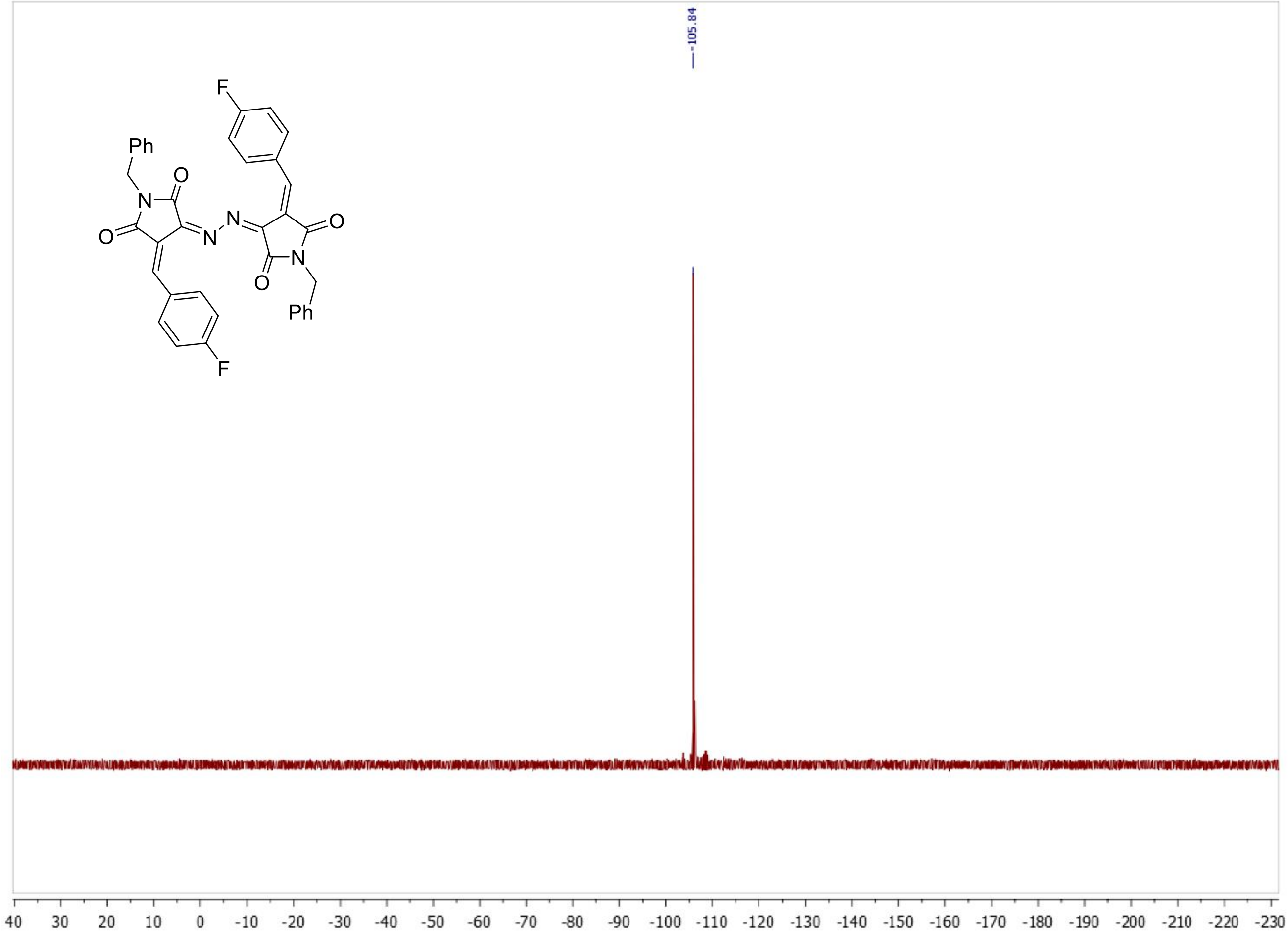

Copies of ^{1}H (400.13 MHz, $CDCl_3$) and $^{13}C\{^{1}H\}$ (100.61 MHz, $CDCl_3$) spectra of **2l**

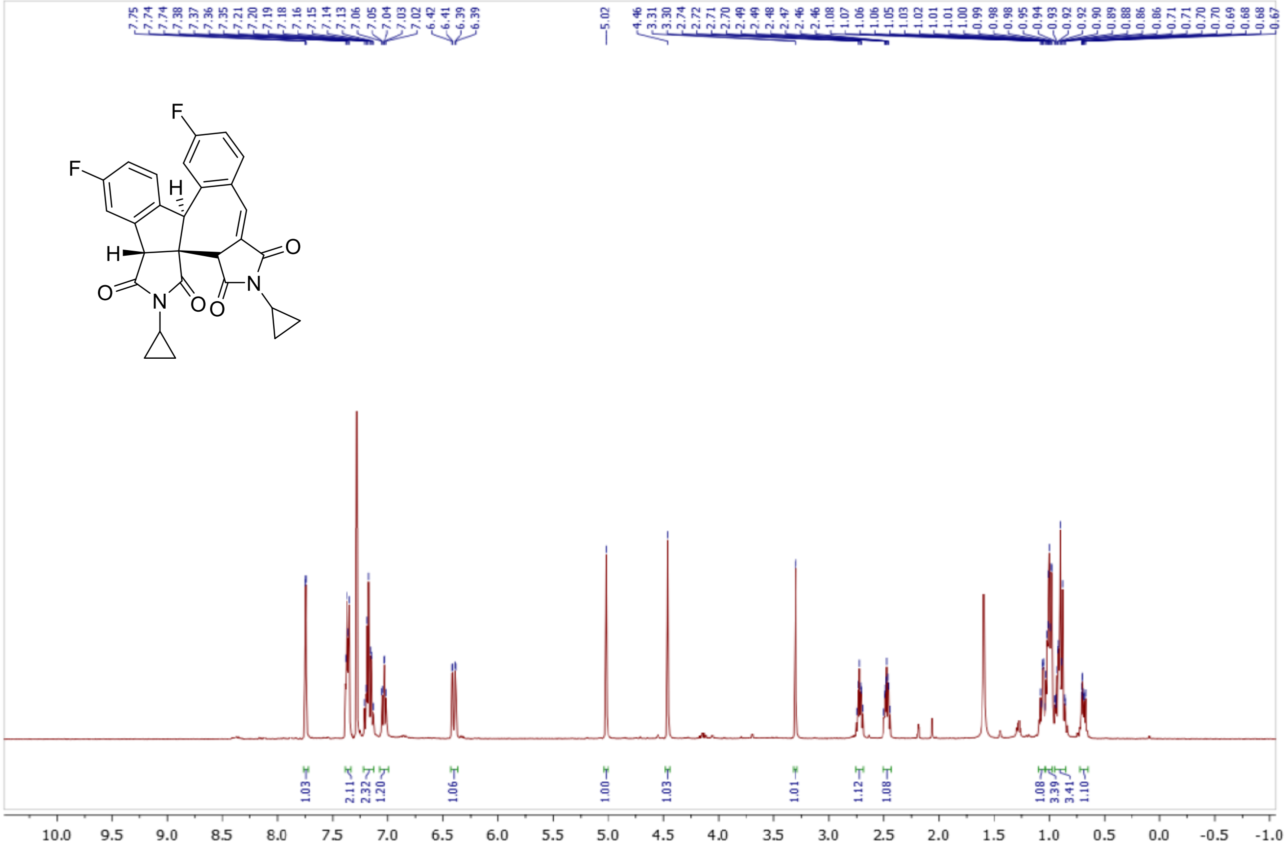

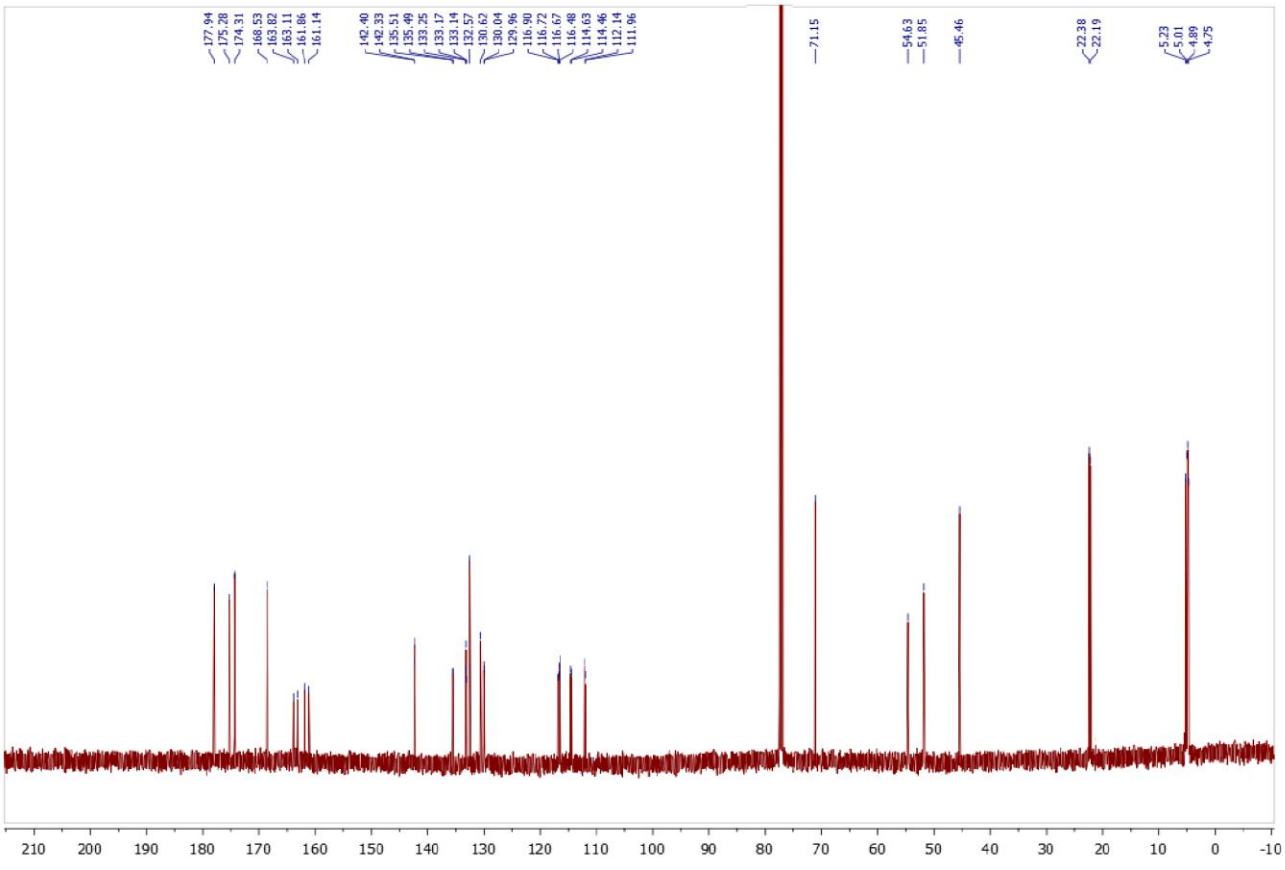

Copy of $^{19}F\{^{1}H\}$ (376.50 MHz, $CDCl_3$) spectrum of **2l**

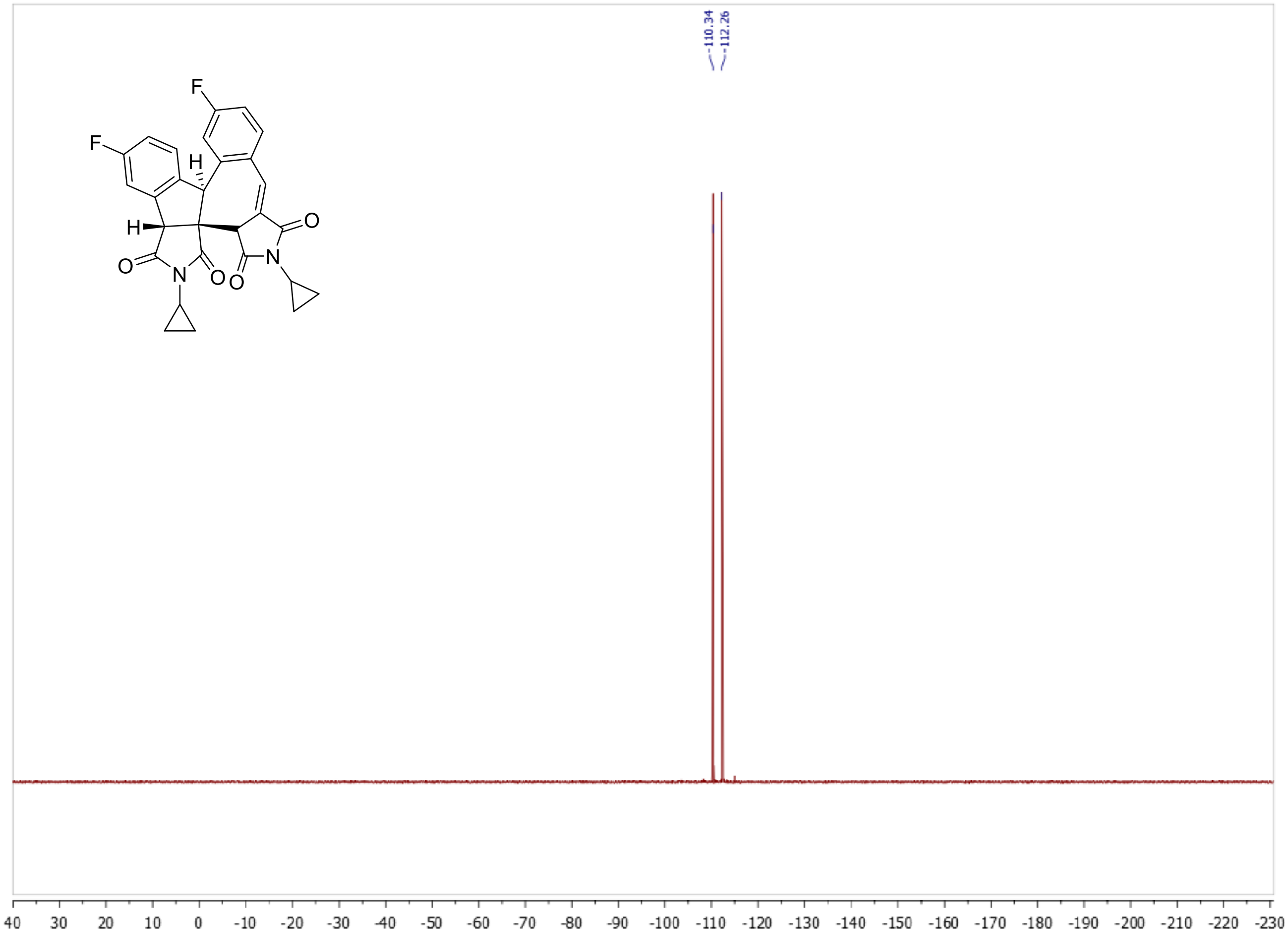

Copies of ^{1}H (400.13 MHz, $CDCl_3$) and $^{13}C\{^{1}H\}$ (100.61 MHz, $CDCl_3$) spectra of **2m**

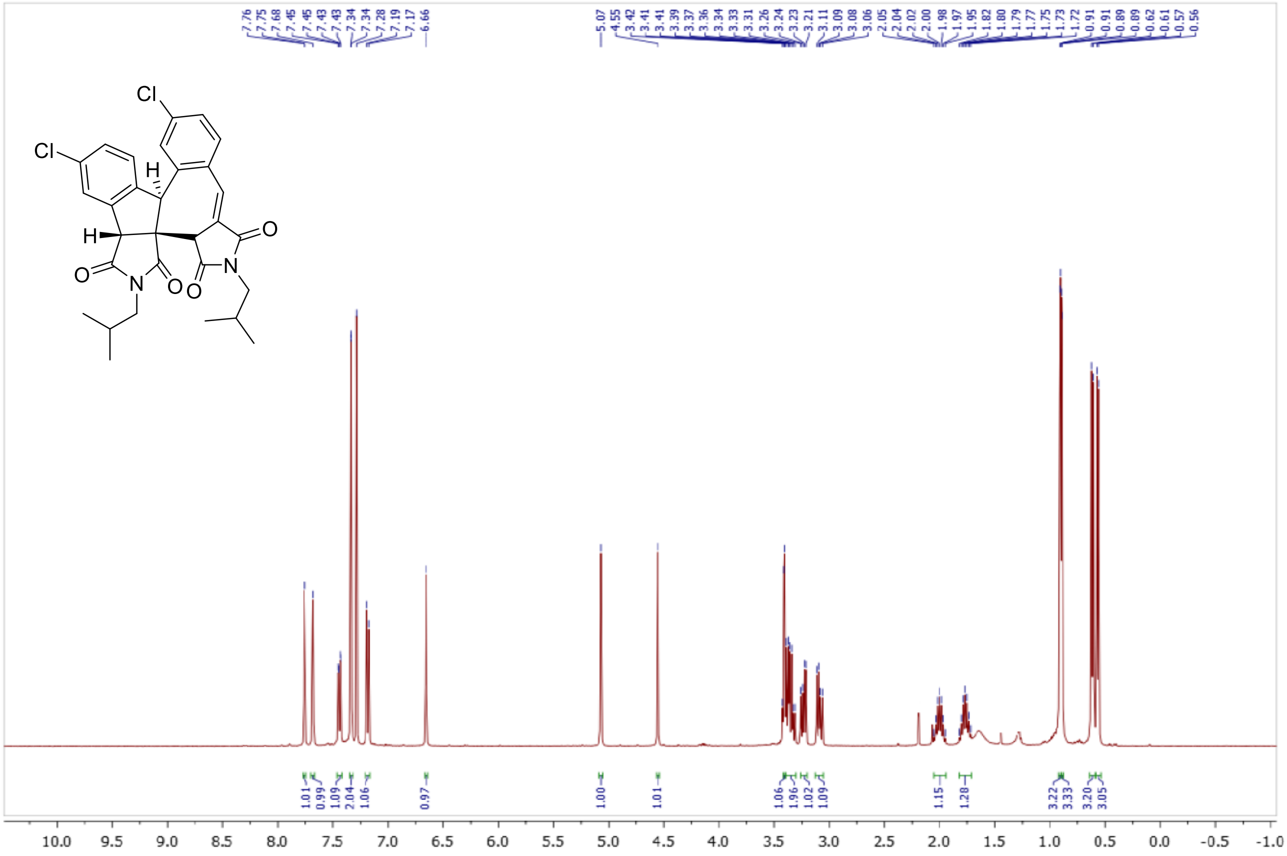

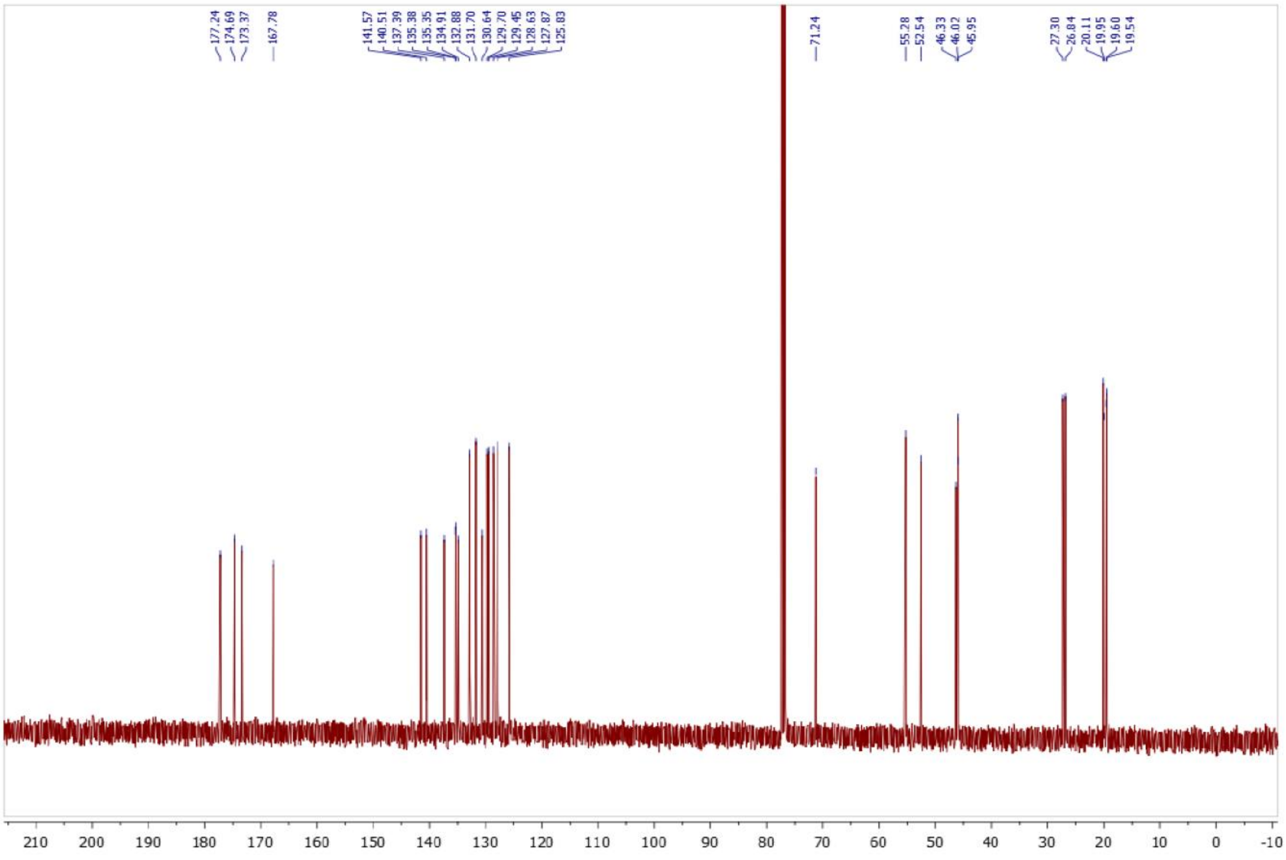

Copies of ^{1}H (400.13 MHz, $CDCl_3$) and $^{13}C\{^{1}H\}$ (100.61 MHz, $CDCl_3$) spectra of **2n**

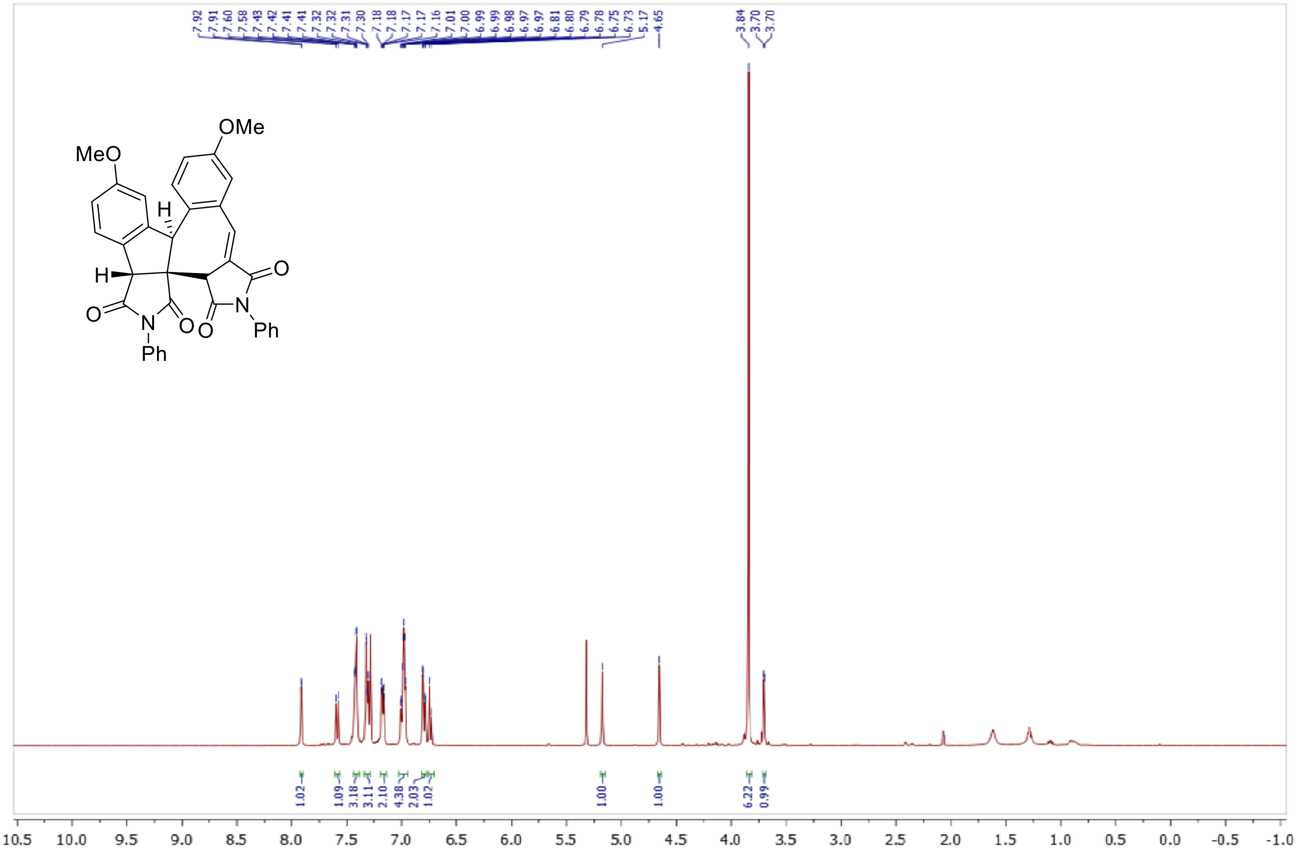

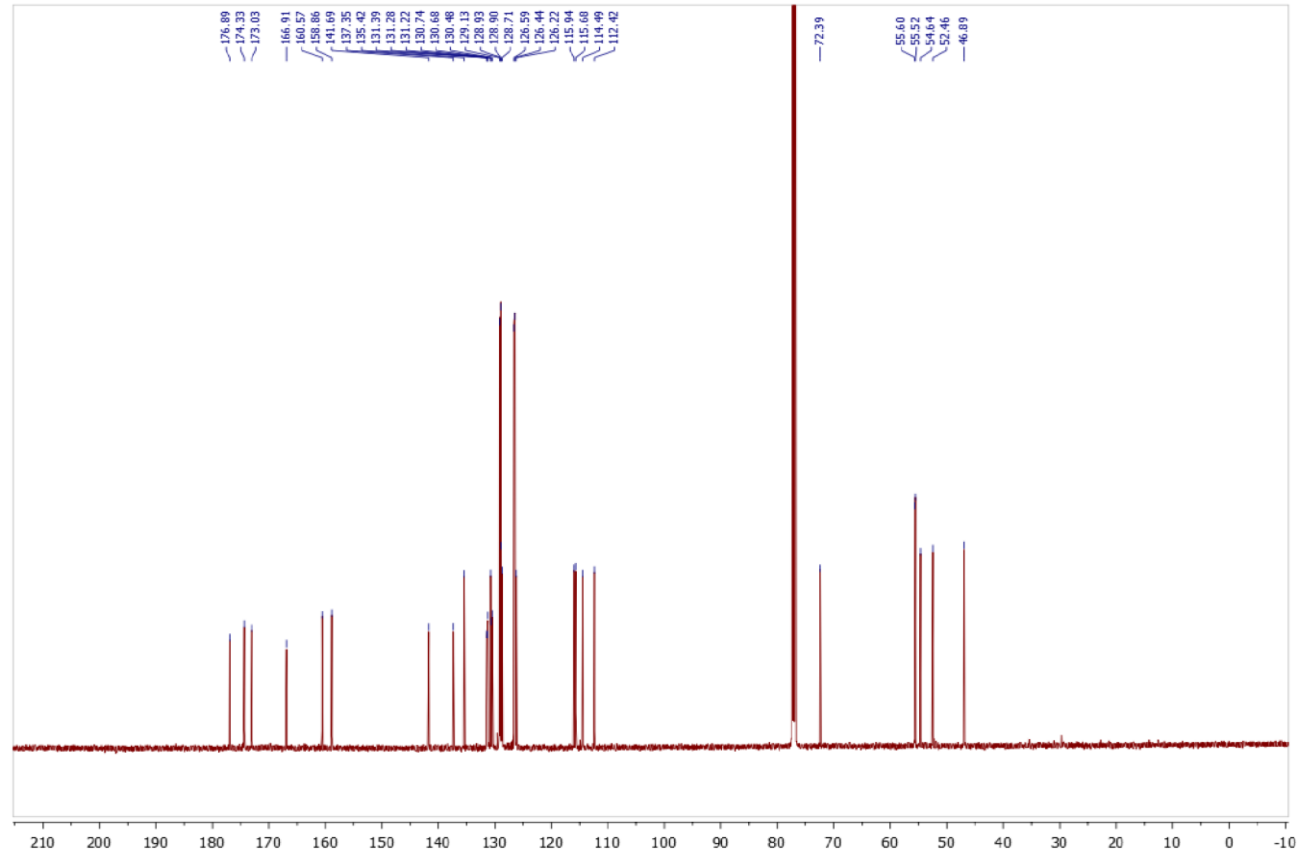

Copies of ^{1}H (400.13 MHz, $CDCl_3$) and $^{13}C\{^{1}H\}$ (100.61 MHz, $CDCl_3$) spectra of **3o**

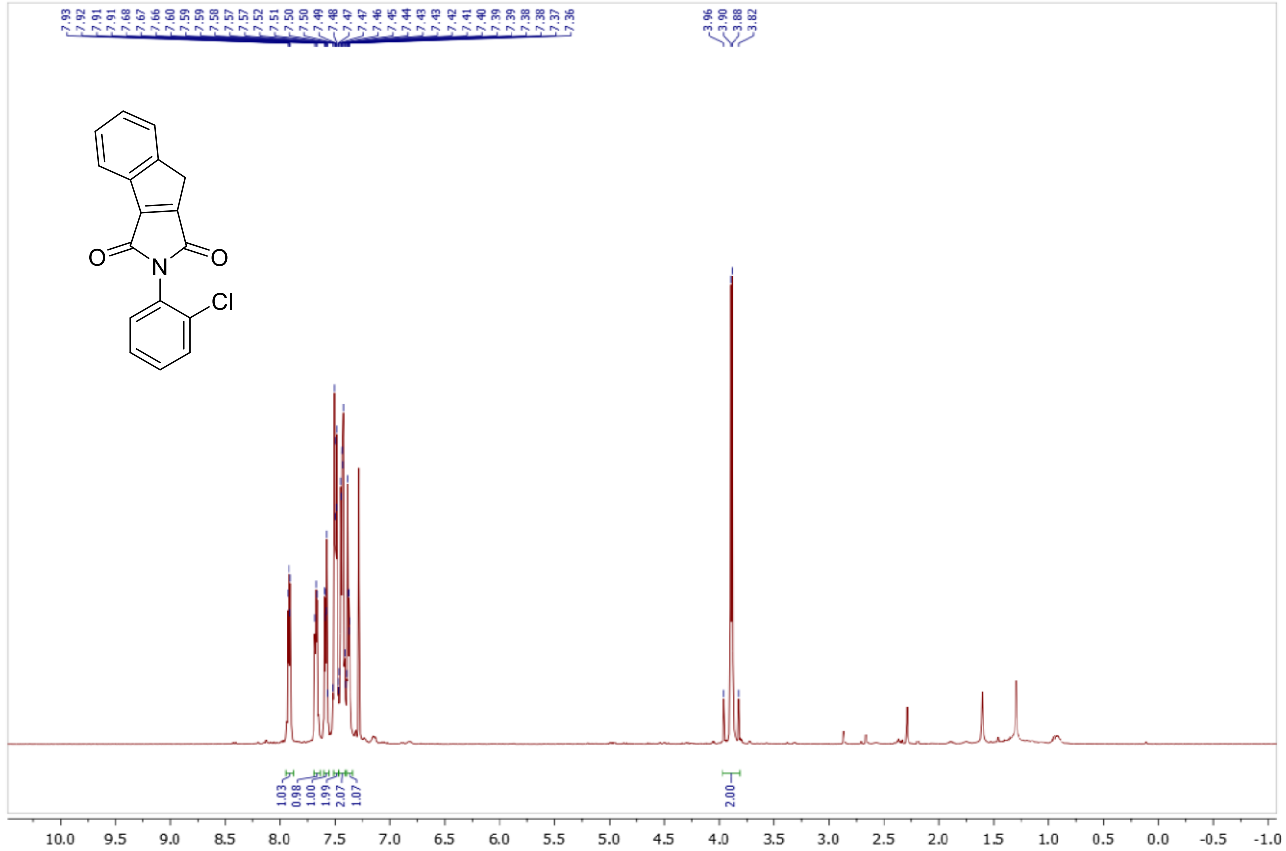

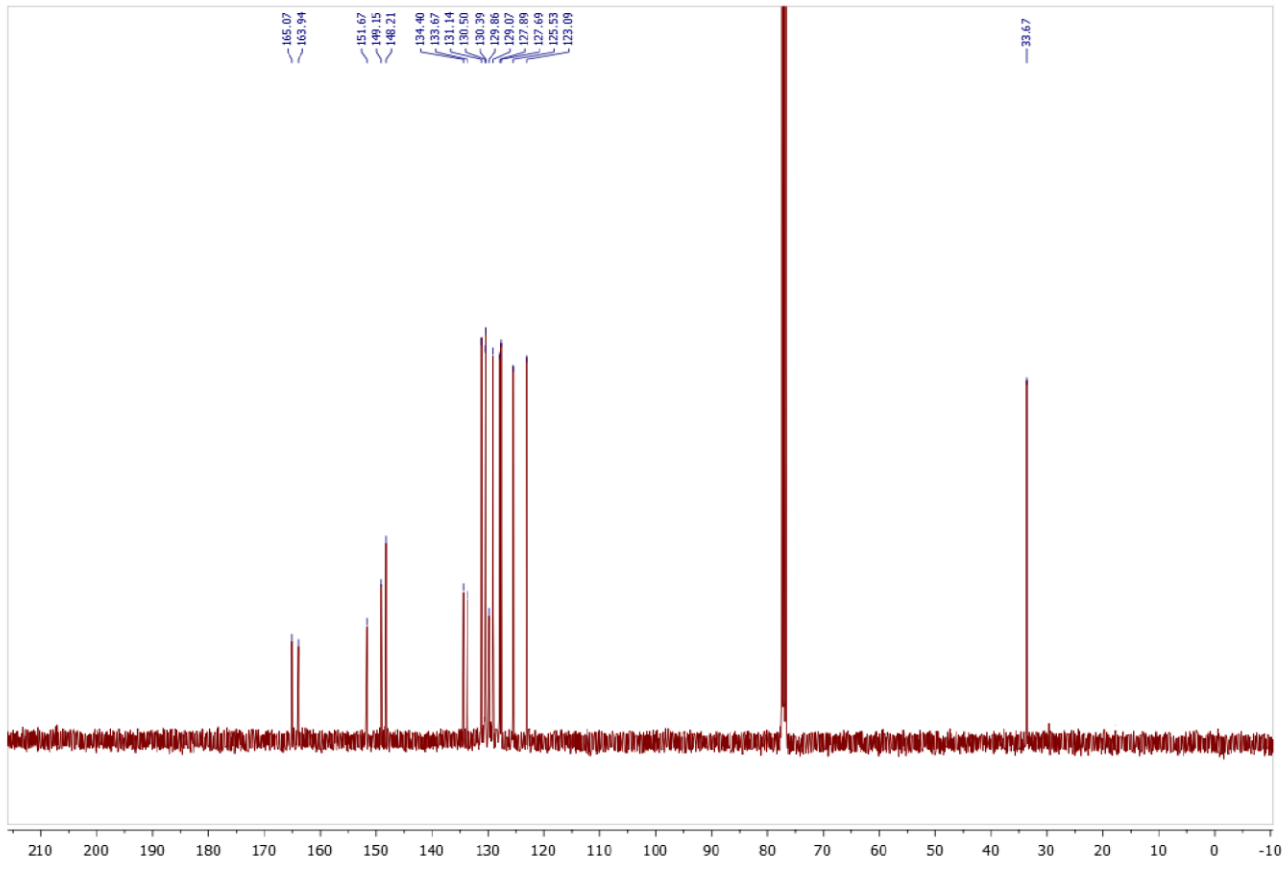

Copies of ^{1}H (400.13 MHz, $CDCl_3$) and $^{13}C\{^{1}H\}$ (100.61 MHz, $CDCl_3$) spectra of **3p**

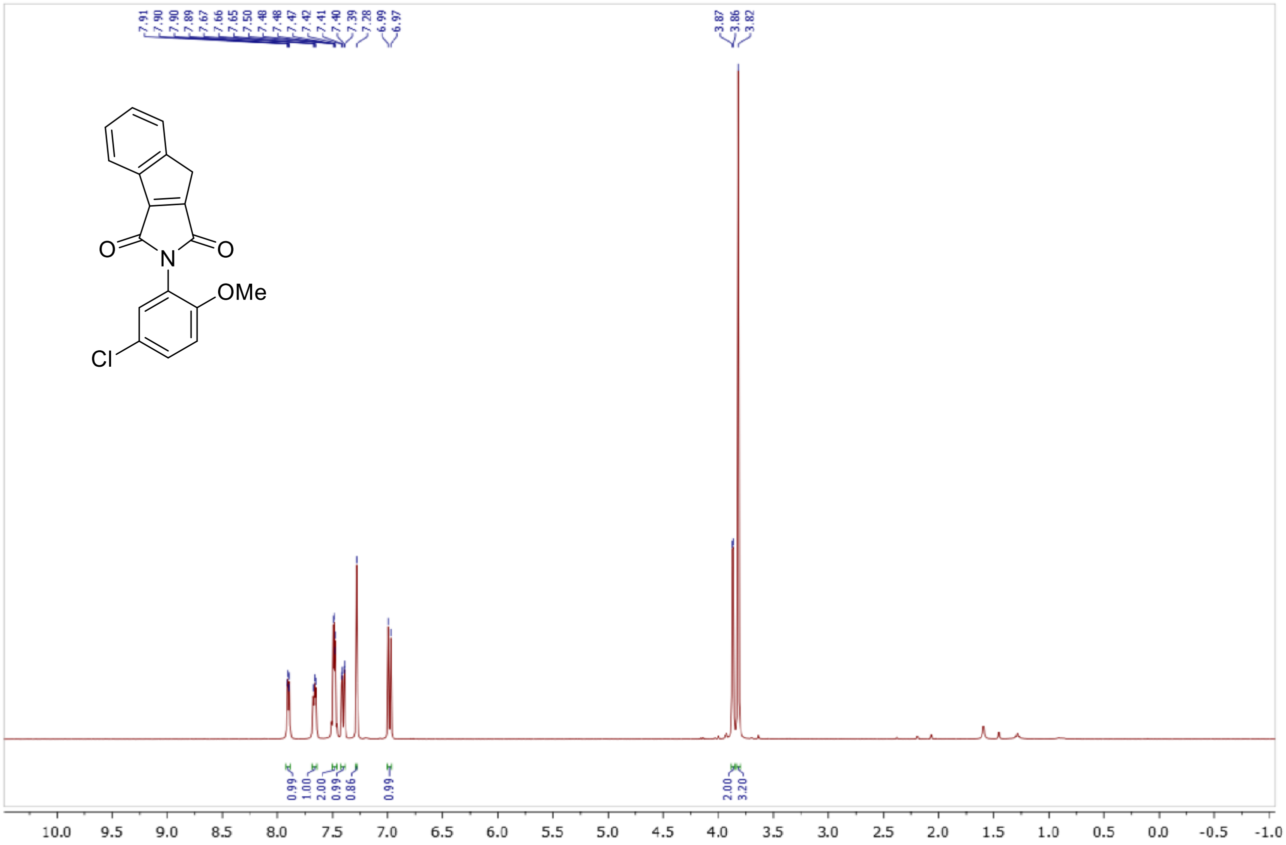

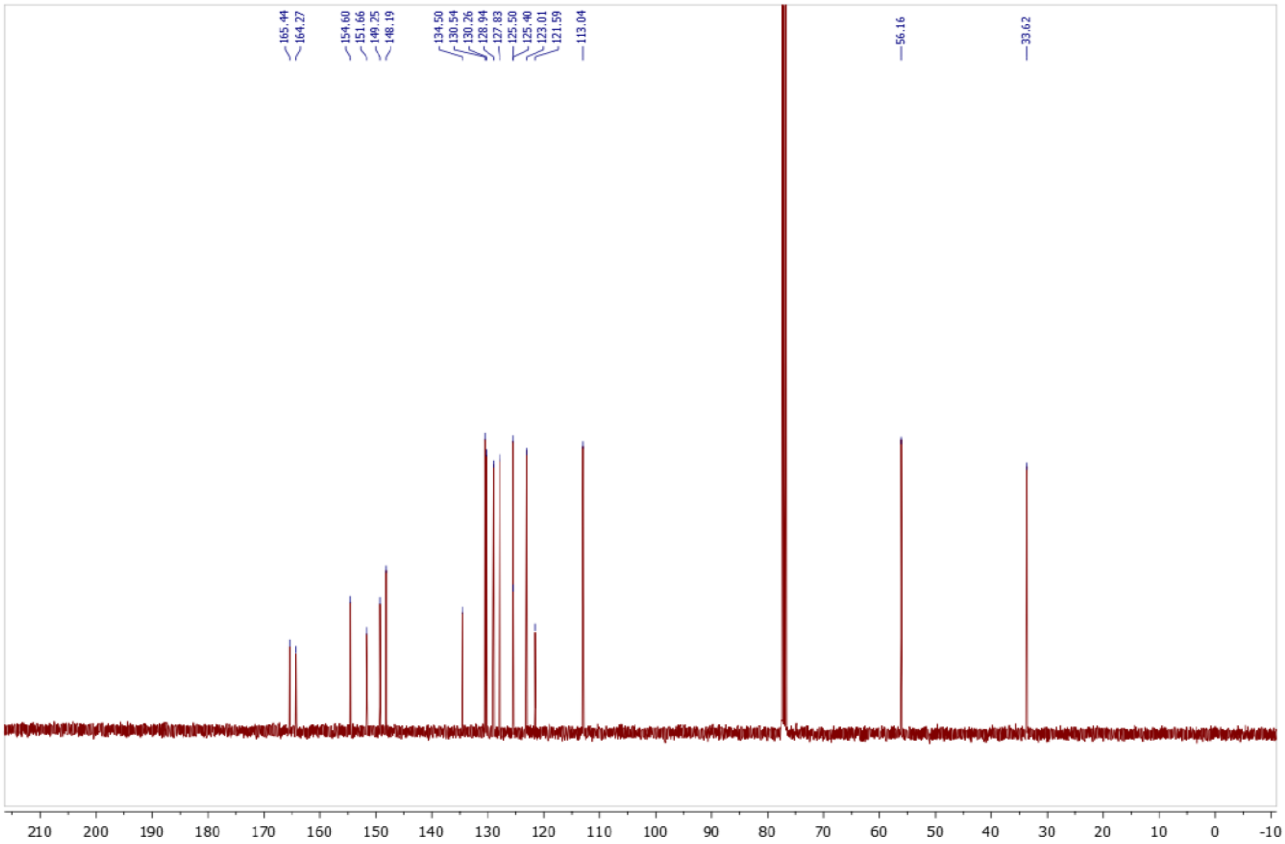

Copies of ^{1}H (400.13 MHz, $CDCl_3$) and $^{13}C\{^{1}H\}$ (100.61 MHz, $CDCl_3$) spectra of **3q**

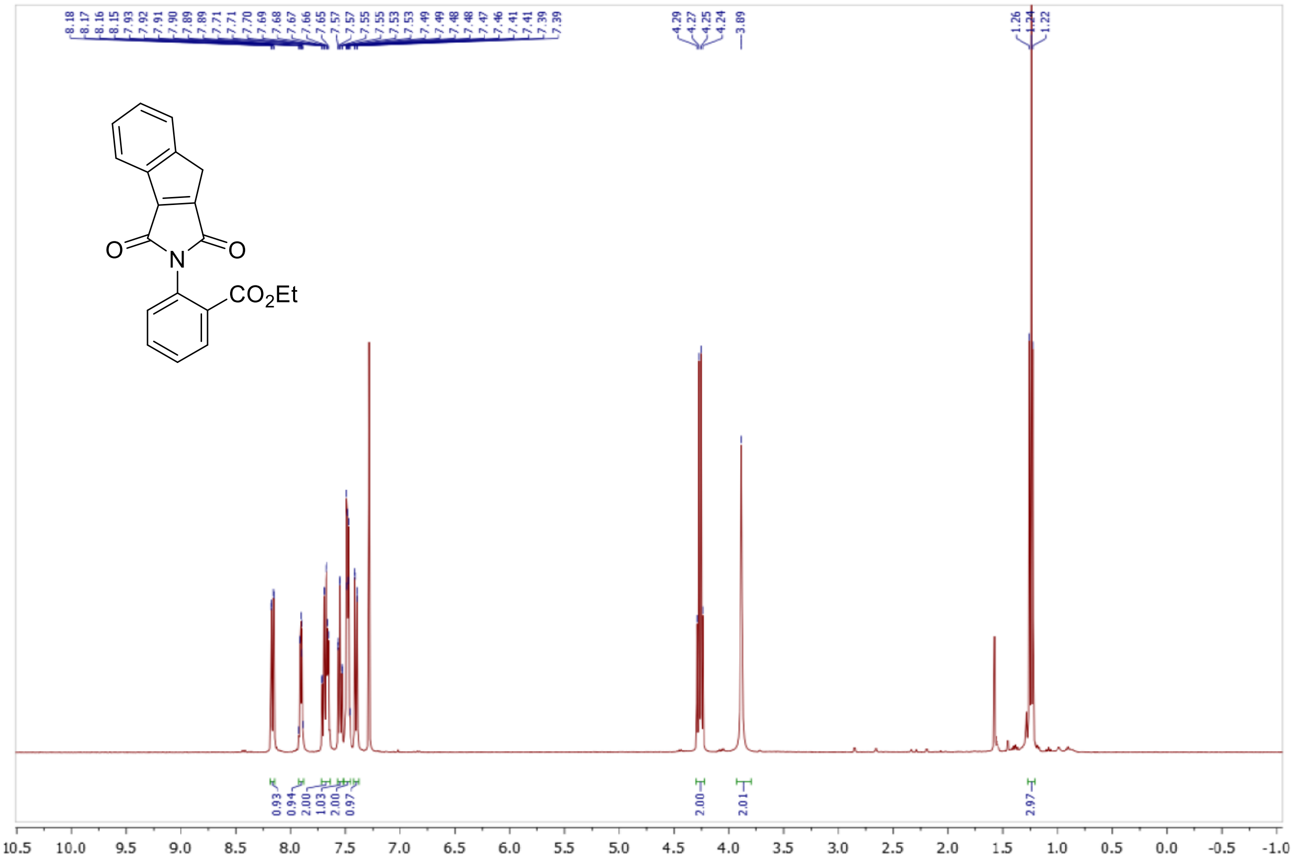

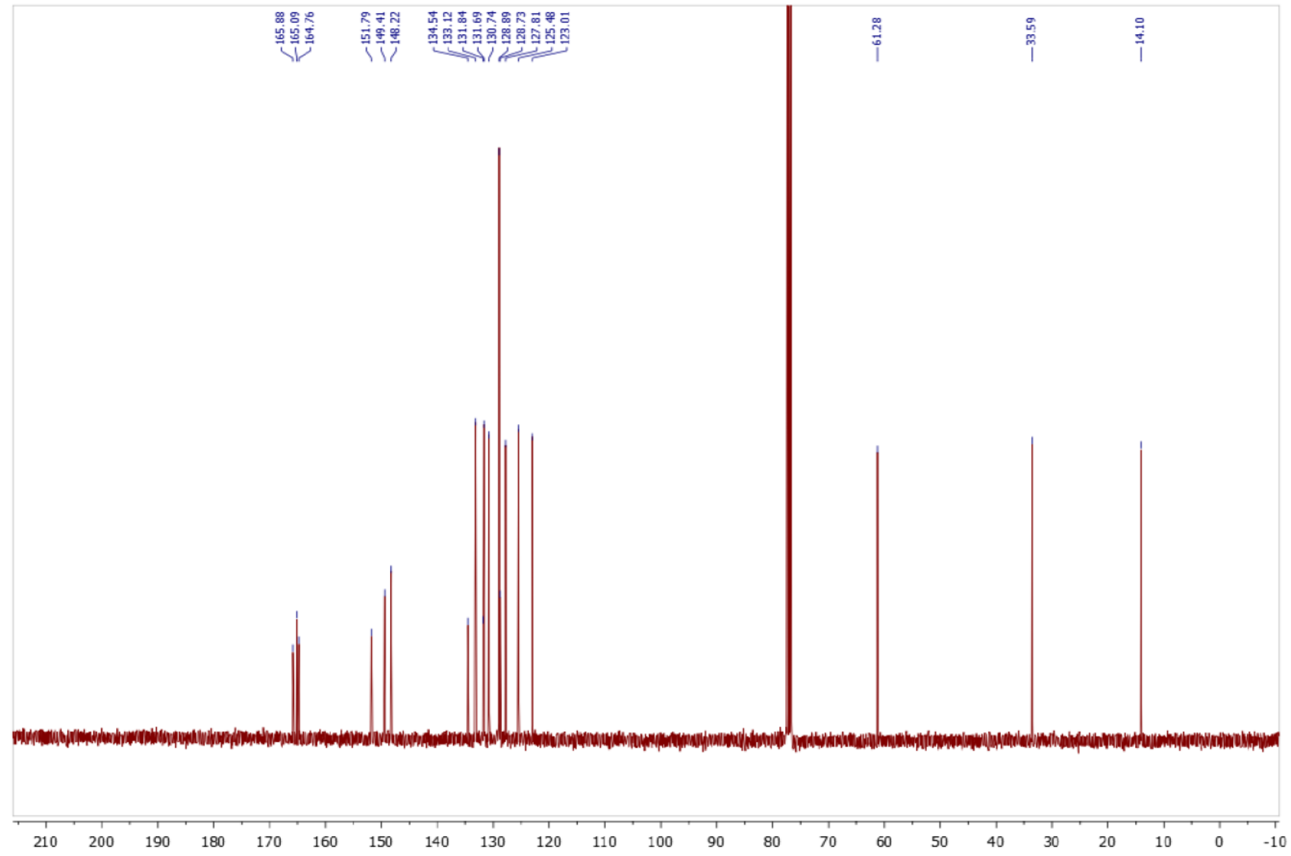

Copies of ^{1}H (400.13 MHz, $CDCl_3$) and $^{13}C\{^{1}H\}$ (100.61 MHz, $CDCl_3$) spectra of **2r**

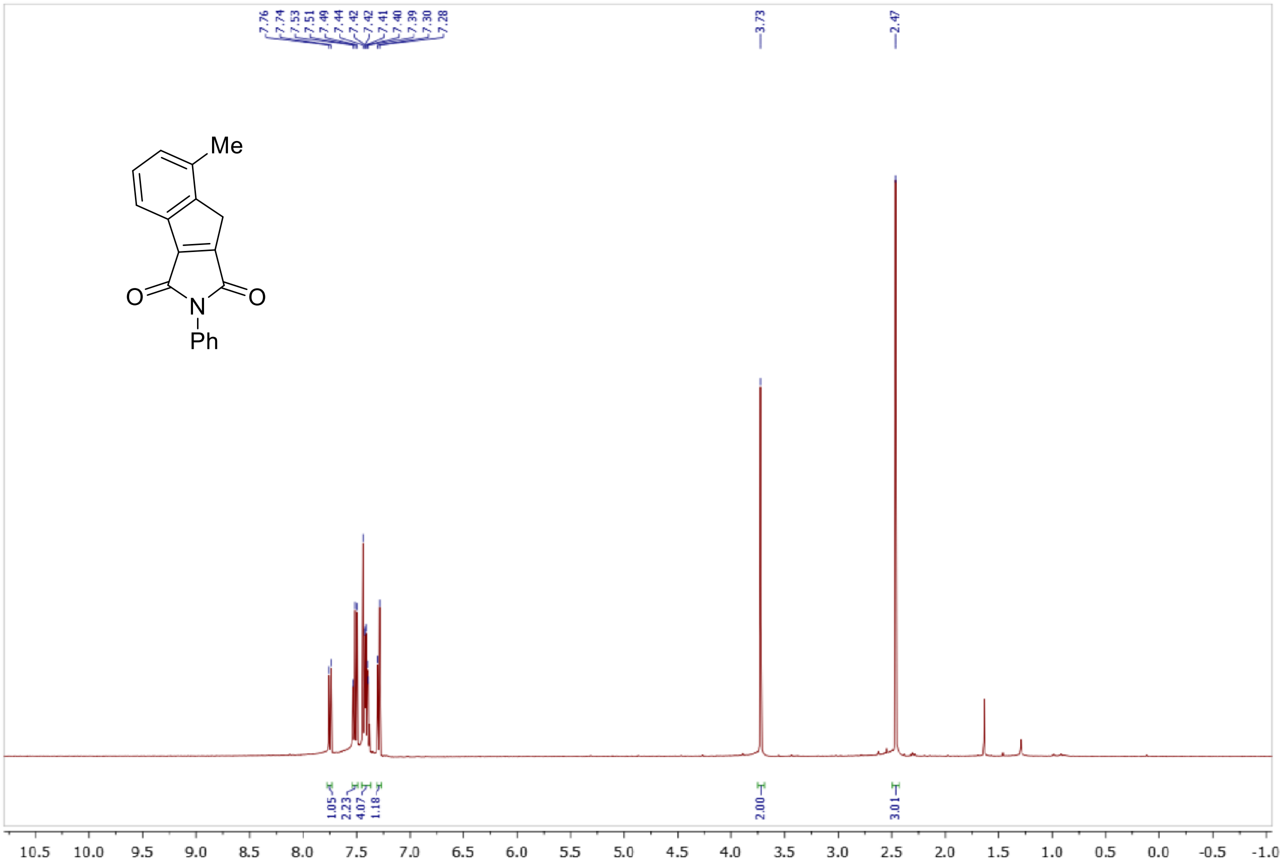

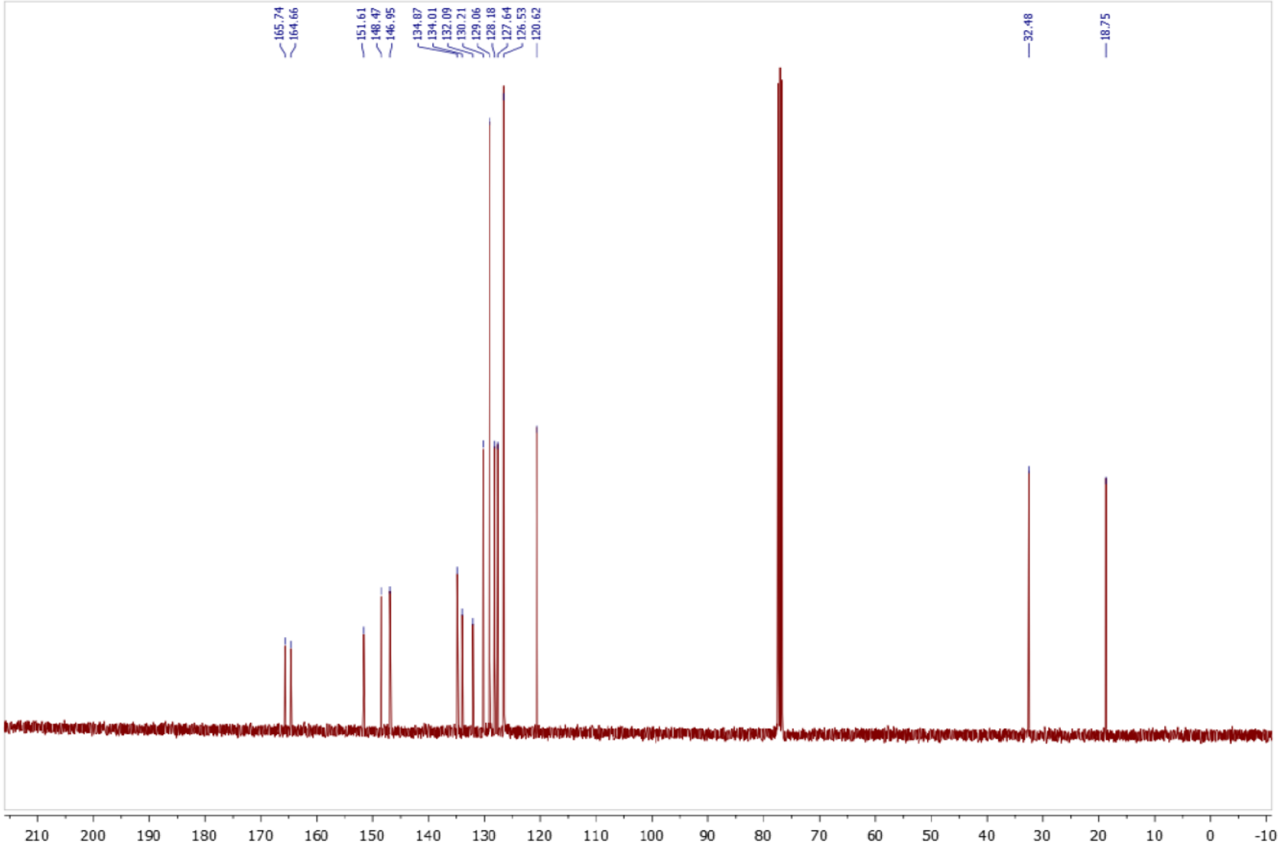

Copies of ^{1}H (400.13 MHz, $CDCl_3$) and ^{13}C{^{1}H} (100.61 MHz, $CDCl_3$) spectra of **3r**

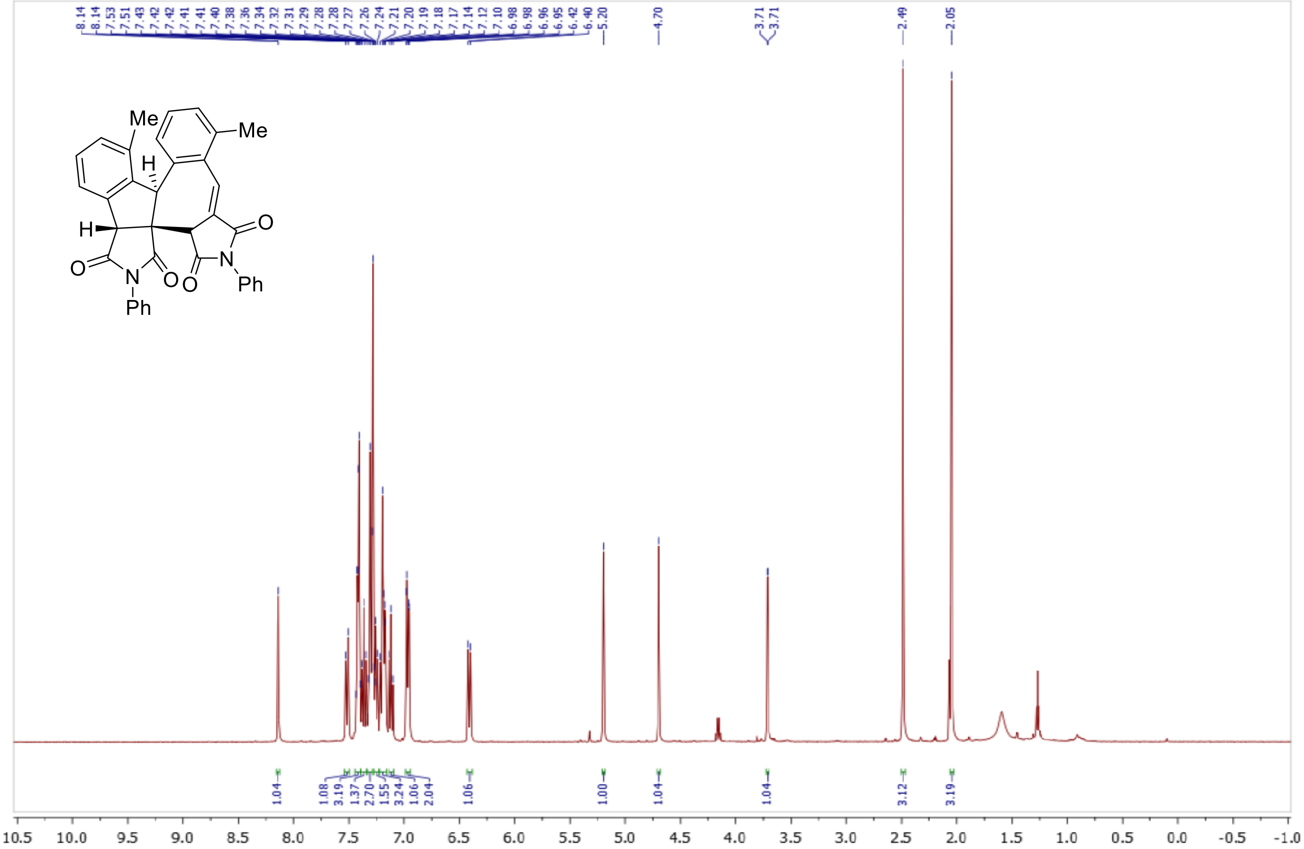

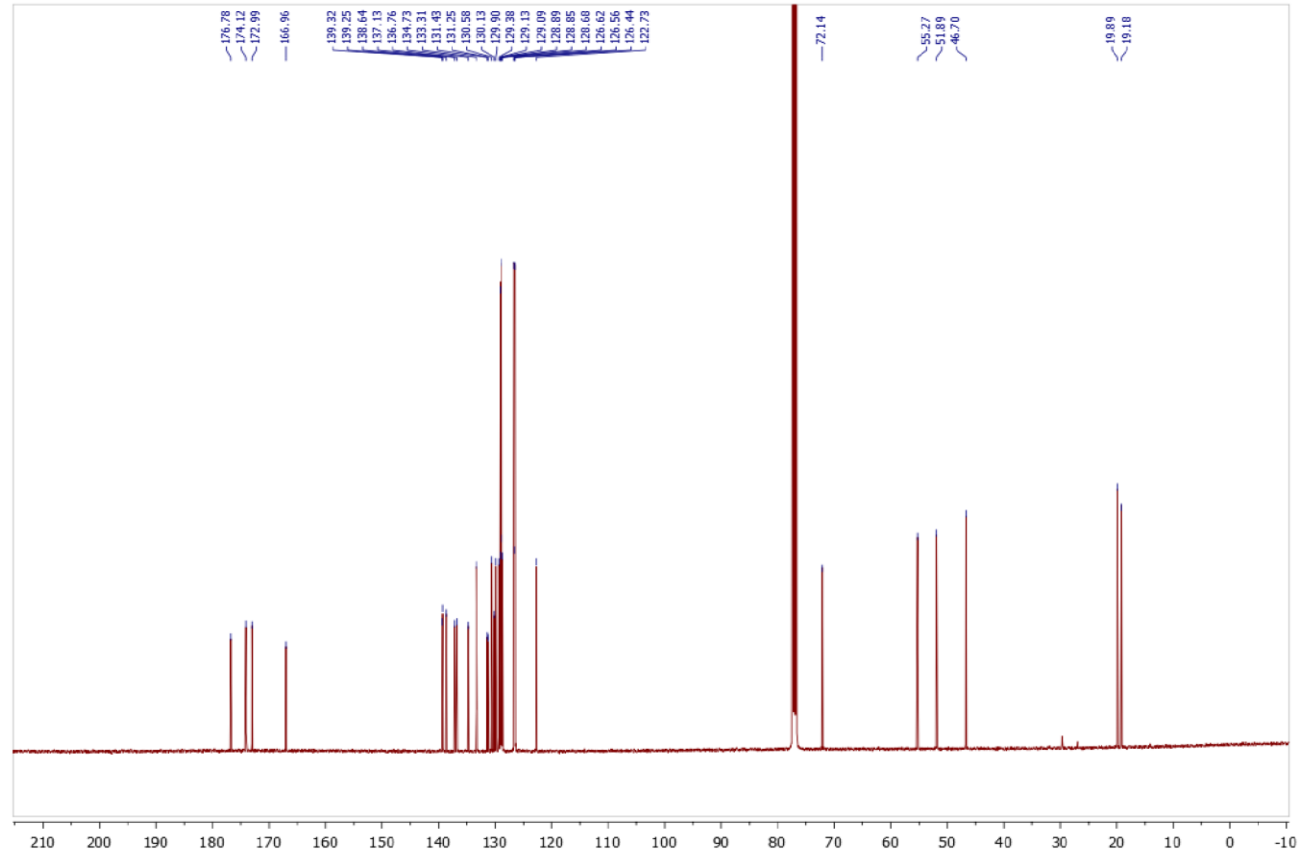

Copies of ^{1}H (400.13 MHz, $CDCl_3$) and $^{13}C\{^{1}H\}$ (100.61 MHz, $CDCl_3$) spectra of **5**

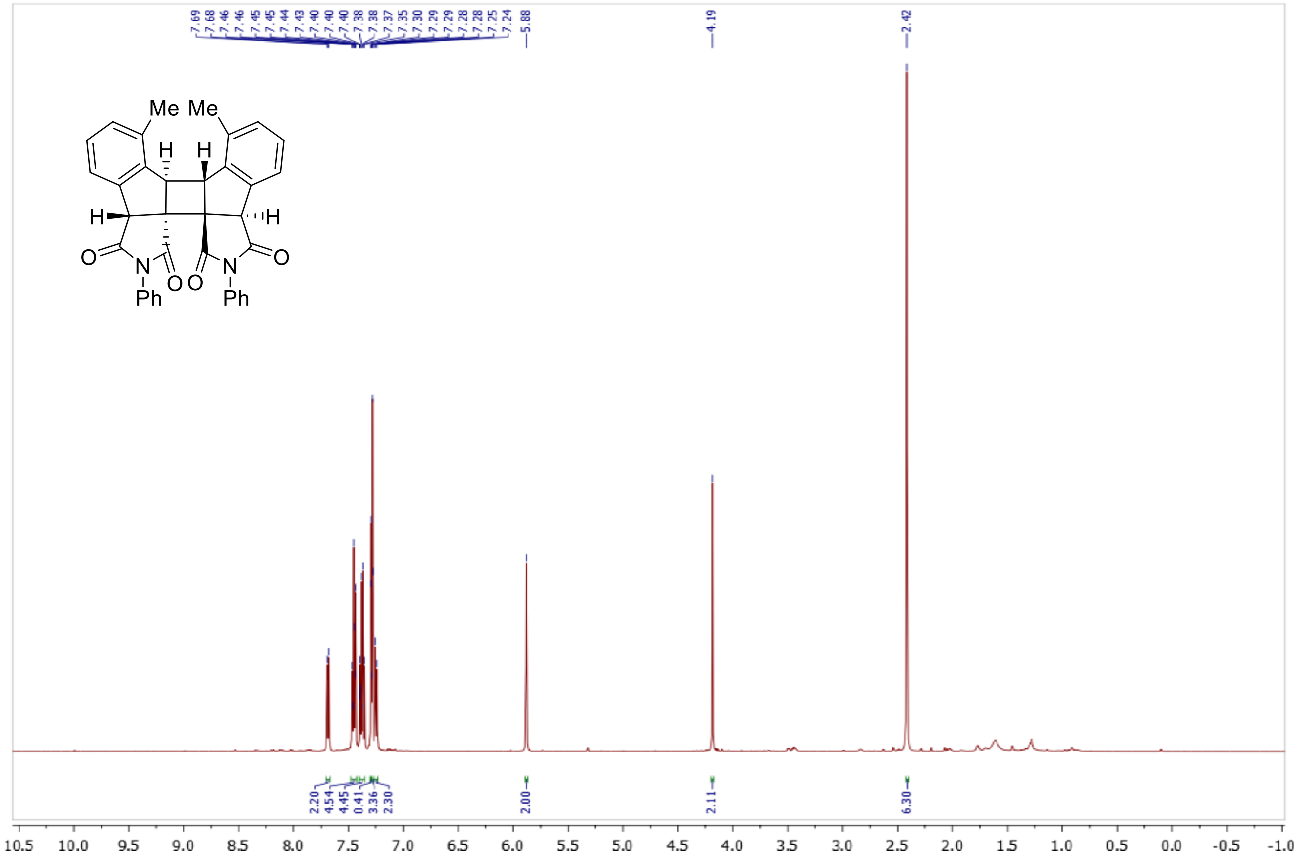

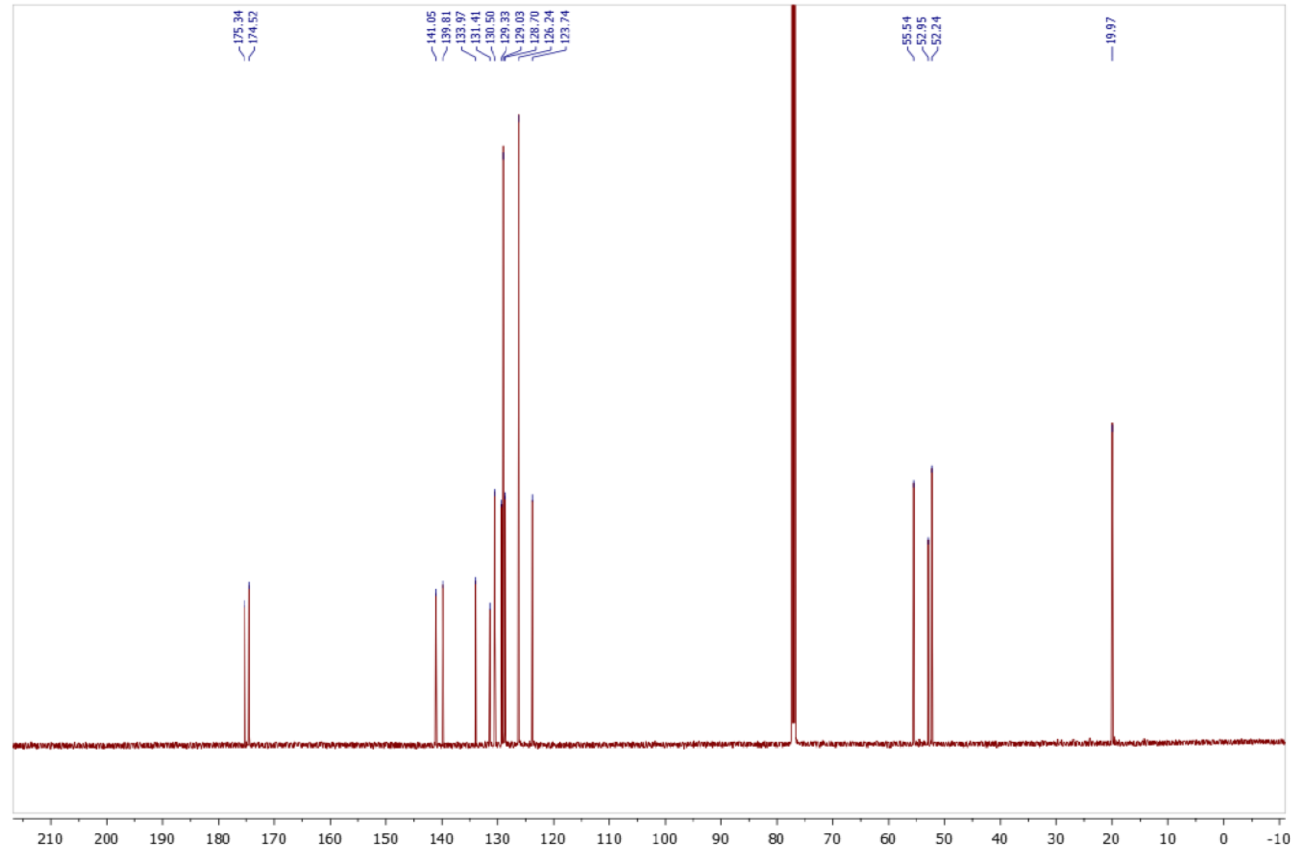

Supplement: File 1 — General experimental information, X-ray crystallographic data, synthetic procedures, analytical data and NMR spectra for the reported compounds. [file Beilstein_J_Org_Chem-18-533-s001.pdf]
